# Human versus equine intramuscular antitoxin, with or without human intrathecal antitoxin, for the treatment of adults with tetanus: a 2 × 2 factorial randomised controlled trial

**DOI:** 10.1016/S2214-109X(22)00117-6

**Published:** 2022-05-10

**Authors:** Nguyen Van Hao, Huynh Thi Loan, Lam Minh Yen, Evelyne Kestelyn, Duc Du Hong, Duong Bich Thuy, Nguyen Thanh Nguyen, Ha Thi Hai Duong, Tran Thi Diem Thuy, Phung Tran Huy Nhat, Phan Nguyen Quoc Khanh, Nguyen Thi Phuong Dung, Nguyen Hoan Phu, Nguyen Thanh Phong, Pham Thi Lieu, Pham Thi Tuyen, Bui Thi Bich Hanh, Ho Dang Trung Nghia, Pham Kieu Nguyet Oanh, Phan Vinh Tho, Tran Tan Thanh, Hugo C Turner, H Rogier van Doorn, Le Van Tan, Duncan Wyncoll, Nicholas PJ Day, Ronald B Geskus, Guy E Thwaites, Nguyen Van Vinh Chau, C Louise Thwaites

**Affiliations:** a Hospital for Tropical Diseases, Ho Chi Minh City, Vietnam; b University of Medicine and Pharmacy, Ho Chi Minh City, Vietnam; c Oxford University Clinical Research Unit, Hospital for Tropical Diseases, Ho Chi Minh City, Vietnam; d Pham Ngoc Thach University of Medicine, Ho Chi Minh City, Vietnam; e MRC Centre for Global Infectious Disease Analysis, School of Public Health, Imperial College London, London, UK; f Centre for Tropical Medicine and Global Health, Nuffield Department of Medicine Research Building, University of Oxford, Oxford, UK; g Guy's and St Thomas’ Hospitals NHS Trust, London, UK; h Mahidol Oxford Tropical Medicine Research Unit, Faculty of Tropical Medicine, Mahidol University, Bangkok, Thailand

## Abstract

**Background:**

Intramuscular antitoxin is recommended in tetanus treatment, but there are few data comparing human and equine preparations. Tetanus toxin acts within the CNS, where there is limited penetration of peripherally administered antitoxin; thus, intrathecal antitoxin administration might improve clinical outcomes compared with intramuscular injection.

**Methods:**

In a 2  × 2 factorial trial, all patients aged 16 years or older with a clinical diagnosis of generalised tetanus admitted to the intensive care unit of the Hospital for Tropical Diseases, Ho Chi Minh City, Vietnam, were eligible for study entry. Participants were randomly assigned first to 3000 IU human or 21 000 U equine intramuscular antitoxin, then to either 500 IU intrathecal human antitoxin or sham procedure. Interventions were delivered by independent clinicians, with attending clinicians and study staff masked to treatment allocations. The primary outcome was requirement for mechanical ventilation. The analysis was done in the intention-to-treat population. The study is registered at ClinicalTrials.gov, NCT02999815; recruitment is completed.

**Findings:**

272 adults were randomly assigned to interventions between Jan 8, 2017, and Sept 29, 2019, and followed up until May, 2020. In the intrathecal allocation, 136 individuals were randomly assigned to sham procedure and 136 to antitoxin; in the intramuscular allocation, 109 individuals were randomly assigned to equine antitoxin and 109 to human antitoxin. 54 patients received antitoxin at a previous hospital, excluding them from the intramuscular antitoxin groups. Mechanical ventilation was given to 56 (43%) of 130 patients allocated to intrathecal antitoxin and 65 (50%) of 131 allocated to sham procedure (relative risk [RR] 0·87, 95% CI 0·66–1·13; p=0·29). For the intramuscular allocation, 48 (45%) of 107 patients allocated to human antitoxin received mechanical ventilation compared with 48 (44%) of 108 patients allocated to equine antitoxin (RR 1·01, 95% CI 0·75–1·36, p=0·95). No clinically relevant difference in adverse events was reported. 22 (16%) of 136 individuals allocated to the intrathecal group and 22 (11%) of 136 allocated to the sham procedure experienced adverse events related or possibly related to the intervention. 16 (15%) of 108 individuals allocated to equine intramuscular antitoxin and 17 (16%) of 109 allocated to human antitoxin experienced adverse events related or possibly related to the intervention. There were no intervention-related deaths.

**Interpretation:**

We found no advantage of intramuscular human antitoxin over intramuscular equine antitoxin in tetanus treatment. Intrathecal antitoxin administration was safe, but did not provide overall benefit in addition to intramuscular antitoxin administration.

**Funding:**

The Wellcome Trust.

## Introduction

Despite several decades of sustained global vaccination programmes, Global Burden of Disease Study 2015 data indicate tetanus still causes 48 000–80 000 deaths every year, most of which occur in low-income and middle-income countries (LMICs).^1^ Tetanus is caused by a neurotoxin produced by *Clostridium tetani*, which, after retrograde axonal transport, inhibits CNS inhibitory synapses. The resultant skeletal muscle spasm and, in severe cases, autonomic nervous system dysfunction, require careful and complex supportive care, and antibiotics and antitoxin (toxin-neutralising antibody) are necessary to prevent further toxin release and neuronal uptake.

Tetanus antitoxin is one of the oldest therapeutic agents in infectious diseases, yet its optimal use in tetanus treatment remains uncertain. The introduction of prophylactic equine-origin antitoxin during World War 1 markedly reduced tetanus incidence among wounded soldiers.^2^ There are few studies that have formally evaluated the benefit of antitoxin in established disease but, in 1960, an open-label trial randomly assigned 79 adults and children in Nigeria and Jamaica to treatment with or without equine antitoxin (200 000 IU) and reported reduced mortality in individuals treated with antitoxin (49% *vs* 76%).^3^

**Research in context**

**Evidence before this study**

Intramuscular antitoxin is recommended for the treatment of tetanus. To identify the evidence concerning optimal antitoxin preparation and route of administration, searches of MEDLINE, PubMed, and Cochrane databases were conducted from database inception to Dec 31, 2017, using the terms "tetanus", "management", "antitoxin", "treatment", "intrathecal", and "immunoglobulin", and with no language restrictions. Tetanus sections in WHO, US Centres for Disease Control and Prevention, European Medicines Agency, and UK Health Protection Agency UK websites were also searched manually for "tetanus". Additional full-text publications were identified through citations within articles via a manual search. Human antitoxin is recommended for the treatment of tetanus, largely based on historical estimates of allergic reactions to equine antitoxin (estimated incidence of 2–50%). Only one trial comparing the human with equine preparations has been performed: a double-blind randomised controlled trial comparing intramuscular injection of 10 000 IU equine antitoxin with 500 IU human antitoxin in neonates in Haiti between 1969 and 1970, with no significant difference in outcomes. Animal studies at the beginning of the 20th century suggested that antitoxin injected directly into the CNS was associated with better outcomes in experimentally induced tetanus. Case series and small, low-quality randomised trials in humans have reported improvements in mortality and hospital stay in both adults and neonates treated with intrathecal antitoxin (human and equine preparations). Most of these studies were done more than 40 years ago and in settings without access to mechanical ventilation. Two meta-analyses have been done. Overall findings and subgroup analyses were conflicting and frequent methodological bias, lack of safety evidence, and possible publication bias were cited as important limitations with current evidence. Safety data were absent in most studies and only one study followed up patients after hospital discharge.

**Added value of this study**

Our study is one of the few clinical trials of antitoxin treatment in tetanus in which attending clinical and study staff were masked to treatment allocations, and systematic surveillance for adverse events and follow-up after hospital discharge occurred. Our study is, to our knowledge, the first randomised controlled trial comparing human and equine intramuscular antitoxin in adults. We found no evidence of differing efficacy between interventions and importantly we found no evidence of increased adverse events in those treated with equine antitoxin. The addition of intrathecal antitoxin did not substantially reduce requirement for mechanical ventilation in adults with tetanus. Intrathecal antitoxin administration was not associated with adverse safety events or detrimental to long-term outcome.

**Implications of all the available evidence**

Equine antitoxin is cheaper and, in many countries, more easily available than human antitoxin. Our findings have shown that intramuscular equine antitoxin is safe, particularly where human antitoxin is unavailable. The additional administration of intrathecal antitoxin, although safe, did not add substantial benefit to outcomes in a setting with good access to mechanical ventilation and low mortality. The small reduction in mechanical ventilation rate we observed was similar to that observed in a smaller, well conducted randomised controlled trial in a Brazilian intensive care unit. Overall, there remains insufficient evidence to recommend the routine use of intrathecal antitoxin in the treatment of adults with tetanus.

Specific human antitoxin (human tetanus immunoglobulin) became available in the 1960s. Only one randomised controlled trial^4^ compared intramuscular human (500 units) with equine tetanus antitoxin (10 000 units), finding no difference in mortality or adverse events in 130 enrolled neonates. Similarly, no outcome differences were seen in an observational study^5^ of 386 adults with tetanus in the USA receiving variable doses of human and equine antitoxins, but not reporting adverse event data. Despite the absence of data demonstrating the superiority of human antitoxin, concerns over adverse reactions to equine antitoxin have led most guidelines to recommend human preparations.^6^, ^7^ In high-income settings, short supply has necessitated modification of guidelines to allow substitution of human antitoxin with intravenous immunoglobulins (IVIg) without supporting clinical evidence.^6^, ^7^, ^8^ In resource-limited settings, cost and availability limit the use of both human antitoxin and IVIg.

Early 20th century reports showed that animals with experimentally induced tetanus had better outcomes if antitoxin was given intrathecally rather than peripherally.^9^ The first therapeutic use of intrathecal tetanus antitoxin in humans was more than 100 years ago,^10^ but this route of administration was largely neglected until the late 1970s.^11^, ^12^, ^13^, ^14^ A meta-analysis including 942 patients from 12 trials of both human and equine intrathecal antitoxin reported that intrathecal administration reduced mortality (relative risk of death 0·71, 95% CI 0·62–0·81); however, only two included trials attempted to conceal the treatment allocation from assessing doctors^15^, ^16^ and safety data were mostly unreported. Since most of these studies were done, survival rates from tetanus have significantly improved because critical care facilities, particularly mechanical ventilation, have become more widely available.^17^, ^18^ Nevertheless, tetanus remains a significant burden on LMIC health systems, with patients commonly requiring prolonged periods in the intensive care unit (ICU).^19^ There are, however, little data on the impact of antitoxin on length of ICU stay and requirement for organ support (particularly mechanical ventilation).

Due to the continuing uncertainties about the best preparation and route of administration of antitoxin in tetanus, we carried out a 2  × 2 factorial study in adults with tetanus to compare current standard of care in Vietnam (intramuscular equine antitoxin) with human antitoxin, with or without additional intrathecal human antitoxin administration. Although our primary objective was to establish whether the addition of intrathecal tetanus antitoxin reduces the need for mechanical ventilation in patients with tetanus, our study also aimed to examine impact on other markers of disease severity and provide data to inform the recommendation of human rather than equine antitoxin, although it was not powered to detect this difference.

## Methods

### Trial design

The study was a prospective 2  × 2 factorial single-blinded randomised controlled trial. The study was conducted at the Hospital for Tropical Diseases, Ho Chi Minh City, Vietnam—a specialist referral hospital for infectious diseases, admitting approximately 250–350 adults with tetanus from southern Vietnam each year.

The protocol was approved by the Ethical Committee of the Hospital for Tropical Diseases, Ho Chi Minh City, Vietnam; the Oxford Tropical Research Ethics Committee, Oxford, UK; and the Ministry of Health, Vietnam.^20^ The protocol was modified after recruitment of the first 67 patients to allow individuals treated with intramuscular antitoxin at their previous hospital to be enrolled.^20^

### Participants

All patients aged 16 years or older with a clinical diagnosis of generalised tetanus admitted to the hospital's ICU were eligible for study entry. Generalised tetanus was diagnosed according to the Hospital for Tropical Disease guidelines: a clinical diagnosis with characteristic features of trismus, dysphagia, and continuous generalised muscle rigidity or spasms in the presence of normal conscious level and without fever at onset.^21^ Exclusion criteria were contraindication to use of human or equine antitoxin, uncertainty about previous antitoxin treatment, contraindication to lumbar puncture, already receiving mechanical ventilation or expected to require this before intrathecal injection could be given, and pregnancy.

All participants or their representatives gave written informed consent before enrolment in the study.

### Randomisation and masking

This was a 2  × 2 factorial trial; thus, two randomisations were done—one for intramuscular interventions and another for intrathecal interventions. A computer-based randomisation list was used to generate the sequence with which participants were allocated to comparison groups. Randomisation was restricted using block randomisation with variable block lengths of 8 and 12 without stratification. For the intramuscular intervention, participants were randomly assigned (1:1) to receive either equine or human intramuscular antitoxin. For the intrathecal intervention, participants were randomly assigned (1:1) to receive either a sham procedure or intrathecal antitoxin. All care providers (ICU doctors and nurses) and study staff were masked to the treatment allocation. Only the study pharmacist who was not otherwise involved in the trial had access to the randomisation list and used it to prepare externally, sequentially numbered, identical treatment packages containing the appropriate study intervention. Attending ICU doctors screened and enrolled eligible patients before informing an independent team of doctors and nurses from a different department who opened the treatment packages and delivered the allocated interventions. All interventions were given behind screens to conceal the intervention from ICU and study staff. No record of the treatment allocation was made in the patients’ records and all treatment packages, empty or residual antitoxin vials, and cerebrospinal fluid aliquots were removed from the ICU directly after the procedure by the independent team. Used treatment packages and drugs were processed appropriately by the study pharmacist, maintaining masking to the study team and ICU staff.

### Procedures

The trial followed the protocol used in a previously published pilot phase.^22^ For the intramuscular intervention, participants were randomly assigned to either equine intramuscular antitoxin (21 000 IU, Vien Vaccin & SPYT, Nha Trang, Vietnam) or human intramuscular antitoxin (3000 IU Tetagam P, CSL Behring, Marburg, Germany). The intramuscular treatment was omitted in patients who had received a therapeutic dose of intramuscular antitoxin at a previous hospital. At the time of the study, only equine intramuscular antitoxin was available for routine care in Vietnam. For the intrathecal intervention, participants were randomly assigned to receive either a sham procedure or 500 IU Tetagam P given by intrathecal injection.

Both intramuscular and intrathecal interventions were given as soon as possible after hospital admission, with the intramuscular injection given first. To preserve masking and to comply with Vietnamese regulations for equine antitoxin use, participants received subcutaneous test doses of 0·5 mL of both human and equine preparations before the main intramuscular allocation. For the intrathecal intervention, the sham procedure consisted of screening the patient from view, positioning them in the lateral decubitus position and placing a dressing over the lumbar area, identical to that used in the intrathecal group. Intrathecal antitoxin was given by lumbar puncture, performed with patients in the lateral decubitus position after, if necessary, bolus doses of intravenous benzodiazepines and fentanyl. Injections were performed using a 20-gauge spinal needle via a 0·2 μm filter after removal of 2 mL of cerebrospinal fluid. Both groups of patients remained in the supine position for 4 h after the intervention and were monitored regularly, including careful neurological examination at 1 h. Due to safety concerns, intrathecal injections were generally not performed between 0000 h and 0600 h.

Routine management was delivered by ICU staff according to the management protocol of the Hospital for Tropical Diseases.^21^ Briefly, this consisted of antibiotics, wound care, and symptomatic control. Spasm control utilises benzodiazepines, escalating to non-depolarising neuromuscular blocking agents (pipecuronium), and mechanical ventilation. Autonomic nervous system disturbance was treated with magnesium sulphate as a first-line intervention, adding calcium antagonists, fentanyl, or inotropes as indicated. Airway management was by initial tracheostomy.^23^ According to hospital policy, patients received a first dose of a tetanus-toxoid-containing vaccine the day before hospital discharge with instructions about following doses and reminders at follow-up.

### Outcomes

The primary endpoint was requirement for mechanical ventilation during ICU stay. Criteria for mechanical ventilation were peripheral oxygen saturation (SpO_2_) less than 90%; or arterial oxygen partial pressure per fractional inspired oxygen (PaO_2_/FiO_2_) less than 250; or excessive spasms necessitating muscle paralysis.

Secondary endpoints were duration of ICU stay, duration of hospital stay, duration of mechanical ventilation, in-hospital and 240-day mortality, 240-day disability evaluated by the modified Rankin score, new antibiotic prescription during ICU stay (excluding antibiotics for tetanus or initial entry site infection), ventilator-associated pneumonia (VAP), syndrome of autonomic nervous system dysfunction (ANSD), daily maximum and minimum heart rate and systolic blood pressure, total dose and duration of benzodiazepines and pipecuronium during hospital stay, cost of hospital and ICU stay, and incidence of adverse events. ANSD was defined as at least three of: heart rate more than 100 beats per minute, systolic blood pressure more than 140 mm Hg, mean arterial pressure less than 60 mm Hg, pyrexia more than 38°C, and fluctuating blood pressure occurring within one day with no other apparent cause. VAP was diagnosed in patients receiving mechanical ventilation for at least 48 h and within the last 48 h, and at least two of: temperature of more than 38°C or less than 36°C, white blood cell count of less than 4·0 × 10^9^ cells per L or 12  × 10^9^ or more cells per L, purulent respiratory secretions, and new or progressive changes on chest radiography. Microbiologically confirmed VAP was defined as VAP plus bacterial growth of 1  × 10^5^ or more colony-forming units per mL from endotracheal aspirate. Outcomes were assessed by study staff blind to the study intervention at daily visits and 240-day outcomes by telephone (to patients and family members).

Active evaluation for adverse events was carried out daily. All adverse events regardless of grade or severity were recorded. Adverse events were defined according to the Common Terminology Criteria for Adverse Events (CTCAE), version 5, classed as any untoward medical event that a study participant experienced during the course of the study and followed the CTCAE grading (grade 1 mild to grade 4 severe). Serious adverse events were defined as those that were life-threatening or resulted in death, new inpatient hospitalisation or prolongation of existing hospitalisation, persistent or significant disability, or congenital anomaly. All serious adverse events and additional specified adverse events were reported to the study data monitoring and safety board and relevant ethical committees. Patients who were discharged home to die were considered in-hospital deaths and analysed as deaths occurring at the time of discharge. These deaths were confirmed by telephone follow-up.

The frequency of adverse events was summarised as the total number of adverse events and each adverse event separately. Tables were generated to summarise the proportion of patients with adverse events, tabulating adverse events by grade (1–4), number of adverse events, and whether these events were judged to be related or possibly related to the treatment intervention. Comparisons of the proportions were done with the χ^2^ test for independence; if the expected number was less than or equal to 1 in at least one of the cells, Fisher's exact test was used.

### Statistical analysis

All statistical analyses were carried out using R, version 4.0.2, according to a predefined statistical analysis plan (appendix p 89). Data were predominantly complete; thus, no corrections were made for missing data, and, for each analysis, numbers analysed are reported. The study sample size was calculated for the intrathecal intervention, assuming no significant interaction between interventions, resulting in a sample size of 250 patients calculated to detect an absolute risk reduction for mechanical ventilation due to intrathecal treatment by 17% (from 45% to 28%) with 80% power and two-sided 5% significance level. To allow for protocol violations and loss to follow-up, this was increased to 272 patients. Due to low mortality in our setting, mechanical ventilation was selected as the primary outcome and this difference was chosen as being the minimal clinically important effect.^19^

The main comparison of this factorial trial was the comparison between individuals receiving intrathecal human antitoxin versus those without (ie, sham procedure). As stated in the statistical analysis plan, similar evaluations were made for the intramuscular intervention (human intramuscular antitoxin versus equine intramuscular antitoxin). Logistic regression was used to compare the intervention groups in separate models, with sham procedure as the reference group for the intrathecal intervention and equine intramuscular antitoxin as the reference group for the intramuscular intervention. We additionally estimated relative risk between the groups based on a binary regression model with a loglink rather than the logit link function used in logistic regression. For both interventions, the intention-to-treat population consisted of all patients randomly assigned to that particular intervention. Previous intramuscular antitoxin administration resulted in fewer patients being randomly assigned to the intramuscular than intrathecal allocation. A secondary (exploratory) analysis of individuals receiving antitoxin at a previous hospital is included in the appendix. The time period between randomisation and the actual intervention resulted in a small number of participants meeting the primary endpoint before the allocated intervention (figure). As detailed in the statistical analysis plan, these were excluded from primary endpoint analyses, but were included in secondary outcome analyses, safety analyses, and a further sensitivity analyses of all patients (appendix p 81).

**Figure:**
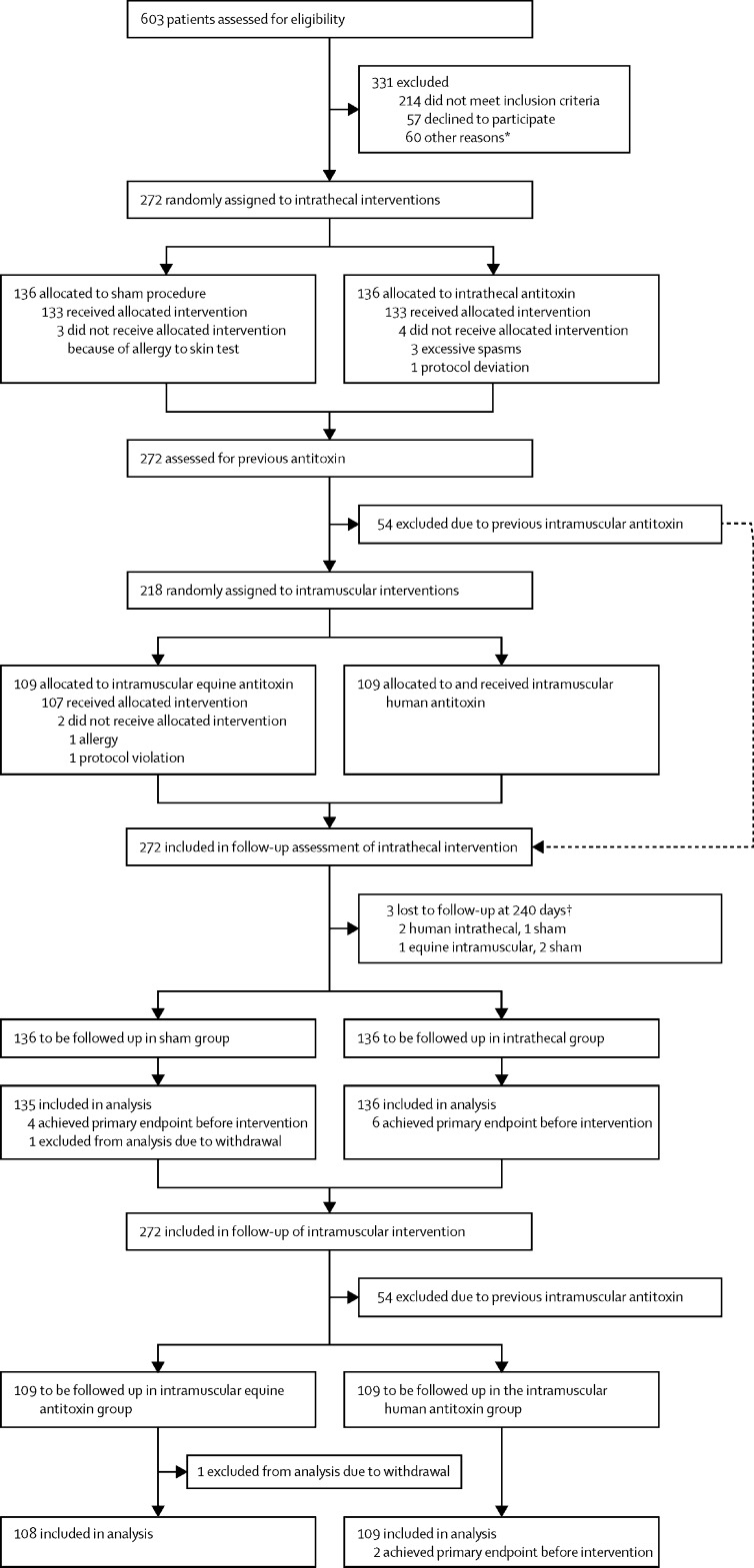
Trial profile *Insufficient intensive care unit beds available (n=19), no protocol drugs available (n=11), delayed diagnosis (n=6), unclear diagnosis (n=11), no available staff (n=3), and other (n=10). †Patients lost to follow up are included in the primary outcomes (in-hospital events), but not all secondary outcomes.

Interactions between the intrathecal and intramuscular interventions were assessed. Heterogeneity of the treatment effects in predefined groups of tetanus severity score (TSS), American Society of Anesthesiologist Score,^24^ age, and previous antitoxin administration was assessed for intramuscular and intrathecal interventions separately via interaction terms. The continuous variables TSS^25^ (calculated from information available before randomisation only) and age were included in the models using restricted cubic splines with three knots. Splines provide a flexible way to quantify the relation between a continuous variable and the outcome. The rcs package in R was used to model the spline regression. The knots were chosen at the 10th, 50th, and 90th percentile of the values of that variable as the default of the package. Robustness analyses with additional knots were also performed to test the heterogeneity of age. The knots were chosen at the 5%, 35%, 65%, and 95% for 4 knots; 5%, 27·5%, 50%, 72·5%, and 95% for 5 knots; and 5%, 23%, 41%, 59%, 77%, for 6 knots. Between each two successive knots, the relation was modelled via a cubic polynomial, while the relation was assumed to change smoothly on both sides of a knot.

For duration of hospital stay, ICU stay, and ventilation, in-hospital death was treated as a competing event. Cause-specific cumulative incidence was estimated and plotted and differences between groups tested using Gray's log-rank test. In-hospital mortality was assessed with a logistic regression model and 240-day mortality estimated via Kaplan-Meier curves and compared using log-rank test. Disability at 240 days was compared with a proportional odds logistic regression model. VAP, new antibiotic prescription during ICU stay, and ANSD were analysed using logistic regression. Daily maximum and minimum systolic blood pressure and heart rate during the first 7 days in hospital was compared using a linear mixed-effects model with days of measurement as a random linear and a random quadratic term, allowing for a quadratic trend over time for both fixed and random effect. Cumulative dose and duration of drugs and hospital costs were compared using linear regression. The total costs during ICU stay and the total costs during hospital stay were calculated as direct costs using itemised hospital bills. Costs in VND were converted to US$ by dividing by 22 660·83, as detailed in the statistical analysis plan (appendix p 89). Because these costs had a skewed distribution, the Box-Cox procedure with intervention group as covariate was used to find a suitable transformation (appendix p 9). After the transformation, groups were compared using linear regression. For the effect measure, the difference in expected values on the transformed scale is reported. Because this might be hard to interpret, the distribution of the cost variables by intervention group is plotted as a histogram, adding the mean value and 95% CI for each intervention group. The median of the costs on the original scale (US$) is also reported.

An independent data and safety monitoring review board oversaw the trial, reviewing all severe adverse events and performing interim analyses for safety endpoints at prespecified timepoints.

The trial is registered with ClinicalTrials.gov, NCT02999815; recruitment is completed.

### Role of the funding source

The funder had no role in study design, data collection, data analysis, data interpretation, or writing of the report.

## Results

272 patients were enrolled between Jan 8, 2017, and Sept 29, 2019, and followed up until May, 2020 (figure 1). In the intrathecal arm of the trial, 136 individuals were randomly assigned to sham procedure and 136 to antitoxin; in the intramuscular arm of the trial, 109 individuals were randomly assigned to equine antitoxin and 109 to human antitoxin. 54 patients received intramuscular antitoxin at a previous hospital and one patient randomly assigned to equine antitoxin and sham procedure withdrew from the study without giving consent for use of data and was excluded from all analyses. Three patients were lost to follow-up at 240 days. Baseline demographic and clinical data of enrolled patients (table 1; appendix p 3) showed balanced baseline characteristics in both intramuscular and intrathecal intervention groups. For individuals allocated to the intrathecal intervention, median time from ICU admission to receiving the intervention was 3·5 h (IQR 2·3–7·0). For individuals allocated to the intramuscular intervention, median time between ICU admission and the intervention was 2·0 h (1·5–2·6; appendix p 87).

**Table 1:** Baseline characteristics of intention-to-treat population

		**Intrathecal intervention**		**Intramuscular intervention**	
		Intrathecal treatment (n=136)	Sham procedure (n=135)	Equine antitoxin (n=108)	Human antitoxin (n=109)
Age, years		46·0 (38·0 to 57·0)	50·0 (41·0 to 60·0)	50·0 (40·8 to 61·0)	48·0 (39·0 to 59·0)
Sex					
	Female	22/136 (16%)	22/135 (16%)	22/108 (20%)	17/109 (16%)
	Male	114/136 (84%)	113/135 (84%)	86/108 (80%)	92/109 (84%)
Body-mass index, kg/m^2^		21·5 (19·9 to 23·4); n=136	20·9 (19·6 to 23·1)	21·6 (19·9 to 23·4)	20·9 (19·5 to 22·8)
Duration of illness, days		3·0 (2·0 to 5·0); n=136	3·0 (2·0 to 5·0)	3·0 (2·8 to 5·0)	3·0 (2·0 to 6·0)
Incubation period,*† days		8·0 (5·8 to 14·0); n=108	9·0 (6·0 to 14·0); n=101	8·0 (6·0 to 13·8); n=78	8·0 (5·0 to 12·0); n=83
Period of onset,*‡ hours		48·0 (24·0 to 72·0); n=117	48·0 (24·0 to 72·0); n=121	48·0 (24·0 to 72·0); n=92	48·0 (24·0 to 72·0); n=92
Ablett score*^26^					
	I	23/136 (16·9%)	24/135 (17·8%)	22/108 (20·4%)	21/109 (19·3%)
	II	100/136 (73·5%)	100/135 (74·1%)	79/108 (73·1%)	77/109 (70·6%)
	III	13/136 (9·6%)	11/135 (8·1%)	7/108 (6·5%)	11/109 (10·1%)
APACHE II score*^27^		4·0 (2·0 to 7·0)	4·0 (2·0 to 7·0); n=135	4·0 (2·0 to 8·0); n=107	4·0 (2·0 to 7·0)
SOFA score*^28^		0·0 (0·0 to 0·0)	0·0 (0·0 to 0·0); n=135	0·0 (0·0 to 0·0); n=107	0·0 (0·0 to 0·0)
Tetanus Severity Score*^25^		0·0 (−3·0 to 5·0)	0·0 (−3·0 to 4·0); n=135	0·0 (−3·0 to 4·0); n=107	0·0 (−3·0 to 5·0)

Data are median (IQR), n/N, or median IQR; n (where n differs from the column total). Ablett score=grade I, no spasms; grade II, tetanus with spasms not interfering with respiration; grade III, severe spasms interfering with respiration. APACHE II=Acute Physiology and Chronic Health Evaluation. SOFA=Sequential Organ Failure.

* Prognostic indicators on admission to hospital.

† Time from wound to first symptom.

‡ Time from first symptom to first spasm.

In the intramuscular intervention (intention-to-treat population), 48 (45%) of 107 patients allocated to human antitoxin received mechanical ventilation compared with 48 (44%) of 108 patients allocated to equine antitoxin (relative risk [RR] 1·01, 95% CI 0·75–1·36; p=0·95; table 2). For the intrathecal intervention (intention-to-treat population), 56 (43%) of 130 patients allocated to intrathecal antitoxin received mechanical ventilation compared with 65 (50%) of 131 patients allocated to sham procedures (RR 0·87, 95% CI 0·66–1·13; p=0·29) with an absolute risk reduction for mechanical ventilations due to intrathecal treatment by 7% (from 50% to 43%). The per-protocol population analysis for both comparisons showed similar results (table 2).

**Table 2:** Primary outcome, requirement for MV

		**No MV**	**MV**	RR (95% CI), p value*	**OR (95% CI), p value**
**Intention-to-treat population**					
Intrathecal, n=261					
	Sham procedure	66/131 (50%)	65/131 (50%)	..	..
	Intrathecal treatment	74/130 (57%)	56/130 (43%)	0·87 (0·66–1·13), 0·29	0·77 (0·47–1·25), 0·29
Intramuscular, n=215					
	Equine antitoxin	60/108 (56%)	48/108 (44%)	..	..
	Human antitoxin	59/107 (55%)	48/107 (45%)	1·01 (0·75–1·36), 0·95	1·02 (0·59–1·74), 0·95
**Per-protocol population**					
Intrathecal, n=254					
	Sham procedure	64/128 (50%)	64/128 (50%)	..	..
	Intrathecal treatment	73/126 (58%)	53/126 (42%)	0·84 (0·64–1·10), 0·21	0·73 (0·44–1·19), 0·21
Intramuscular, n=213					
	Equine antitoxin	59/106 (56%)	47/106 (44%)	..	..
	Human antitoxin	59/107 (55%)	48/107 (45%)	1·01 (0·75–1·37), 0·94	1·02 (0·59–1·75), 0·94

Data are n/n (%), unless stated otherwise. MV=mechanical ventilation. RR=relative risk. OR=odds ratio.

* Measured using values in the intervention row.

There was no evidence that the treatment effects of equine versus human intramuscular antitoxin varied in prespecified subgroups according to tetanus severity, physical status, or age (intention-to-treat and per-protocol populations; appendix pp 6–7). However, for the intrathecal intervention, we found some evidence of heterogeneity of treatment effect in the prespecified subgroups defined by age and pre-hospital antitoxin administration (appendix pp 6–9). In the intention-to-treat group, older participants given intrathecal antitoxin were less likely to be ventilated than those treated with sham procedure (appendix p 6), but this effect diminished in the per-protocol population and robustness analyses with additional knots (appendix p 8).

There was weak evidence that individuals who received equine intramuscular had a reduced requirement for mechanical ventilation in the intrathecal antitoxin group versus the sham group (intention-to-treat population RR 0·39, 95% CI 0·18–0·85; p=0·02; interaction term RR 2·30, 95% CI 0·76–7·06; p=0·14; appendix p 5).

In patients who had previously received antitoxin, 16 (76%) of 21 allocated to intrathecal antitoxin required mechanical ventilation compared with 16 (50%) of 32 allocated to sham procedure (odds ratio 3·20, 95% CI 0·99–11·7; p=0·06; appendix p 82), with evidence of an interaction between treatment with antitoxin at a previous hospital and intrathecal treatment (appendix p 7).

Secondary outcomes for intention-to-treat and per-protocol populations are shown in table 3, and in the appendix (pp 4, 16–60). For both intramuscular and intrathecal interventions, there was little evidence for any differences, although intrathecal antitoxin treatment was suggested to be associated with shorter ICU days (intention-to-treat population, median 13·5 days *vs* 16 days; table 3). However, the overall duration of hospital stays were similar across groups.

**Table 3:** Secondary outcomes (intention-to-treat population)

	**n**	**Summary measure**	**Effect measure**	**95% CI**	**p value**
Duration of ICU stay, days*					
Sham	135	16·0 (8·0–23·0)	..	..	..
Intrathecal	136	13·5 (8·0–21·0)	..	..	0·07
Equine intramuscular	108	14·0 (8·0–22·0)	..	..	..
Human intramuscular	109	13·0 (7·0–22·0)	..	..	0·68
Duration of hospital stay, days*					
Sham	135	24·0 (17·0–31·0)	..	..	..
Intrathecal	136	23·0 (18·0–29·3)	..	..	0·23
Equine intramuscular	108	23·0 (17·0–30·0)	..	..	..
Human intramuscular	109	23·0 (17·0–30·0)	..	..	0·85
Duration of mechanical ventilation, days*†					
Sham	69	17·0 (12·3–22·8)	..	..	..
Intrathecal	62	16·0 (11·0–21·0)	..	..	0·46
Equine intramuscular	48	17·0 (12·3–22·8)	..	..	..
Human intramuscular	50	16·0 (11·0–21·0)	..	..	0·71
**In-hospital deaths**‡					
Sham	135	4 (3%)	..	..	..
Intrathecal	136	3 (2%)	OR 0·74	0·14 to 3·41	0·70
Equine intramuscular	108	4 (4%)	..	..	..
Human intramuscular	109	2 (2%)	OR 0·49	0·07 to 2·54	0·41
**240-day deaths**§					
Sham	134	6 (4%)	..	..	..
Intrathecal	134	5 (4%)	OR 0·82	0·23 to 2·79	0·75
Equine intramuscular	107	6 (6%)	..	..	..
Human intramuscular	107	4 (4%)	OR 0·65	0·16 to 2·33	0·51
**240-day Rankin score,**¶**severe (score >2)**					
Sham	134	18 (13%)	..	..	..
Intrathecal	134	11 (8%)	OR 0·58	0·25 to 1·26	0·17
Equine intramuscular	107	14 (13%)	..	..	..
Human intramuscular	107	9 (8%)	OR 0·61	0·24 to 1·46	0·28
**Ventilator-associated pneumonia**†‡					
Sham	69	30 (43%)	..	..	..
Intrathecal	62	26 (42%)	OR 0·94	0·47 to 1·88	0·86
Equine intramuscular	48	20 (42%)	..	..	..
Human intramuscular	50	25 (50%)	OR 1·40	0·63 to 3·13	0·41
**Microbiologically confirmed ventilator-associated pneumonia**‡					
Sham	69	24 (35%)	..	..	..
Intrathecal	62	21 (34%)	OR 0·96	0·46 to 1·98	0·91
Equine intramuscular	48	16 (33%)	..	..	..
Human intramuscular	50	21 (42%)	OR 1·45	0·64 to 3·33	0·38
**New antibiotics during hospitalisation**‡					
Sham	135	61 (45%)	..	..	..
Intrathecal	136	51 (38%)	OR 0·73	0·45 to 1·18	0·20
Equine intramuscular	108	40 (37%)	..	..	..
Human intramuscular	109	44 (40%)	OR 1·15	0·67 to 1·99	0·61
**Autonomic nervous system dysfunction**‡					
Sham	135	28 (21%)	..	..	..
Intrathecal	136	28 (21%)	OR 0·99	0·55 to 1·79	0·98
Equine intramuscular	108	22 (20%)	..	..	..
Human intramuscular	109	21 (19%)	OR 0·93	0·48 to 1·82	0·84
**Total dose pipecuronium, mg**‖					
Sham	69	386 (162–646)	..	..	..
Intrathecal	62	452 (222–637)	Beta 1·5	−1·6 to 4·6	0·343
Equine intramuscular	48	376 (147–624)	..	..	..
Human intramuscular	50	444 (114–713)	Beta 0·3	−3·5 to 4·1	0·877
**Duration of pipecuronium, days**‖					
Sham	69	11·0 (6·0–17·0)	..	..	..
Intrathecal	62	13·5 (8·0–17·0)	Beta 1·0	−1·4 to 3·4	0·419
Equine intramuscular	48	11·0 (5·8–17·0)	..	..	..
Human intramuscular	50	11·5 (5·3–18·0)	Beta 0·5	−2·5 to 3·5	0·734
**Total benzodiazepines, mg**‖					
Sham	135	4718 (1686–9224)	..	..	..
Intrathecal	136	4817 (1858–8327)	Beta −0·5	−7·7 to 6·8	0·902
Equine intramuscular	108	3457 (1438–7890)	..	..	..
Human intramuscular	109	5588 (1686–9021)	Beta 6·2	−2·0 to 14·4	0·139
**Duration of benzodiazepines, days**‖					
Sham	135	1·40 (1·26–1·51)	..	..	..
Intrathecal	136	1·38 (1·28–1·48)	Beta −0·02	−0·1 to 0·03	0·392
Equine intramuscular	108	1·38 (1·26–1·49)	..	..	..
Human intramuscular	109	1·38 (1·26–1·49)	Beta −0·004	−0·063 to 0·055	0·884
**ICU cost, US$**‖**					
Sham	135	1150 (259–2632)	..	..	..
Intrathecal	136	735 (276–2316)	Beta −0·1	−0·2 to 0·1	0·258
Equine intramuscular	108	721 (232–2526)	..	..	..
Human intramuscular	109	744 (271–2656)	Beta 0·02	−0·13 to 0·17	0·825
**Hospital cost, US$**‖**					
Sham	135	1217 (369–2726)	..	..	..
Intrathecal	136	861 (391–2423)	Beta −0·07	−0·18 to 0·05	0·25
Equine intramuscular	108	791 (349–2599)	..	..	..
Human intramuscular	109	843 (351–2737)	Beta 0·01	−0·12 to 0·14	0·908

Data are median (IQR), or n (%), unless stated otherwise. ICU=intensive care unit. OR=odds ratio. Based on the Box-Cox diagnoses, beta coefficients of groups were compared on a log transformation scale for ICU costs and hospital costs, and on a square root transformation scale for dose of pipecuronium and benzodiazepines to reduce non-normality of the errors in linear regression models.

* p value relates to cause-specific cumulative incidence tested using Gray's log-rank test.

† Ventilated patients only.

‡ p value is based on logistic regression model.

§ p value relates to comparison of survival curves using log-rank test.

¶ p value relates to proportional odds logistic regression model.

‖ p value is based on linear regression model.

** Costs exclude those of antitoxin and any lumbar puncture procedure.

The majority of adverse events were mild, and approximately 40% of patients had no adverse events (table 4). When evaluated according to interventions, there was no difference in either the number of patients with adverse events or the severity of events occurring in individuals in either the intrathecal group compared with sham or the intramuscular interventions (table 4). Events judged to be related to or possibly related to the intervention are shown in table 4 and the appendix (pp 63–66). Overall, 22 (16%) of 136 individuals allocated to the intrathecal group and 15 (11%) of 135 individuals allocated to the sham procedure had adverse events judged to be related or possibly related to the intervention, with only four in each group graded as grade 3 or 4 events. For the intramuscular intervention, 16 (15%) of 108 allocated to equine antitoxin and 17 (16%) of 109 allocated to human antitoxin had adverse events related or possibly related to the intervention. 13 severe adverse events occurred in patients allocated to the sham group and eight in those allocated to the intrathecal group. 12 severe adverse events occurred in patients allocated to the equine group and nine occurred in the human intramuscular group. There were no deaths judged to be related to the study interventions.

**Table 4:** Number and characteristics of adverse events by intervention

		**Intrathecal intervention**			**Intramuscular intervention**		
		Intrathecal antitoxin	Sham procedure	**p value**	Equine antitoxin	Human antitoxin	**p value**
Related adverse events		2/136 (1%)	2/135 (1%)	0·994	2/108 (2%)	2/109 (2%)	0·993
Possibly related adverse events		20/136 (15%)	13/135 (10%)	0·201	14/108 (13%)	15/109 (14%)	0·863
Unrelated adverse events		72/136 (53%)	84/135 (62%)	0·122	61/108 (56%)	58/109 (53%)	0·628
Number of adverse events per patient		..	..	0·076	..	..	0·779
	0	52/136 (38%)	47/135 (35%)	..	41/108 (38%)	41/109 (38%)	..
	1	27/136 (20%)	27/135 (20%)	..	18/108 (17%)	24/109 (22%)	..
	2	17/136 (12%)	21/135 (16%)	..	16/108 (15%)	17/109 (16%)	..
	3	6/136 (4%)	13/135 (10%)	..	10/108 (9%)	5/109 (5%)	..
	4	14/136 (10%)	9/135 (7%)	..	8/108 (7%)	10/109 (9%)	..
	5	13/136 (10%)	4/135 (3%)	..	6/108 (6%)	4/109 (4%)	..
	>5	7/136 (5%)	14/135 (10%)	..	9/108 (8%)	8/109 (7%)	..

Data are n/N (%), unless stated otherwise.

## Discussion

We found no evidence that additional intrathecal antitoxin reduced requirement for mechanical ventilation in adults already treated with intramuscular antitoxin, or substantively improved any of the secondary outcomes. Treatment with human intramuscular antitoxin did not reduce mechanical ventilation requirements in adults with tetanus, or improve any secondary outcomes, compared with standard equine intramuscular antitoxin treatment.

Our study was done in an ICU that specialises in the treatment of tetanus (around 300 cases per year) and it is possible that the limited observed treatment effects of intrathecal administration, in particular, might be different in settings with less expertise and resources. Of the 12 studies included in a 2006 meta-analysis of intrathecal antitoxin,^29^ median mortality in controls was 55%, reflecting limited access to mechanical ventilation (unlike in our setting), and many previous studies were subject to significant methodological bias. The only data from a setting similar to ours came from a Brazilian ICU, which showed that intrathecal administration was associated with reduced disease progression. Mechanical ventilation was reduced in the intrathecal group (38% in individuals treated with 1000 IU intrathecal human antitoxin compared with 55% in controls), but the difference did not meet statistical significance.^16^ It is therefore possible that there is a smaller benefit from intrathecal intervention than our study was powered to detect. Our sample size was calculated after a consensus among international and local physicians regarding the degree of mechanical ventilation reduction that would constitute an appropriate clinical benefit, applying knowledge of increased cost and nosocomial infections in patients with tetanus receiving mechanical ventilation.^19^ Of note, we saw no differences in the related secondary outcomes of VAP, duration of ventilation, or antibiotic use during admission, which constituted much of the rationale for reducing mechanical ventilation rates overall.

Other authors have reported reduced disease progression as endpoints in the evaluation of intrathecal antitoxin; thus, it might be expected that a clinically meaningful reduction in mechanical ventilation would be linked to an overall less severe spectrum of disease. However, we observed no indication of benefit of intrathecal antitoxin in secondary endpoints of endpoints of ANSD, doses, and duration of muscle relaxants. The underlying explanation for the reduction in ICU length of stay observed in the per-protocol group remains unclear in view of the unchanged outcomes related to ventilation, disease severity, or hospital length of stay.

It is possible that an insufficient dose of intrathecal antitoxin is an explanation for our findings, and insufficient knowledge of CNS antitoxin pharmacokinetics is a limitation of our study. Our choice was based on previous studies using doses of 250–1500 IU without any observable dose–response effect, and animal data showing that substantially lower doses of antitoxin are effective if administered intrathecally compared with other routes.^9^, ^29^, ^30^

No differences in mechanical ventilation requirements were seen between treatment allocations in any of the predefined subgroups, except individuals treated with intramuscular antitoxin before admission, for whom there was an increased requirement for mechanical ventilation in those treated with intrathecal antitoxin. Reasons for a difference in this group are unclear, and might include greater disease severity on admission, but it might also be a chance finding. It is possible that in patients with rapidly progressing disease, the stimulatory procedure of lumbar puncture provoked further spasms, necessitating ventilation. Insufficiency of data detailing times of antitoxin in previous hospitals meant that we are unable to investigate this further. With respect to interactions between the two interventions, our results support our assumption and historical observations of limited interaction between intrathecal and intramuscular treatments.^12^ Although we observed a reduced ventilation rate in individuals treated with intrathecal antitoxin and equine intramuscular antitoxin in our hospital, we interpret this with caution, given the contradictory observation in individuals previously treated with equine antitoxin and the small numbers.

An important finding of our study was the safety of both interventions. There was no evidence of worse clinical outcomes 240 days after enrolment after intrathecal antitoxin, or intramuscular preparation, and adverse event frequency was similar. Only minor expected events, such as headache, were apparent in the intrathecal group.

A limitation of our study was that we did not include formal health economic analyses, nor antitoxin costs in hospital cost analysis, because human antitoxin was not commercially available in Vietnam at the time of our study. For LMICs, where cost of ICU care is less than in high-income countries, the approximately 10-times reduction in antitoxin costs associated with equine antitoxin are an important consideration in determining treatment. Elsewhere, savings associated with shorter ICU stay related to intrathecal treatment might be more relevant. A cost-effectiveness analysis from the Brazilian trial suggested significant health economic benefits to the use of intrathecal antitoxin.^31^

Our study was done in a single centre, which might limit generalisability. The complexity of delivering separate blinded interventions necessitated our use of a site with both sufficient numbers of cases and experience in high-quality clinical trial conduct. Mortality rates in this study are similar to those at our centre over recent years.^23^ Reasons for low mortality in our centre are discussed in more detail elsewhere; however, we believe a standard treatment protocol and staff experience are important.^23^ We were unable to contact three patients after discharge, potentially missing post-discharge deaths or other complications. However, given the small number involved, this is unlikely to have had a significant effect on our results. In other ways, the population recruited to our study was similar in structure and severity to contemporary series from other LMICs. The age and sex of cases probably reflects the focus of tetanus vaccination programmes in Vietnam and other LMICs, which are directed towards infants and pregnant women.^18^, ^32^ Increasing vaccination coverage and improving the quality of ICU care remain critical strategies in improving outcomes from tetanus in LMICs.

In summary, we found that additional administration of intrathecal antitoxin, although safe, does not add overall benefit in the treatment of tetanus, particularly in settings similar to Vietnam with good access to mechanical ventilation and low mortality. Our findings indicate human intramuscular antitoxin did not offer any benefit for tetanus treatment compared with equine intramuscular antitoxin. The study highlights the importance of achieving high coverage in global tetanus vaccination programmes.

## Data sharing

Data collected for the study, including de-identified participant data and a data dictionary defining each field in the set will be made available to others with publication at the Oxford Research Archive (https://ora.ox.ac.uk/) under the terms of the Creative Commons Attribution 4·0 International license (CC-BY 4·0). The study protocol can be accessed at https://wellcomeopenresearch.org/articles/3–58. The statistical analysis plan is included in the appendix.

## Declaration of interests

All authors declare no competing interests.
