# Supplementary material for: Human versus equine intramuscular antitoxin, with or without human intrathecal antitoxin, for the treatment of adults with tetanus: a 2 × 2 factorial randomised controlled trial
Source: Lancet Glob Health. 2022 May 10;10(6):e862–72. doi: 10.1016/S2214-109X(22)00117-6 (PMC9115864; doi:10.1016/S2214-109X(22)00117-6)
Supplement: Supplementary appendix [file mmc1.pdf]

THE LANCET
Global Health

Supplementary appendix

This appendix formed part of the original submission and has been peer reviewed. We post it as supplied by the authors.

Supplement to: Van Hao N, Thi Loan H, Minh Yen L, et al. Human versus equine intramuscular antitoxin, with or without human intrathecal antitoxin, for the treatment of adults with tetanus: a 2 × 2 factorial randomised controlled trial. *Lancet Glob Health* 2022; **10:** e862–72.

Supplementary Appendix

Contents

Supplementary Tables and Figures 3

Table S1 Baseline characteristics of study populations (per-protocol population) 3

Table S2 Baseline characteristics of patients treated with intramuscular antitoxin, including those treated at a previous hospital 4

Table S3 Interactions between intramuscular and intrathecal interventions intention-to-treat populations 5

Figure S1 Pre-specified subgroup analysis for intrathecal and intramuscular interventions (intention-to-treat populations): tetanus severity and age 6

Figure S2: Pre-specified subgroup analysis for intrathecal and intramuscular interventions (intention-to-treat populations): ASA score and antitoxin at previous hospital 7

Figure S3 Assessment of heterogeneity of the treatment effect and age with variable knots 8

Figures S4&5 Pre-specified subgroup analysis of heterogeneity of treatment effect in per-protocol population 9

Figure S6 Heterogeneity of treatment effect in pre-specified subgroups including population receiving antitoxin at previous hospital. 11

Table S4 Secondary outcomes in per-protocol populations 12

Figure S7 Secondary outcomes – cardiovascular parameters: intrathecal and intramuscular (IM) interventions (intention-to-treat populations) 16

Figure S8 Cardiovascular parameters for the intramuscular (IM) intervention including population receiving antitoxin at previous hospital. 18

Figures S9a-c Duration of intensive care unit (ICU) stay 19

Figures S10a-c: Duration of hospital stay 22

Figures S11a-c Time from treatment to start mechanical ventilation 25

Figures S12a-c Duration of mechanical ventilation 28

Table S5: in-hospital mortality including population receiving antitoxin at previous hospital 31

Table S6: 240-day mortality including population receiving antitoxin at previous hospital 31

Figures S13 a-c Kaplan Meier Curves for 240-day mortality 32

Tables S7 a-f: 240-day disability 35

Tables S8a&b Ventilator associated pneumonia (VAP) in ventilated patients, intramuscular intervention including population receiving antitoxin at previous hospital 37

Table S9 Clinical syndrome of autonomic nervous system dysfunction (ANSD) intramuscular intervention including population receiving antitoxin at previous hospital 38

Table S10 New antibiotic prescription during ICU stay intramuscular intervention including population receiving antitoxin at previous hospital 38

Figures S14a-c Total dose of pipecuronium during hospital stay (for patients ventilated)..........39
Figures S15 a-c Total duration of pipecuronium (for patients ventilated) 42
Figures S16a-c Total dose of Diazepam during hospital stay...45
Figures S17 a-c Total dose of midazolam during hospital stay 48
Figures S18a-c Total dose of benzodiazepines during hospital stay...51
Figures S19a-c Total duration of benzodiazepines 54
Figures S20a-c Cost of ICU Stay..57
Figures S21a-c Cost of hospital Stay 60
Table S11 Frequency of adverse events classified as 'possibly related' or 'related to' interventions (intention –to-treat population)...63
Table S12 Number of patients with adverse events (AE) (Intramuscular intervention per-protocol population) 64
Table S13 Number of patients with adverse events(AE) (Intrathecal intervention per-protocol population)..65
Table S14 Number of patients with adverse events (AE) (Intramuscular intervention including population receiving antitoxin at previous hospital) 66
Table S15 All adverse events (Intramuscular intervention intention-to-treat population)..........67
Table S16 All adverse events (Intramuscular intervention per-protocol population) 70
Table S17 All adverse events (Intrathecal intervention intention-to-treat population) 73
Table S18 All adverse events (Intrathecal intervention per-protocol population).......................75
Table S19 All adverse events (Intramuscular intervention including pre-hospital intramuscular antitoxin population) 78
Tables S20a-c Primary outcome: requirement for mechanical ventilation: without exclusions for early mechanical ventilation 81
Table S21 Interaction between treatments including intramuscular antitoxin at previous hospital population (intention-to-treat populations)...82
Table S22 Baseline characteristics of patients treated at previous hospital antitoxin and with intrathecal treatment only..83
Table S23 a&b Time from ICU admission to intervention: intention-to-treat and per-protocol populations 85
Figure S22 Histogram showing numbers of males and females in whole study population........86
Figure S23 Flow diagram showing intrathecal population only..87
Figure S24 Flow diagram showing intramuscular population only..88
Statistical Analysis Plan 89
References 104

Supplementary Tables and Figures

Table S1 Baseline characteristics of study populations (per-protocol population)

	Intrathecal intervention				Intramuscular intervention			
	Intrathecal antitoxin (N=132)		Sham procedure (N=132)		Equine antitoxin (N=106)		Human antitoxin (N=109)	
**Characteristic**	N	Median (IQR), or count (%)	N	Median (IQR), or count (%)	n	Median (IQR), or count (%)	n	Median (IQR), or count (%)
Age [years]	132	46.0 (38.0, 57.5)	132	51.5 (41.0, 60.0)	106	50.0 (41.0, 61.0)	109	48.0 (39.0, 59.0)
Female Sex	132	22/132 (16.7%)	132	22/132 (16.7%)	106	22/106 (20.8%)	109	17/109 (15.6%)
Body mass index [kg/m2]	132	21.4 (19.9, 23.2)	132	20.9 (19.5, 23.1)	106	21.6 (19.9, 23.4)	109	20.9 (19.5, 22.8)
Duration of illness [days]	132	3.0 (2.0, 5.0)	132	3.0 (2.0, 5.0)	106	3.0 (2.2, 5.0)	109	3.0 (2.0, 6.0)
Incubation period [days]*	104	8.0 (5.0, 13.2)	100	9.0 (6.0, 14.0)	77	8.0 (6.0, 13.0)	83	8.0 (5.0, 12.0)
Period of onset [hours]*	113	48.0 (24.0, 72.0)	118	48.0 (24.0, 72.0)	90	48.0 (24.0, 72.0)	92	48.0 (24.0, 72.0)
Ablett Score on admission*	132		132		106		109	
- I		72/132 (54.5%)		72/132 (54.5%)		22/106 (20.8%)		21/109 (19.3%)
- II		54/132 (40.9%)		48/132 (36.4%)		77/106 (72.6%)		77/109 (70.6%)
- III		6/132 (4.5%)		12/132 (9.1%)		7/106 (6.6%)		11/109 (10.1%)
APACHE II score*	131	4.0 (2.0, 7.0)	132	4.0 (2.0, 7.0)	105	4.0 (2.0, 8.0)	109	4.0 (2.0, 7.0)
SOFA score*	131	0.0 (0.0, 0.0)	132	0.0 (0.0, 0.0)	105	0.0 (0.0, 0.0)	109	0.0 (0.0, 0.0)
Tetanus Severity Score*	131	0.0 (-3.0, 4.0)	132	0.0 (-3.0, 4.0)	105	0.0 (-3.0, 4.0)	109	2.0 (-3.0, 5.0)

* Prognostic indicators on admission to hospital. Incubation period is the period from wound to first symptom; period of onset is the period from first symptom to first spasm; Ablett score: Grade I: no spasms; II tetanus with spasms not interfering with respiration; III severe spasms interfering with respiration (3); APACHE II (4), Sequential Organ Failure Score (5), Tetanus Severity Score (2)

Table S2 Baseline characteristics of patients treated with intramuscular antitoxin, including those treated at a previous hospital

	Equine IM (N=108)		Antitoxin in previous hospital[†] (N=54)		Human IM (N=109)	
**Characteristic**	n	Median (IQR), or count (%)	n	Median (IQR), or count (%)	n	Median (IQR), or count (%)
Age [years]	108	50.0 (40.8, 61.0)	54	48.5 (39.2, 56.0)	109	48.0 (39.0, 59.0)
Female Sexh	108	22/108 (20.4%)	54	5/54 (9.3%)	109	17/109 (15.6%)
BMI [kg/m2]	108	21.6 (19.9, 23.4)	54	21.5 (20.2, 23.5)	109	20.9 (19.5, 22.8)
Duration of illness [days]	108	3.0 (2.8, 5.0)	54	3.0 (2.0, 4.0)	109	3.0 (2.0, 6.0)
Incubation period [days]*	78	8.0 (6.0, 13.8)	48	10.0 (6.0, 14.0)	83	8.0 (5.0, 12.0)
Period of onset [hours]*	92	48.0 (24.0, 72.0)	54	48.0 (24.0, 87.0)	92	48.0 (24.0, 72.0)
Ablett Score on admission*	108		54		109	
- I		22/108 (20.4%)		4/54 (7.4%)		21/109 (19.3%)
- II		79/108 (73.1%)		44/54 (81.5%)		77/109 (70.6%)
- III		7/108 (6.5%)		6/54 (11.1%)		11/109 (10.1%)
APACHE II score*	107	4.0 (2.0, 8.0)	54	3.0 (1.0, 6.0)	109	4.0 (2.0, 7.0)
SOFA score*	107	0.0 (0.0, 0.0)	54	0.0 (0.0, 0.0)	109	0.0 (0.0, 0.0)
Tetanus Severity Score*	107	0.0 (-3.0, 4.0)	54	0.0 (-3.0, 3.8)	109	2.0 (-3.0, 5.0)

* Prognostic indicators on admission to hospital. Incubation period is the period from wound to first symptom; period of onset is the period from first symptom to first spasm; Ablett score: Grade I: no spasms; II tetanus with spasms not interfering with respiration; III severe spasms interfering with respiration (3); APACHE II (4), Sequential Organ Failure Score (5), Tetanus Severity Score (2).

[†] Antitoxin at previous hospital was intramuscular equine origin only.

Table S3 Interactions between intramuscular and intrathecal interventions intention-to-treat populations

	Variable	No MV[1]	MV	RR[2] for MV	95% CI[3]	P-value
In Sham procedure group	**N = 99**					
	Equine intramuscular antitoxin	22 (44%)	28 (56%)	—	—	
	Human intramuscular antitoxin	28 (57%)	21 (43%)	0.59	0.26, 1.30	0.19
In intrathecal treatment group	**N = 109**					
	Equine intramuscular antitoxin	38 (67%)	19 (33%)	—	—	
	Human intramuscular antitoxin	31 (60%)	21 (40%)	1.35	0.62, 2.98	0.45
In Human intramuscular antitoxin group	**N = 101**					
	Sham procedure	28 (57%)	21 (43%)	—	—	
	Intrathecal treatment	31 (60%)	21 (40%)	0.90	0.41, 2.00	0.80
In Equine intramuscular antitoxin group	**N = 107**					
	Sham procedure	22 (44%)	28 (56%)	—	—	
	Intrathecal treatment	38 (67%)	19 (33%)	0.39	0.18, 0.85	0.02
Overall interaction	Human intramuscular antitoxin* Intrathecal treatment			2.30	0.76, 7.06	0.14

[1]Mechanical Ventilation, [2]RR = Relative Risk; [3]CI = Confidence Interval. As detailed in statistical analysis plan, analysis excludes those requiring mechanical ventilation before receiving intrathecal and intramuscular treatments in hospital

Figure S1 Pre-specified subgroup analysis for intrathecal and intramuscular interventions (intention-to-treat populations): tetanus severity and age

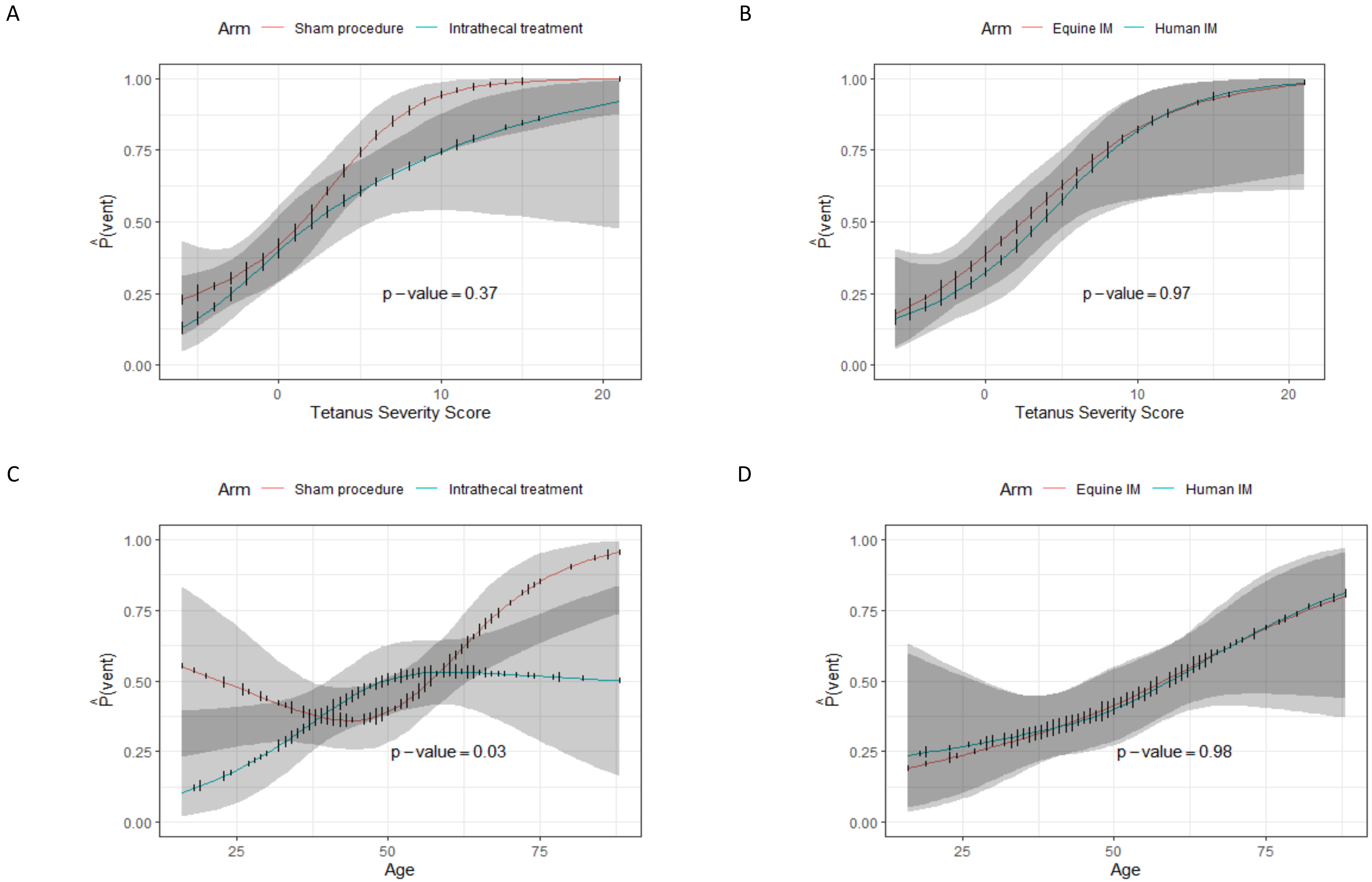

Figure showing heterogeneity of treatment effect in pre-specified subgroups: Tetanus Severity score (2) and intrathecal intervention (A); Tetanus Severity score and intramuscular (IM) intervention (B); age and intrathecal intervention (C) and age and intramuscular intervention (D). P(vent) Probability for requiring mechanical ventilation. P values calculated from restricted spline regression with knots as detailed in statistical methods section.

Figure S2: Pre-specified subgroup analysis for intrathecal and intramuscular interventions (intention-to-treat populations): ASA score and antitoxin at previous hospital

A

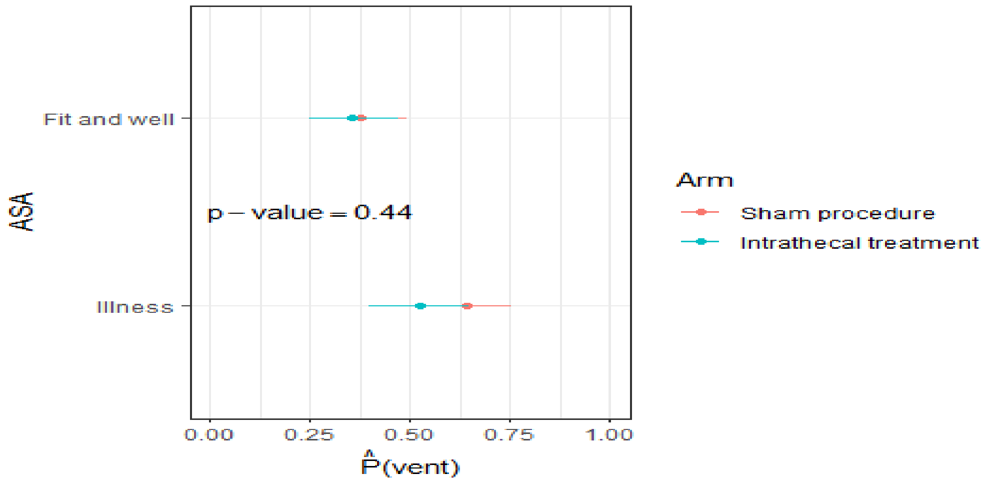

B

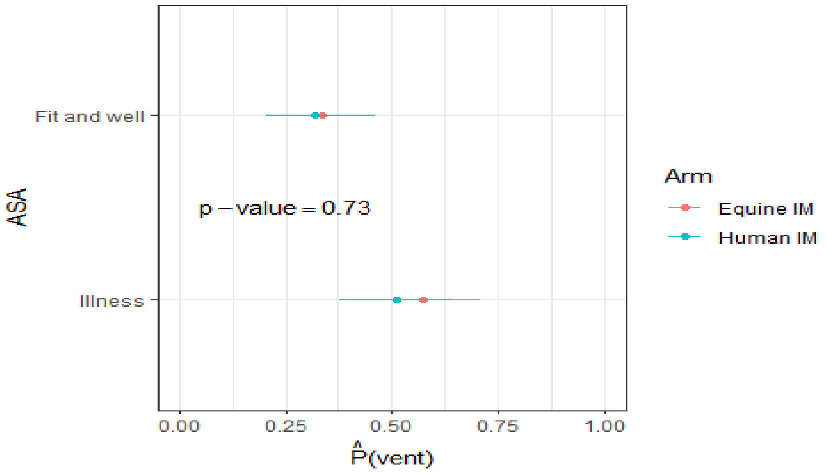

C

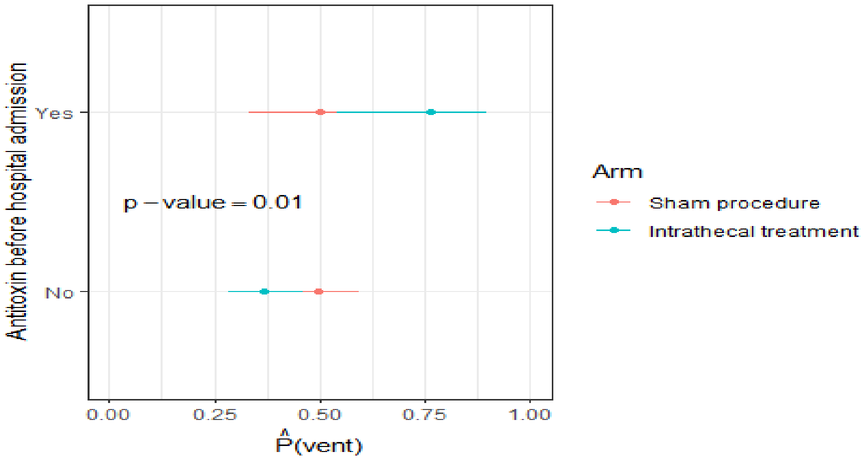

Figure showing heterogeneity of pre-specified subgroups American Society of Anestheologists (ASA) score (6) and treatment with antitoxin at previous hospital: intrathecal intervention and ASA score (A) ; intramuscular (IM) intervention and ASA score (B) and intrathecal intervention and antitoxin at previous hospital (note antitoxin available at previous hospitals was intramuscular equine origin only) (C)

Figure S3 Assessment of heterogeneity of the treatment effect and age with variable knots

Figure S3 Heterogeneity of the treatment effect by age in intention-to-treat population

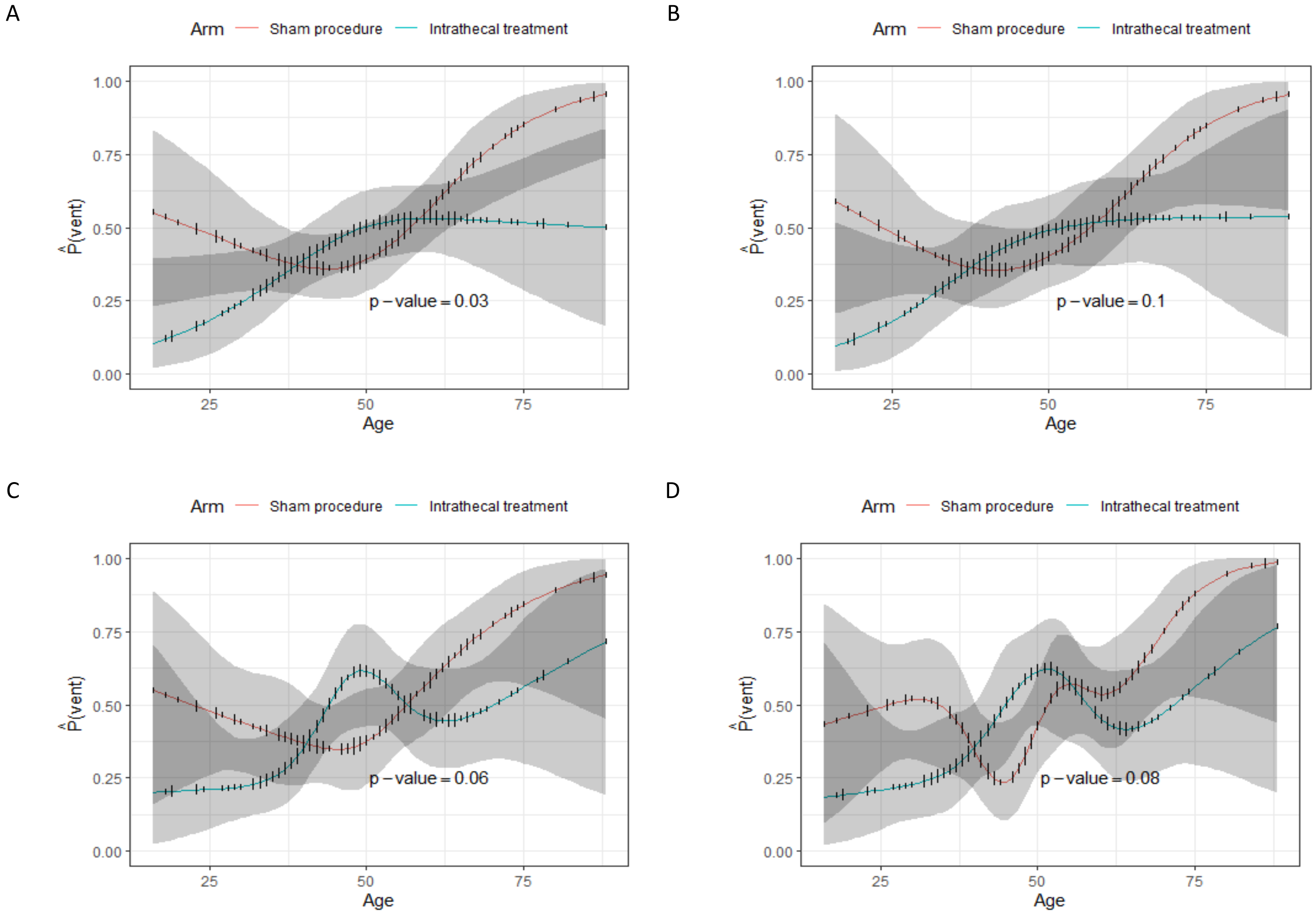

Figure showing heterogeneity of treatment effect in age with different number of knots in intention-to-treat population: age and intrathecal intervention (3 knots) (A); age and intrathecal intervention (4 knots) (B); age and intrathecal intervention (5 knots) (C) and age and intrathecal intervention (6 knots) (D). P(vent) probability of requiring mechanical ventilation. P values calculated from restricted cubic spline regression with knots as detailed in statistical methods section.

Figures S4&5 Pre-specified subgroup analysis of heterogeneity of treatment effect in per-protocol population

Figure S4 Heterogeneity of the treatment effect in per-protocol population by tetanus severity and age

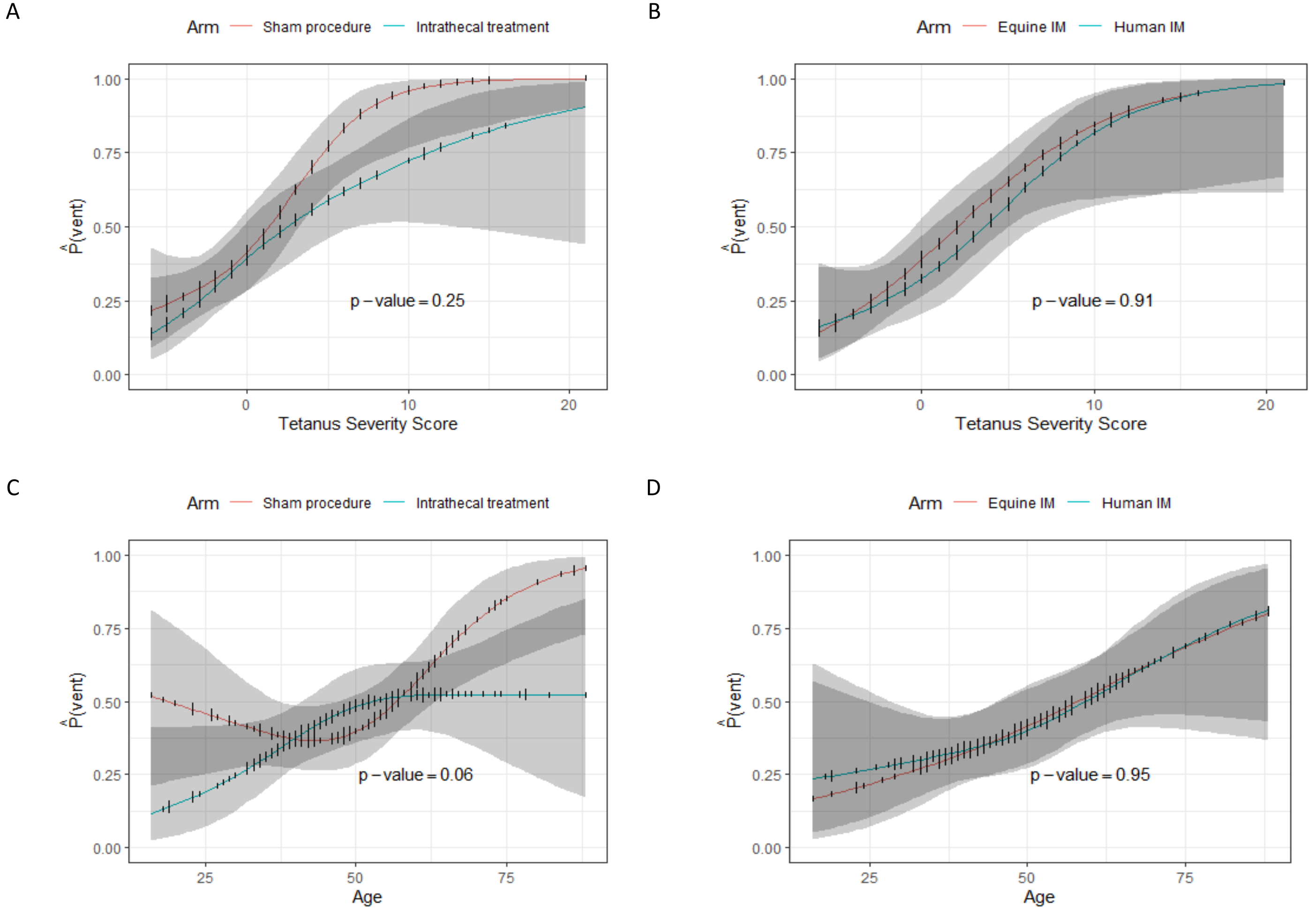

Figure showing heterogeneity of treatment effect in pre-specified subgroups: Tetanus Severity score (2) and intrathecal antitoxin (A); Tetanus Severity score and intramuscular (IM) intervention (B); age and intrathecal intervention (C) and age and intramuscular (IM) intervention (D). P(vent) probability of requiring mechanical ventilation. P values calculated from restricted cubic spline regression with knots as detailed in statistical methods section.

Figure S5 Heterogeneity of the treatment effect in per-protocol population by ASA physical state scale and antitoxin treatment at previous hospital

A

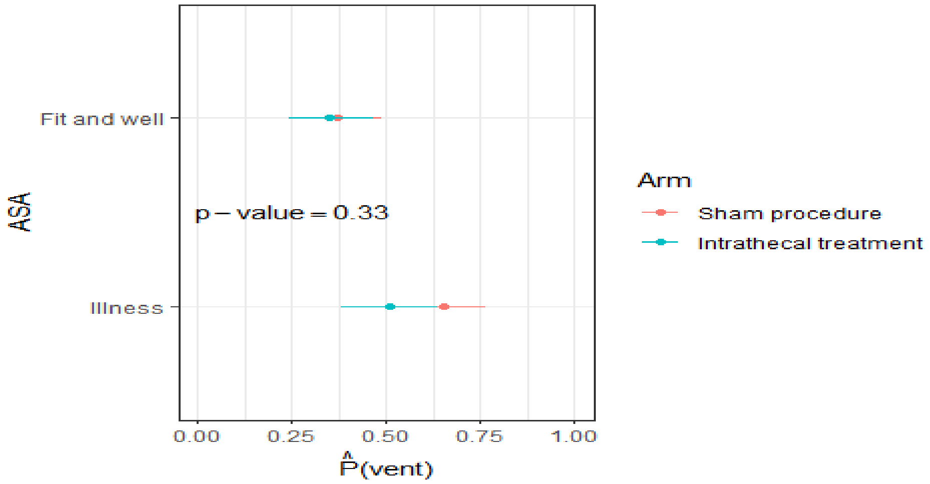

B

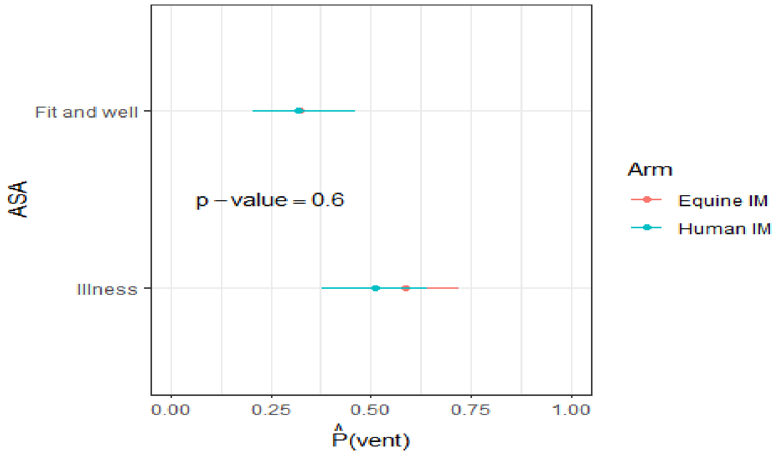

C

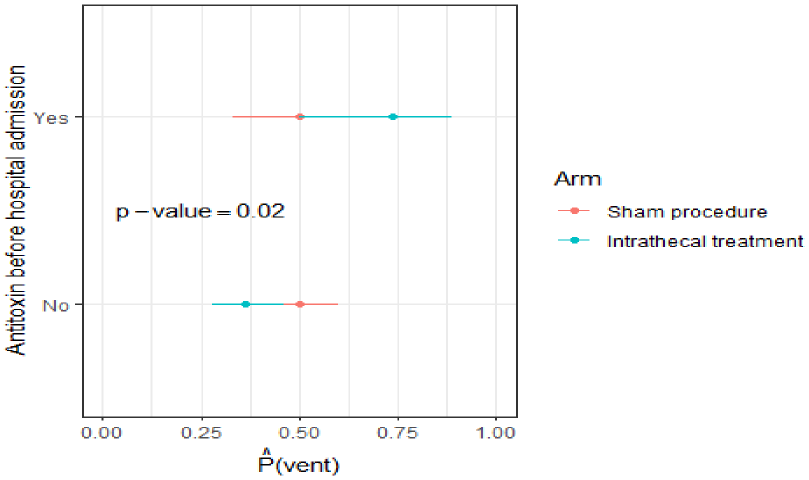

Figure showing heterogeneity of pre-specified subgroups ASA score (6) and treatment with antitoxin at previous hospital: intervention and ASA score (A) ; intramuscular (IM) intrathecal intervention and ASA score (B) and intrathecal intervention and previous antitoxin (C) P(vent) probability of requiring mechanical ventilation. Antitoxin available at previous hospital was intramuscular equine origin only.

Figure S6 Heterogeneity of treatment effect in pre-specified subgroups including population receiving antitoxin at previous hospital.

A

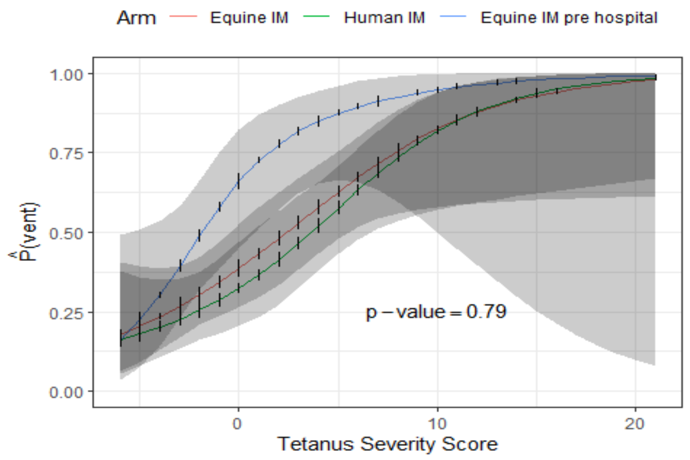

B

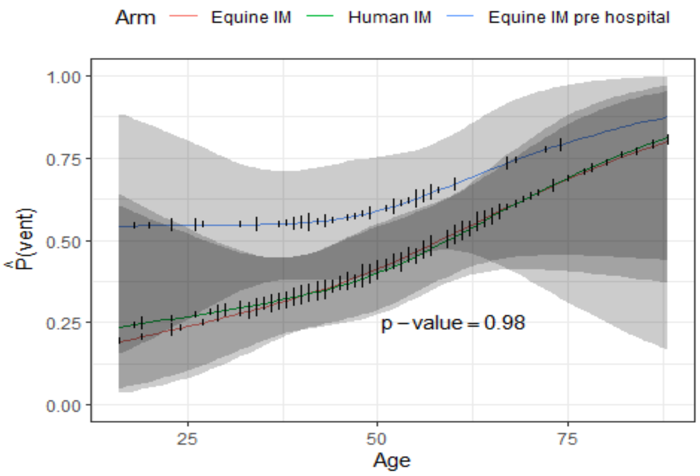

C

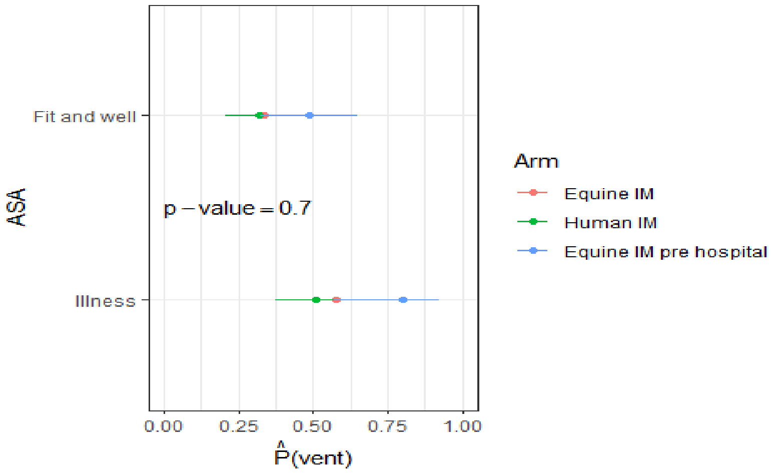

Figure showing heterogeneity of pre-specified subgroups: Tetanus Severity score and intramuscular (IM) antitoxin including pre-hospital treatment group (A); Age and intramuscular intervention including pre-hospital treatment group (B); intramuscular intervention and ASA score including pre-hospital treatment group (C). P(vent) probability of requiring mechanical ventilation. Antitoxin available at previous hospital was intramuscular equine origin only.

Table S4 Secondary outcomes in per-protocol populations

	Antitoxin	n		Summary statistic	Effect measure		(95% CI[6])	P-value
**Duration of intensive care unit stay (days)** [1]	Sham	132	Median (IQR)	16.0 (8.0, 23.0)				
	Intrathecal	132	Median (IQR)	13.0 (8.0, 21.0)				0.02
	Equine intramuscular	106	Median (IQR)	14.0 (8.0, 22.0)				
	Human intramuscular	109	Median (IQR)	13.0 (7.0, 22.0)				0.67
**Duration of hospital stay (days)** [1]	Sham	132	Median (IQR)	24.0 (17.8, 31.0)				
	Intrathecal	132	Median (IQR)	23.0 (18.0, 29.0)				0.11
	Equine intramuscular	106	Median (IQR)	23.0 (17.0, 30.0)				
	Human intramuscular	109	Median (IQR)	23.0 (17.0, 30.0)				0.83
**Duration of mechanical ventilation (days)** [2]	Sham	68	Median (IQR)	17.0 (11.0, 23.3)				
	Intrathecal	59	Median (IQR)	17.0 (12.5, 20.5)				0.50
	Equine intramuscular	47	Median (IQR)	17.0 (12.3, 22.8)				
	Human intramuscular	50	Median (IQR)	16.0 (11.0, 21.0)				0.77
**In hospital deaths**[2]	Sham	132	n(%)	4 (3.0%)		—	—	
	Intrathecal	132	n(%)	2 (1.5%)	OR	0.49	0.07, 2.57	0.42
	Equine intramuscular	106	n(%)	4 (3.8%)		—	—	
	Human intramuscular	109	n(%)	2 (1.8%)	OR	0.48	0.07, 2.50	0.40
**240 day deaths**[3]	Sham	131	n(%)	6 (4.6%)		—	—	
	Intrathecal	130	n(%)	4 (3.1%)	OR	0.66	0.16, 2.35	0.52
	Equine intramuscular	105	n(%)	6 (5.7%)		—	—	
	Human intramuscular	107	n(%)	4 (3.7%)	OR	0.63	0.16, 2.29	0.49

	Antitoxin	n		Summary statistic	Effect measure		(95% CI[6])	P-value
**240-day Rankin score. Number (%) severe (score>2)** [4]	Sham	131	n(%)	18 (14%)		—	—	
	Intrathecal	130	n(%)	10 (7.7%)	OR	0.52	0.22, 1.16	0.12
	Equine intramuscular	105	n(%)	14 (13%)		—	—	
	Human intramuscular	107	n(%)	9 (8.4%)	OR	0.60	0.24, 1.43	0.25
**Ventilator associated pneumonia (VAP)** [2*]	Sham	68	n(%)	30 (44%)		—	—	
	Intrathecal	59	n(%)	26 (44%)	OR	1.00	0.49, 2.02	>0.99
	Equine intramuscular	47	n(%)	20 (43%)		—	—	
	Human intramuscular	50	n(%)	25 (50%)	OR	1.35	0.61, 3.03	0.46
**Microbiologically confirmed VAP**[2*]	Sham	66	n(%)	24 (35%)		—	—	
	Intrathecal	59	n(%)	21 (36%)	OR	1.01	0.49, 2.10	0.97
	Equine intramuscular	47	n(%)	16 (34%)		—	—	
	Human intramuscular	50	n(%)	21 (42%)	OR	1.40	0.62, 3.23	0.42
**New antibiotics during hospitalization**[2]	Sham	132	n(%)	60 (45%)		—	—	
	Intrathecal	132	n(%)	50 (38%)	OR	0.73	0.45, 1.19	0.21
	Equine intramuscular	106	n(%)	39 (37%)		—	—	
	Human intramuscular	109	n(%)	44 (40%)	OR	1.16	0.67, 2.02	0.59
**Autonomic nervous system dysfunction**[2]	Sham	132	n(%)	27 (20%)		—	—	
	Intrathecal	132	n(%)	25 (19%)	OR	0.91	0.49, 1.67	0.76
	Equine intramuscular	106	n(%)	21 (20%)		—	—	
	Human intramuscular	109	n(%)	21 (19%)	OR	0.97	0.49, 1.90	0.92

	Antitoxin	n		Summary statistic	Effect measure		(95% CI[6])	P-value
**Dose of pipecuronium (mg) [5*]**	Sham	68	Median (IQR)	364 (158, 646)		—	—	
	Intrathecal	59	Median (IQR)	452 (223, 633)	Beta	1.5	-1.7, 4.7	0.35
	Equine intramuscular	47	Median (IQR)	365 (132, 632)		—	—	
	Human intramuscular	50	Median (IQR)	444 (114, 713)	Beta	0.4	-3.5, 4.2	0.85
**Duration of pipecuronium (days)[5*]**	Sham	68	Median (IQR)	11.5 (6.0, 17.3)		—	—	
	Intrathecal	59	Median (IQR)	13.0 (8.0, 17.0)	Beta	0.9	-1.5, 3.4	0.44
	Equine intramuscular	47	Median (IQR)	11.5 (5.3, 18.0)		—	—	
	Human intramuscular	50	Median (IQR)	11.0 (5.5, 17.0)	Beta	0.5	-2.5, 3.5	0.74
**Dose of benzodiazepines (mg) [5]**	Sham	132	Median (IQR)	4784 (1683, 9209)		—	—	
	Intrathecal	132	Median (IQR)	4600 (1827, 8080)	Beta	-1.2	-8.5, 6.2	0.76
	Equine intramuscular	106	Median (IQR)	3457 (1416, 7709)		—	—	
	Human intramuscular	109	Median (IQR)	5588 (1686, 9021)	Beta	6.4	-1.8, 14.7	0.13
**Duration of benzodiazepines (days)[5]**	Sham	132	Median (IQR)	1.40 (1.27, 1.51)		—	—	
	Intrathecal	132	Median (IQR)	1.38 (1.28, 1.48)	Beta	-0.02	-0.1, 0.03	0.41
	Equine intramuscular	106	Median (IQR)	1.38 (1.26, 1.49)		—	—	
	Human intramuscular	109	Median (IQR)	1.38 (1.26, 1.49)	Beta	-0.005	-0.064, 0.054	0.87
**ICU Cost(USD)[5**]**	Sham	132	Median (IQR)	1160 (263, 2649)		—	—	
	Intrathecal	132	Median (IQR)	669 (272, 2256)	Beta	-0.1	-0.2, 0.1	0.16
	Equine intramuscular	106	Median (IQR)	721 (240, 2512)		—	—	
	Human intramuscular	109	Median (IQR)	744 (271, 2656)	Beta	0.02	-0.13, 0.16	0.83

	Antitoxin	n		Summary statistic	Effect measure		(95% CI[6])	P-value
**Hospital Cost(USD)[5**]**	Sham	132	Median (IQR)	1225 (380, 2730)		—	—	
	Intrathecal	132	Median (IQR)	753 (386, 2387)	Beta	-0.08	-0.20, 0.03	0.15
	Equine IM	106	Median (IQR)	791 (356, 2598)		—	—	
	Human intramuscular	109	Median (IQR)	843 (351, 2737)	Beta	0.01	-0.12, 0.14	0.92

P values correspond to statistical analysis as described in the ***Statistical Appendix** and Statistical Analysis Plan*. Summary statistics of median (IQR) and N (%) are given to aid interpretation.

[1] P value relates to cause-specific cumulative incidence tested using Gray's log-rank test.

[2] P value relates In-hospital mortality was assessed with a logistic regression model.

[3] P value relates to Kaplan-Meier curves and compared using the log-rank test.

[4] P value relates proportional odds logistic regression model.

[5] P value relates to linear regression model. Based on the Box-Cox diagnoses, beta coefficients of arms were compared on a log transformation scale for ICU costs and hospital costs, and on a square root transformation scale for dose of pipecuronium and benzodiazepines to reduce non-normality of the errors in linear regression models.

[6] CI Confidence Interval

*In those receiving mechanical ventilation

** Costs exclude antitoxin

Figure S7 Secondary outcomes – cardiovascular parameters: intrathecal and intramuscular (IM) interventions (intention-to-treat populations)

Intrathecal allocation | Intramuscular allocation

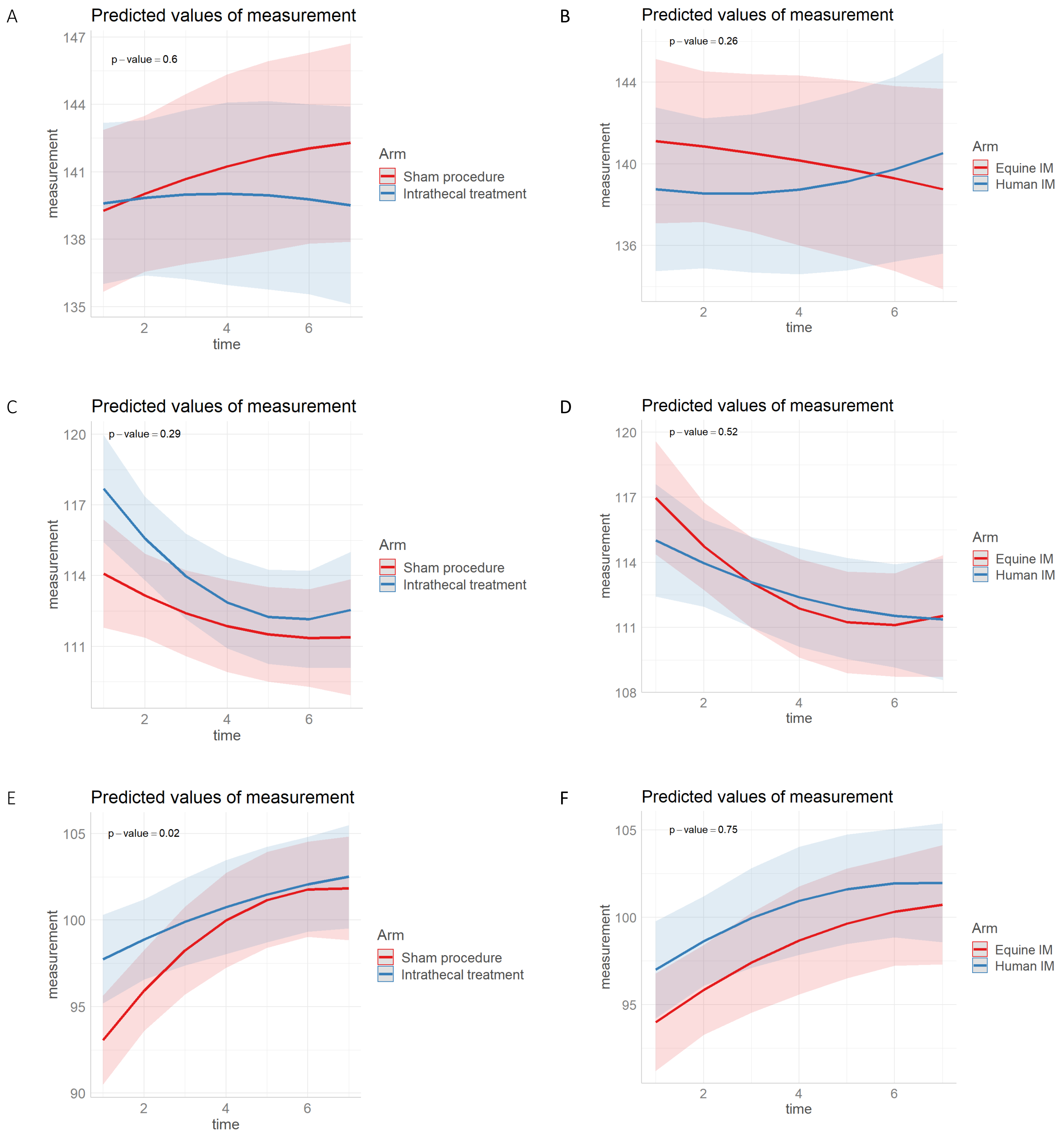

Intrathecal allocation

Intramuscular allocation

G

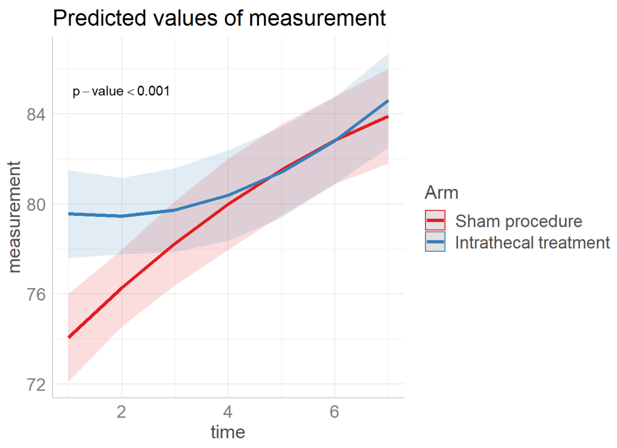

H

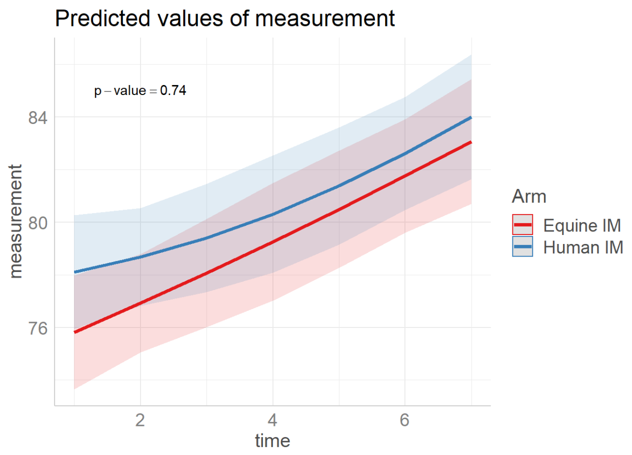

Figure shows predicted values of measurement over time for: maximum systolic blood pressure (A & B); minimum systolic blood pressure (C,D); maximum heart rate (E,F); minimum heart rate (G, H)

The p-values refer to the test for interaction between intervention and time on the outcome

Figure S8 Cardiovascular parameters for the intramuscular (IM) intervention including population receiving antitoxin at previous hospital.

Figure S8 Systolic blood pressure and heart rate in the intramuscular intervention including population receiving antitoxin at previous hospital

Daily maximum systolic blood pressure

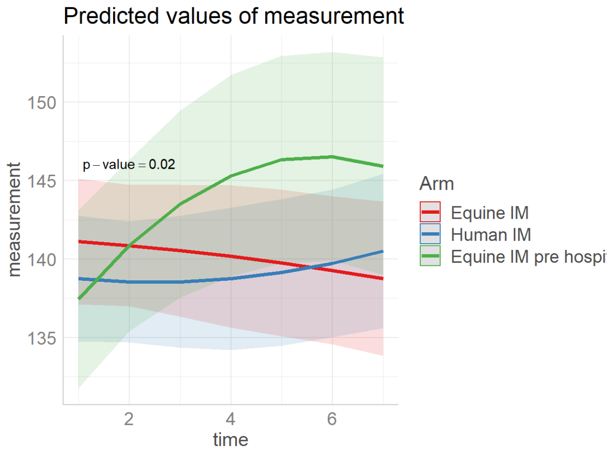

Daily maximum heart rate

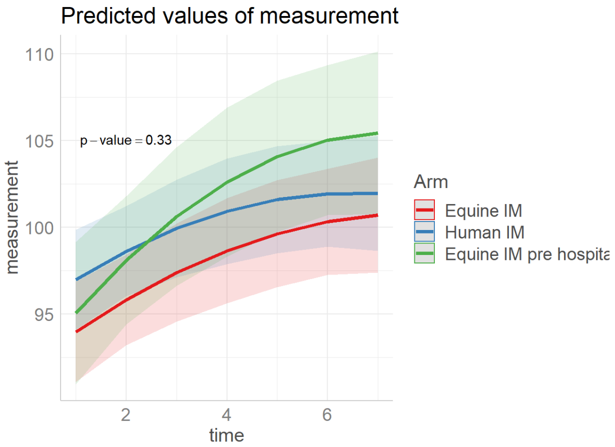

Daily minimum systolic blood pressure

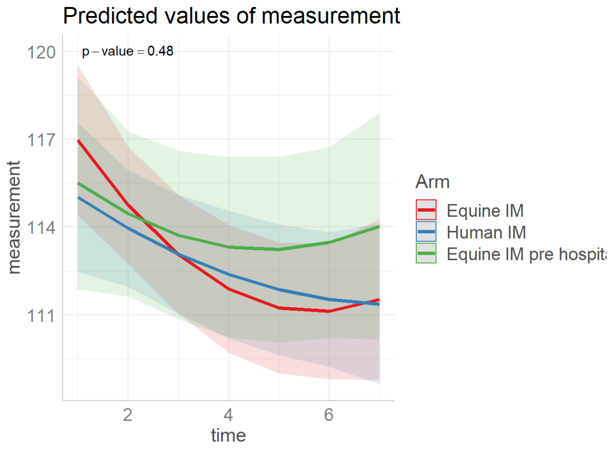

Daily minimum heart rate

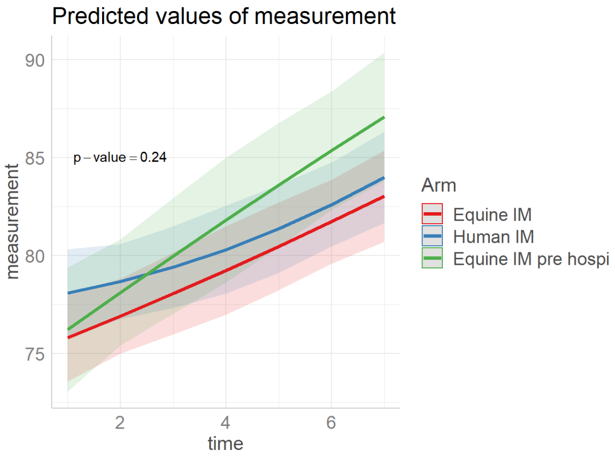

Figure shows predicted values of measurement over time. The p-values refer to the test for interaction between intervention and time on the outcome.

Antitoxin at previous hospital was intramuscular equine origin only.

Figures S9a-c Duration of intensive care unit (ICU) stay

Figure S9a Time to ICU discharge (Intrathecal intervention intention-to-treat and per-protocol populations)

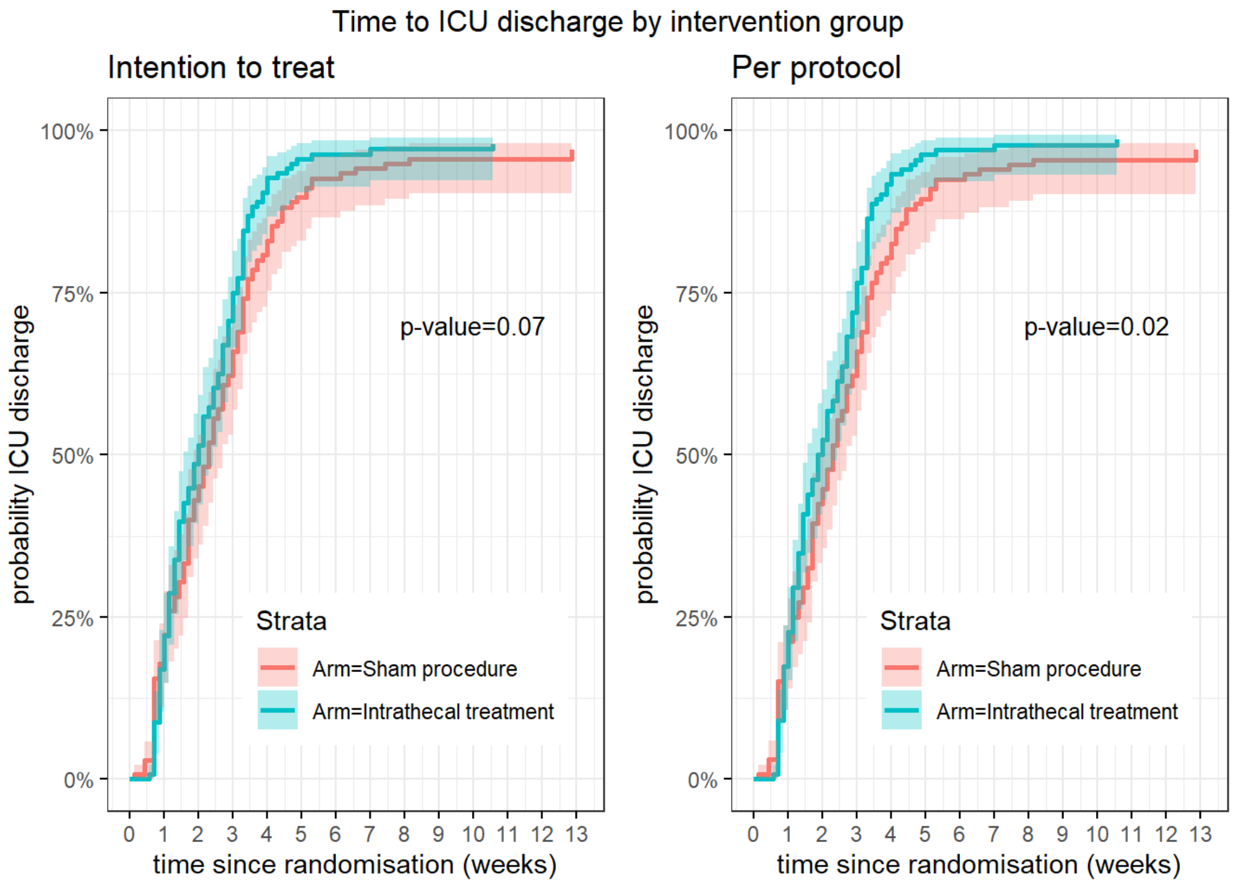

P value relates to cause-specific cumulative incidence tested using Gray's log-rank test

Figure S9b Time to ICU discharge (Intramuscular (IM) intervention: intention-to-treat and per-protocol populations)

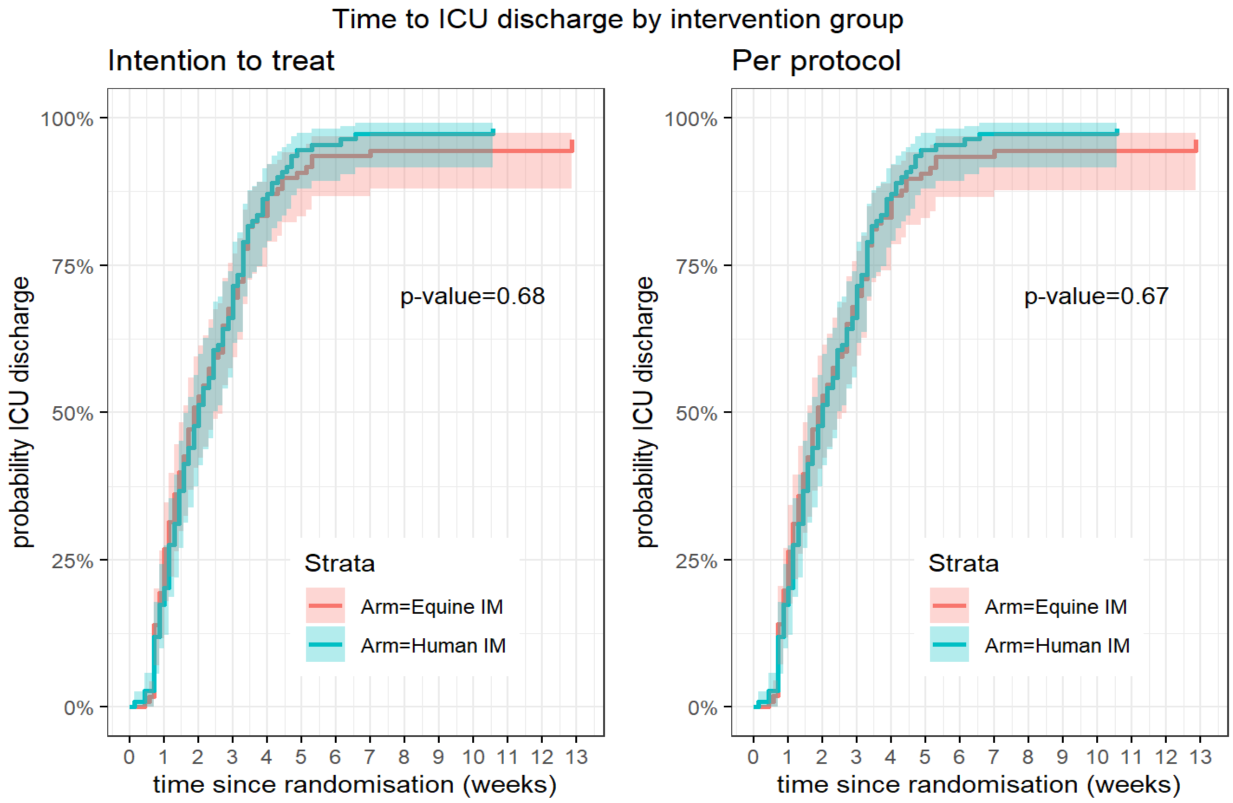

P value relates to cause-specific cumulative incidence tested using Gray's log-rank test

Figure S9c Time to ICU discharge (Intramuscular intervention including pre-hospital intramuscular antitoxin population)

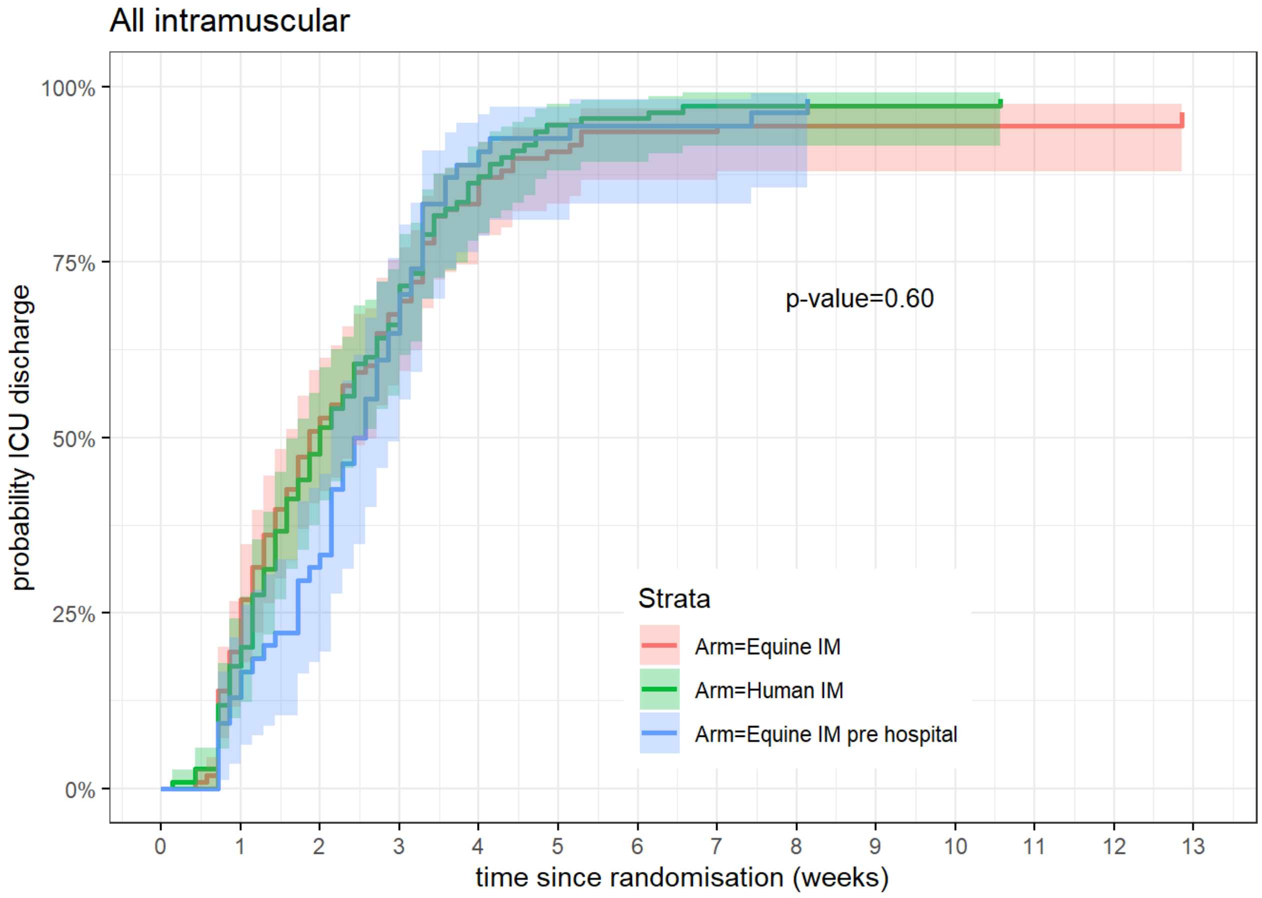

P value relates to cause-specific cumulative incidence tested using Gray's log-rank test

Antitoxin at previous hospital was intramuscular equine origin only.

Figures S10a-c: Duration of hospital stay

Figure S10a Time to hospital discharge (Intrathecal intervention intention-to-treat and per-protocol populations)

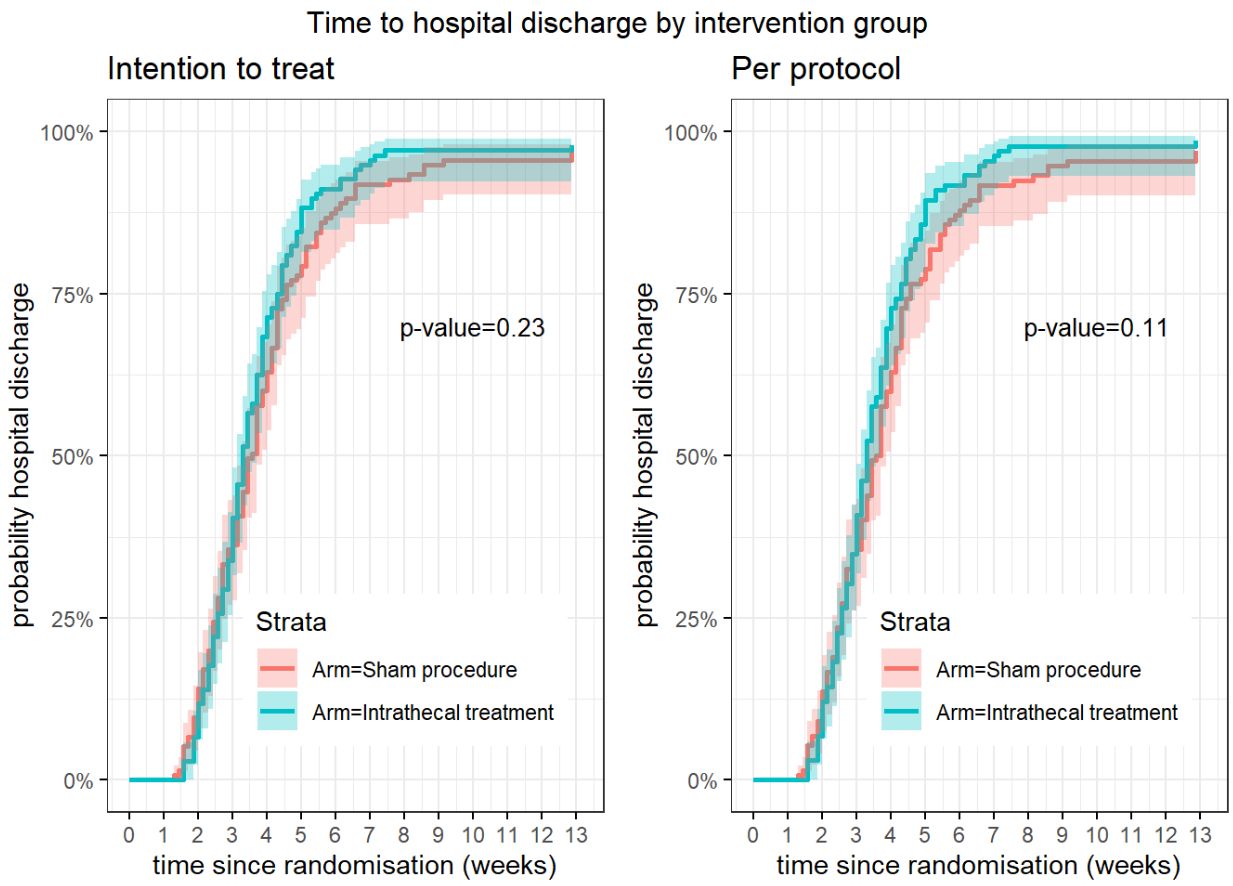

P value relates to cause-specific cumulative incidence tested using Gray's log-rank test

Figure S10b Time to hospital discharge (Intramuscular (IM) intervention intention-to-treat and per-protocol populations)

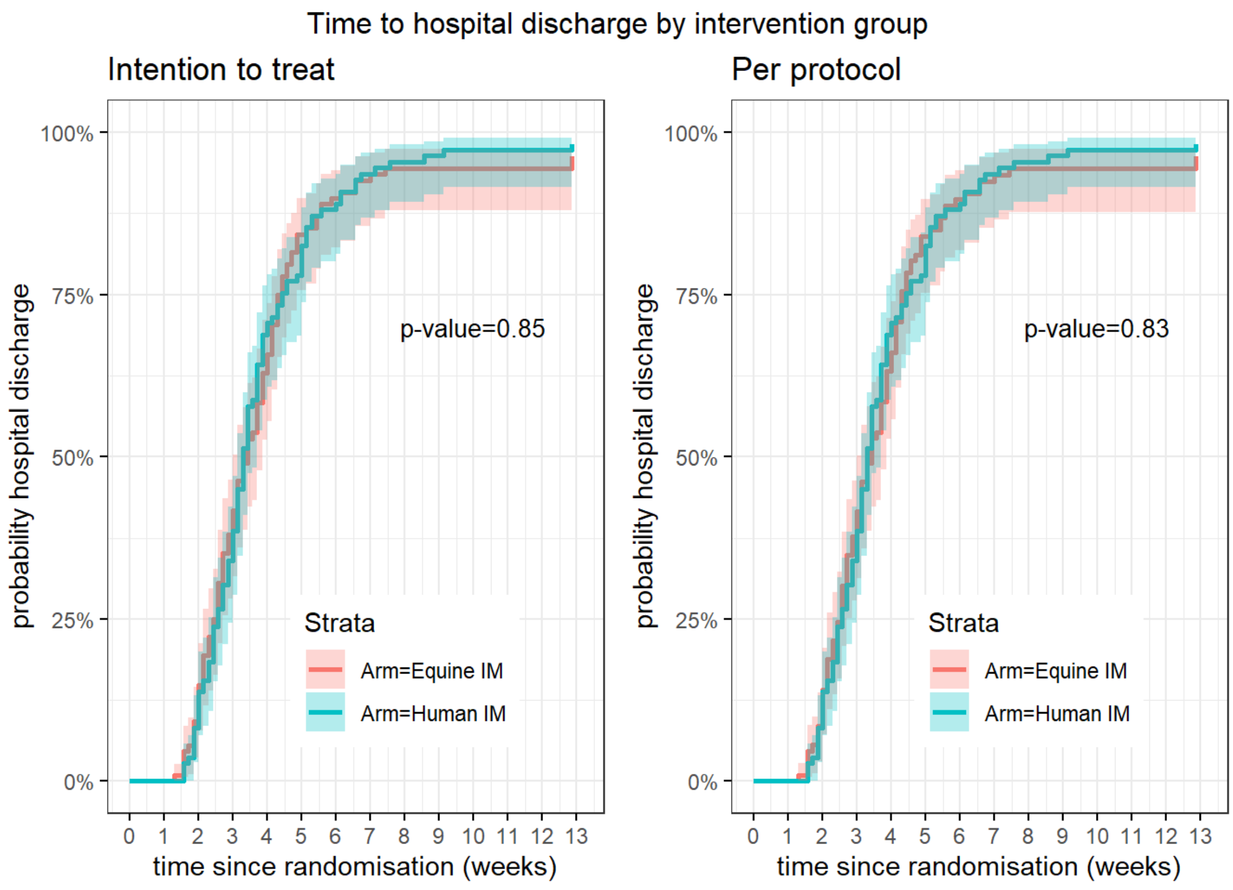

P value relates to cause-specific cumulative incidence tested using Gray's log-rank test

Figure S10c Time to hospital discharge (Intramuscular (IM) intervention including pre-hospital intramuscular antitoxin population)

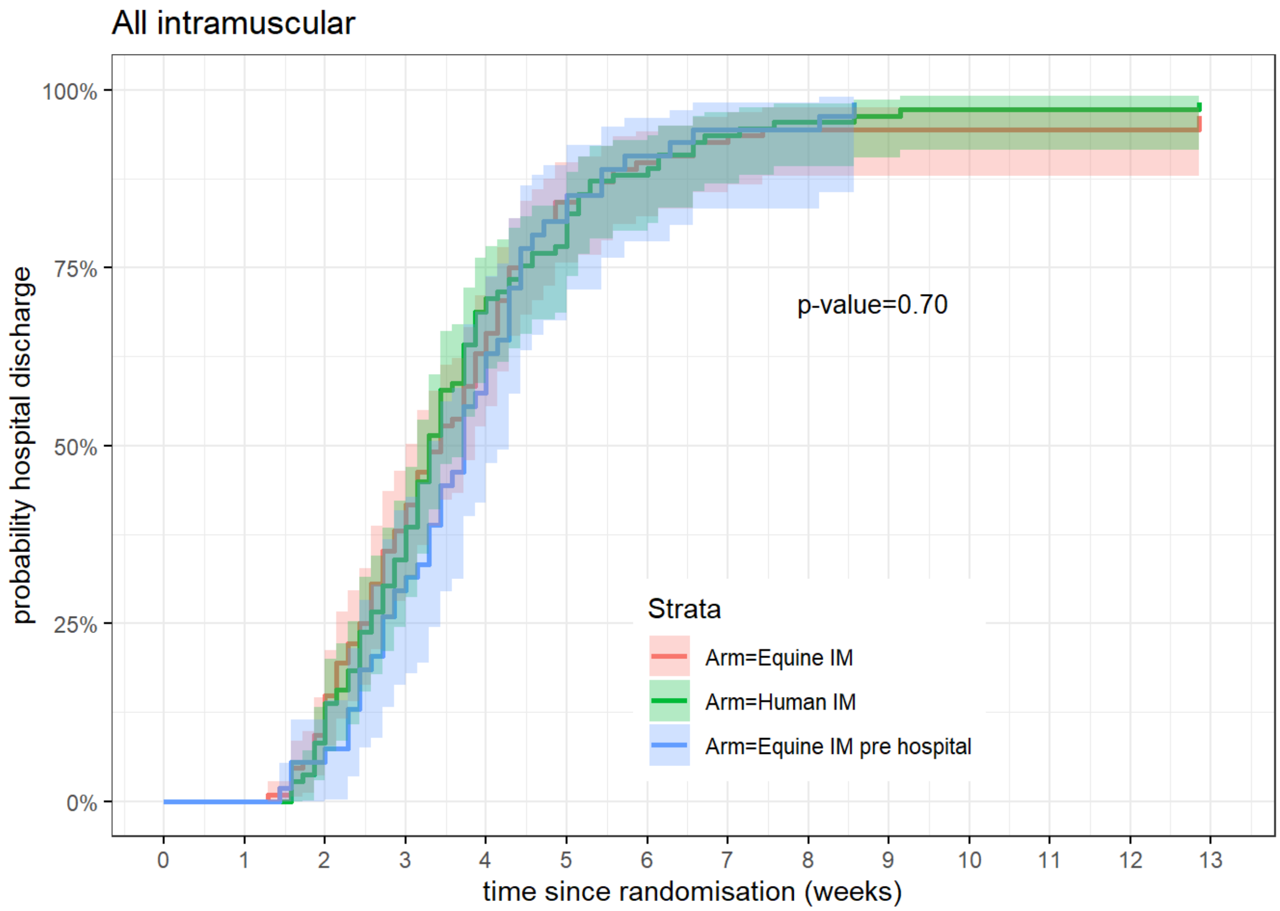

P value relates to cause-specific cumulative incidence tested using Gray's log-rank test

Antitoxin at previous hospital was intramuscular equine origin only.

Figures S11a-c Time from treatment to start mechanical ventilation

Figure S11a Time to start mechanical ventilation (Intrathecal intervention intention-to-treat and per-protocol populations)

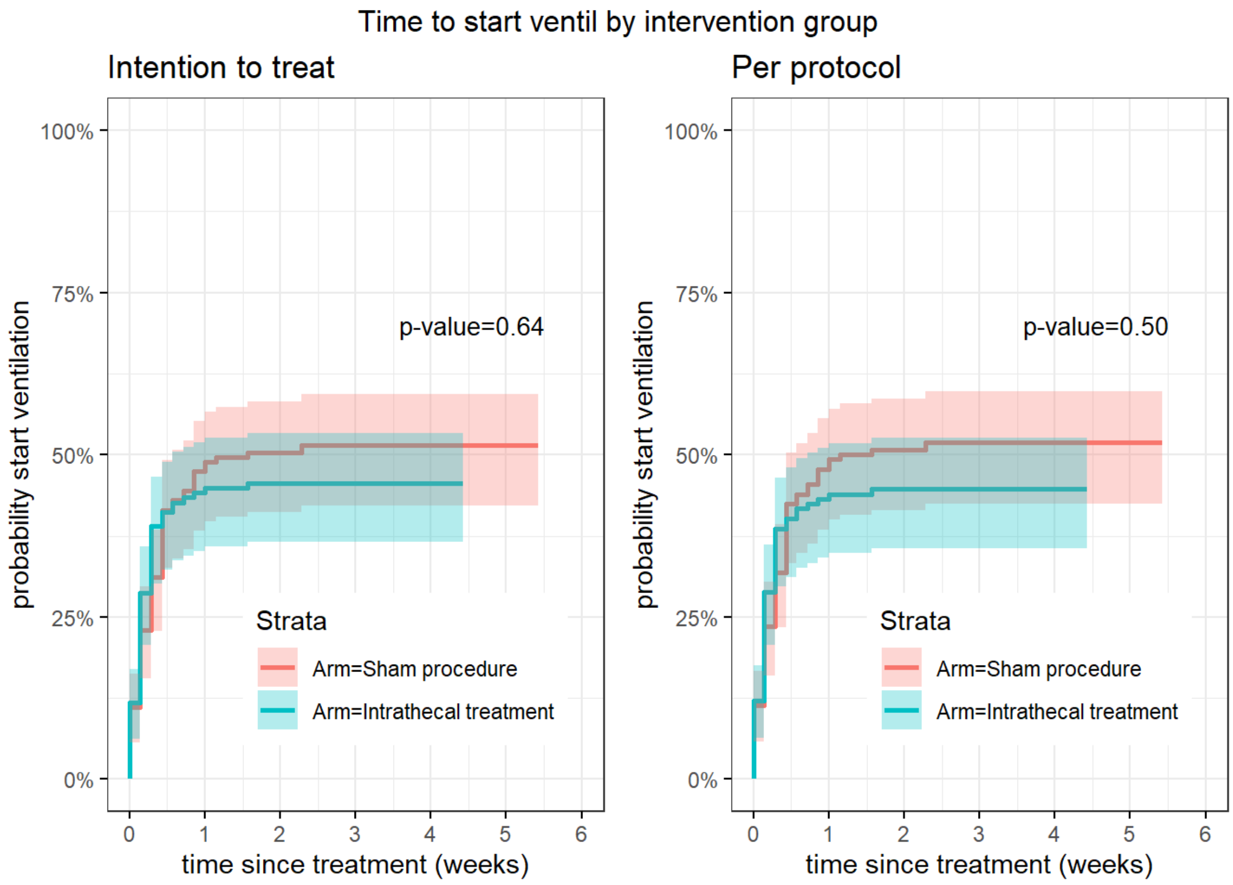

P value relates to cause-specific cumulative incidence tested using Gray's log-rank test

Figure S11b Time to start mechanical ventilation (Intramuscular intervention (IM) intention-to-treat and per-protocol populations)

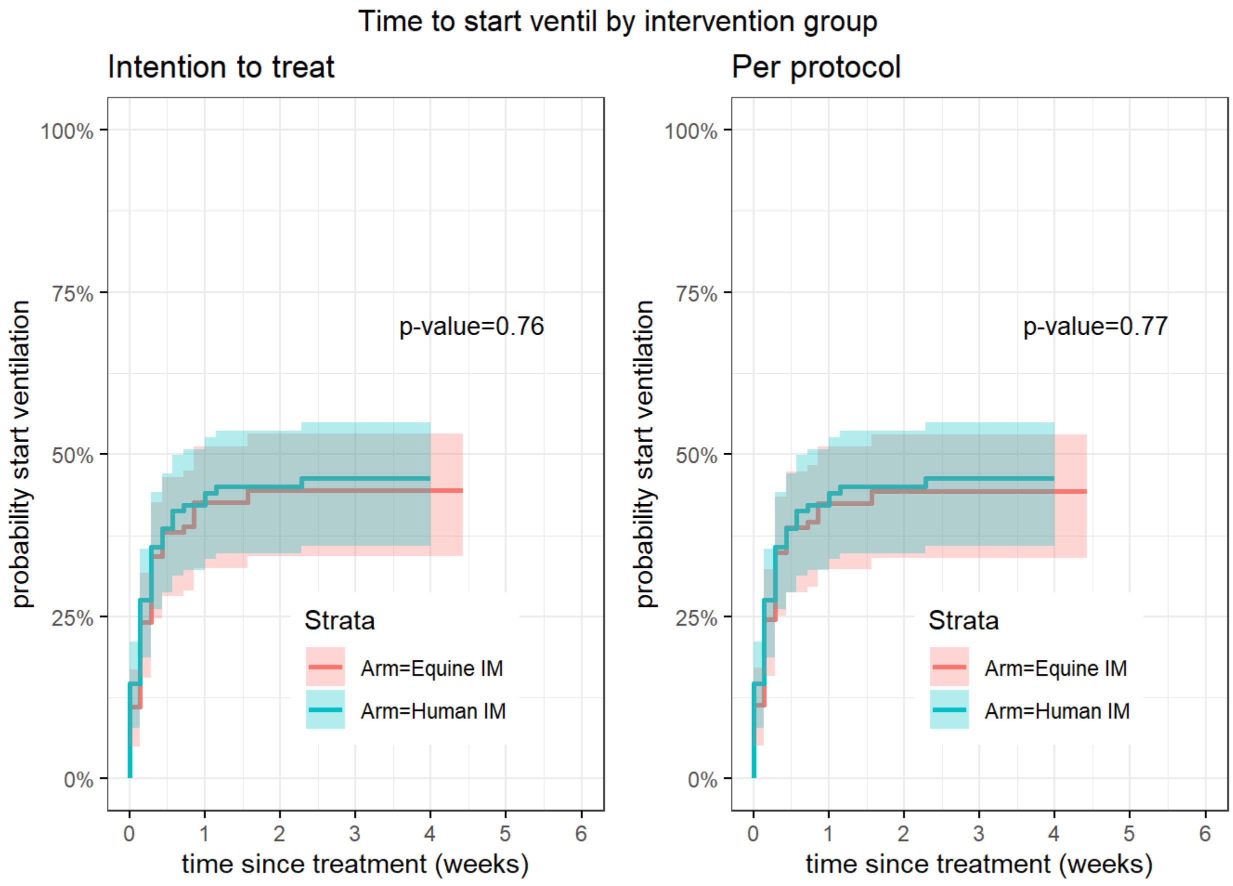

P value relates to cause-specific cumulative incidence tested using Gray's log-rank test

Figure S11c Time to start mechanical ventilation (Intramuscular (IM) intervention including pre-hospital intramuscular antitoxin population)

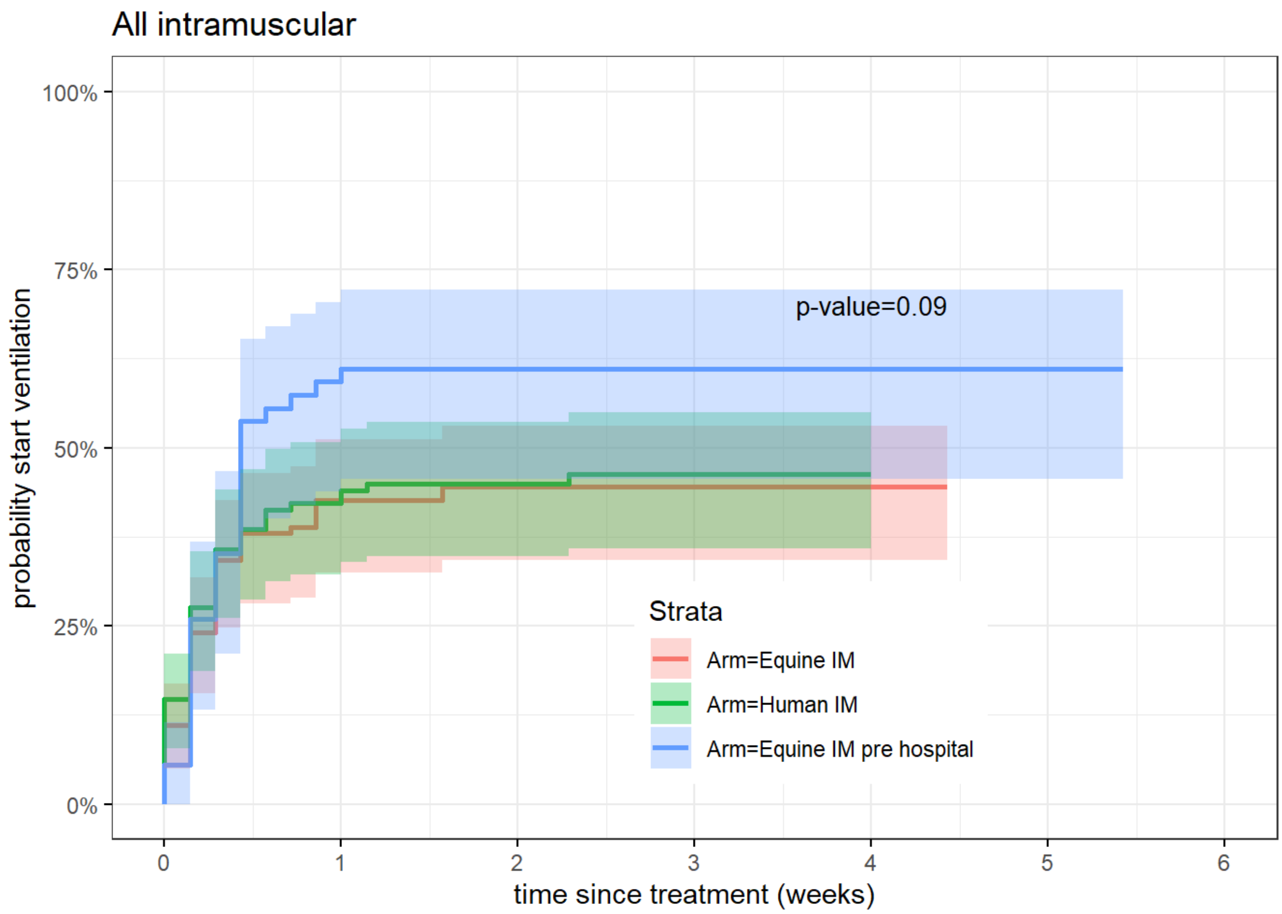

P value relates to cause-specific cumulative incidence tested using Gray's log-rank test

Antitoxin at previous hospital was intramuscular equine origin only.

Figures S12a-c Duration of mechanical ventilation

Figure S12a Duration of mechanical ventilation (Intrathecal intervention intention-to-treat and per-protocol populations)

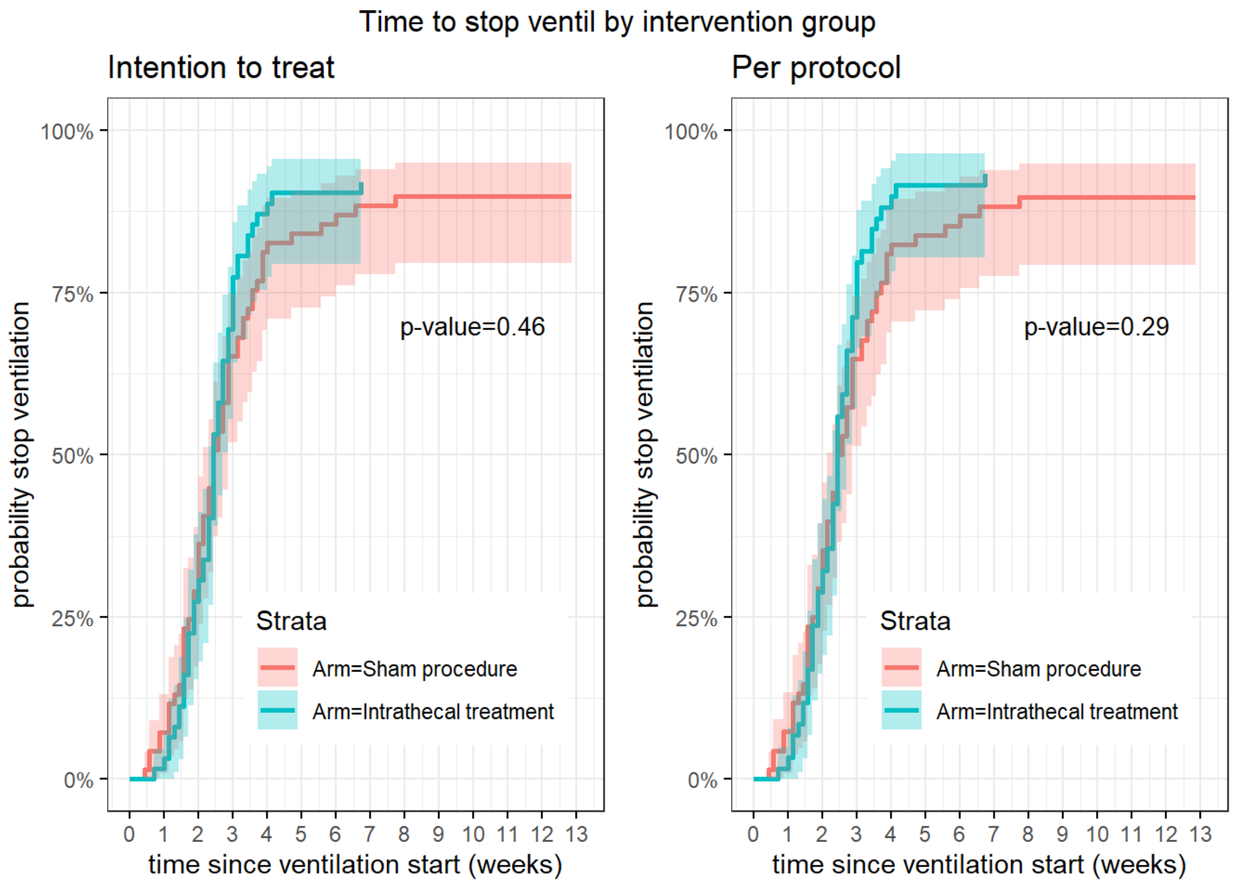

P value relates to cause-specific cumulative incidence tested using Gray's log-rank test

Figure S12b Duration of mechanical ventilation (Intramuscular (IM) population intention-to-treat and per-protocol population)

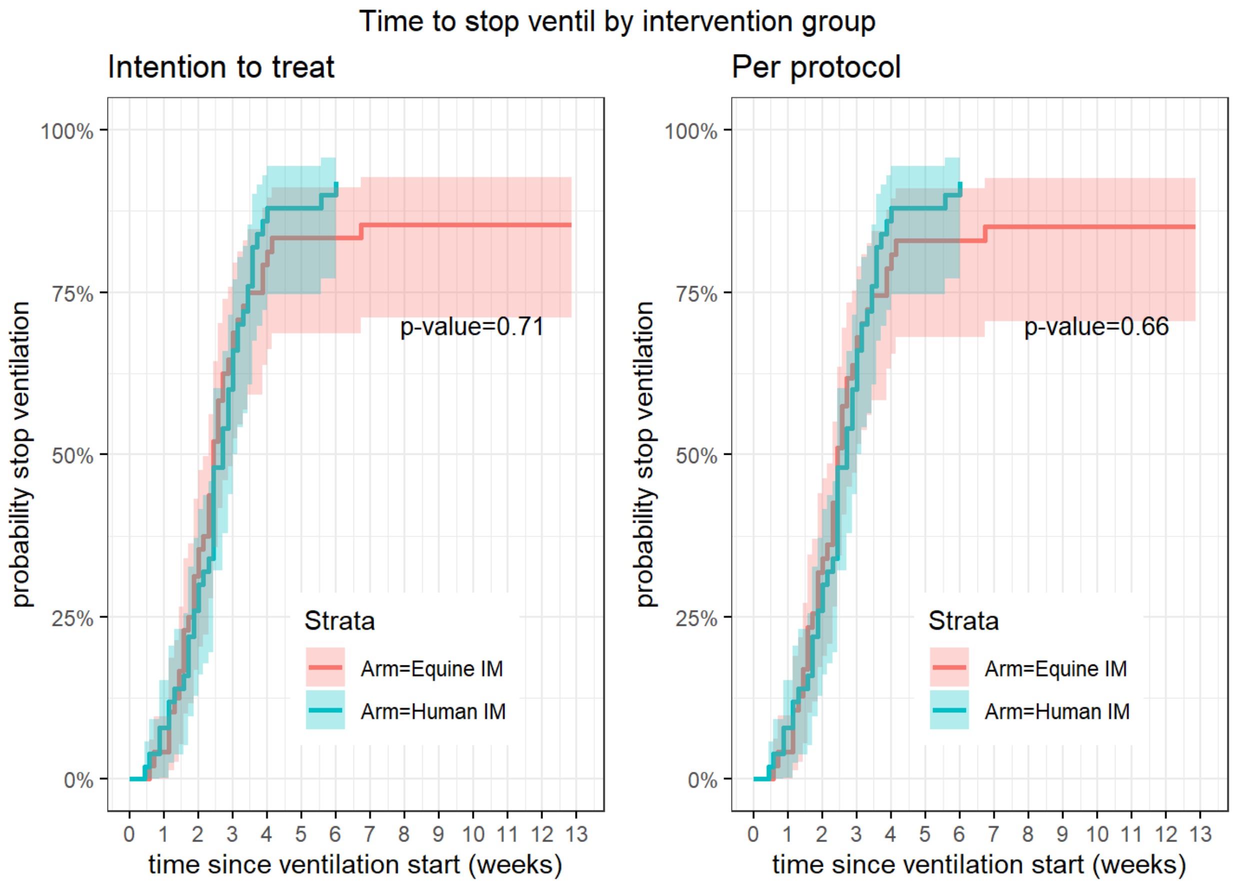

P value relates to cause-specific cumulative incidence tested using Gray's log-rank test

Figure S12c Duration of mechanical ventilation (Intramuscular (IM) intervention including pre-hospital intramuscular antitoxin treatment population)

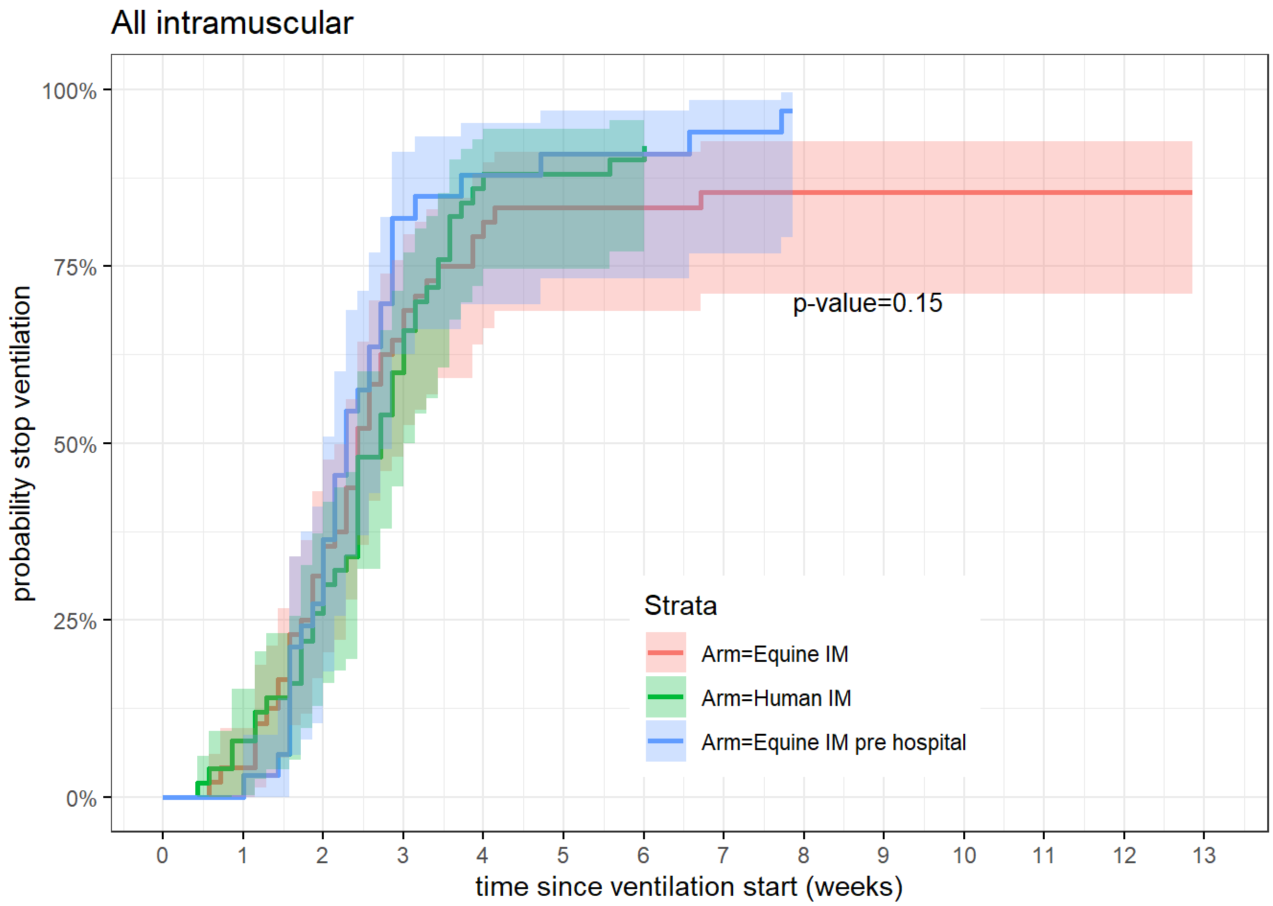

P value relates to cause-specific cumulative incidence tested using Gray's log-rank test

Antitoxin at previous hospital was intramuscular equine origin only.

Table S5: in-hospital mortality including population receiving antitoxin at previous hospital

Table S5 In-hospital mortality for intramuscular intervention including population receiving antitoxin at previous hospital

Variable	Not died, (n = 264)	Died (n = 7)	OR[1] for mortality	95% CI[2]	P-value
Arm (N = 271)					**0.65**
Equine intramuscular	104 (96%)	4 (3.7%)	—	—	
Human intramuscular	107 (98%)	2 (1.8%)	0.49	0.07, 2.54	0.41
Antitoxin in previous hospital[3]	53 (98%)	1 (1.9%)	0.49	0.02, 3.42	0.53

[1]OR = Odds Ratio; [2]CI = Confidence Interval. [3]Antitoxin at previous hospital was intramuscular equine origin only.

Table S6: 240-day mortality including population receiving antitoxin at previous hospital

Table S6 240-day mortality for Intramuscular intervention including population receiving antitoxin at previous hospital

Variable	Not died (n = 264)	Died (n = 7)	OR[1] for mortality	95% CI[2]	P-value
Arm (N = 271)					**0.49**
Equine intramuscular	102 (94%)	6 (5.6%)	—	—	
Human intramuscular	105 (96%)	4 (3.7%)	0.65	0.16, 2.33	0.51
Antitoxin in previous hospital[3]	53 (98%)	1 (1.9%)	0.32	0.02, 1.94	0.30

[1]OR = Odds Ratio; [2]CI = Confidence Interval. [3]Antitoxin at previous hospital was intramuscular equine origin only.

Figures S13 a-c Kaplan Meier Curves for 240-day mortality

Figure S13a 240-day mortality (Intrathecal intervention: intention-to-treat and per-protocol populations)

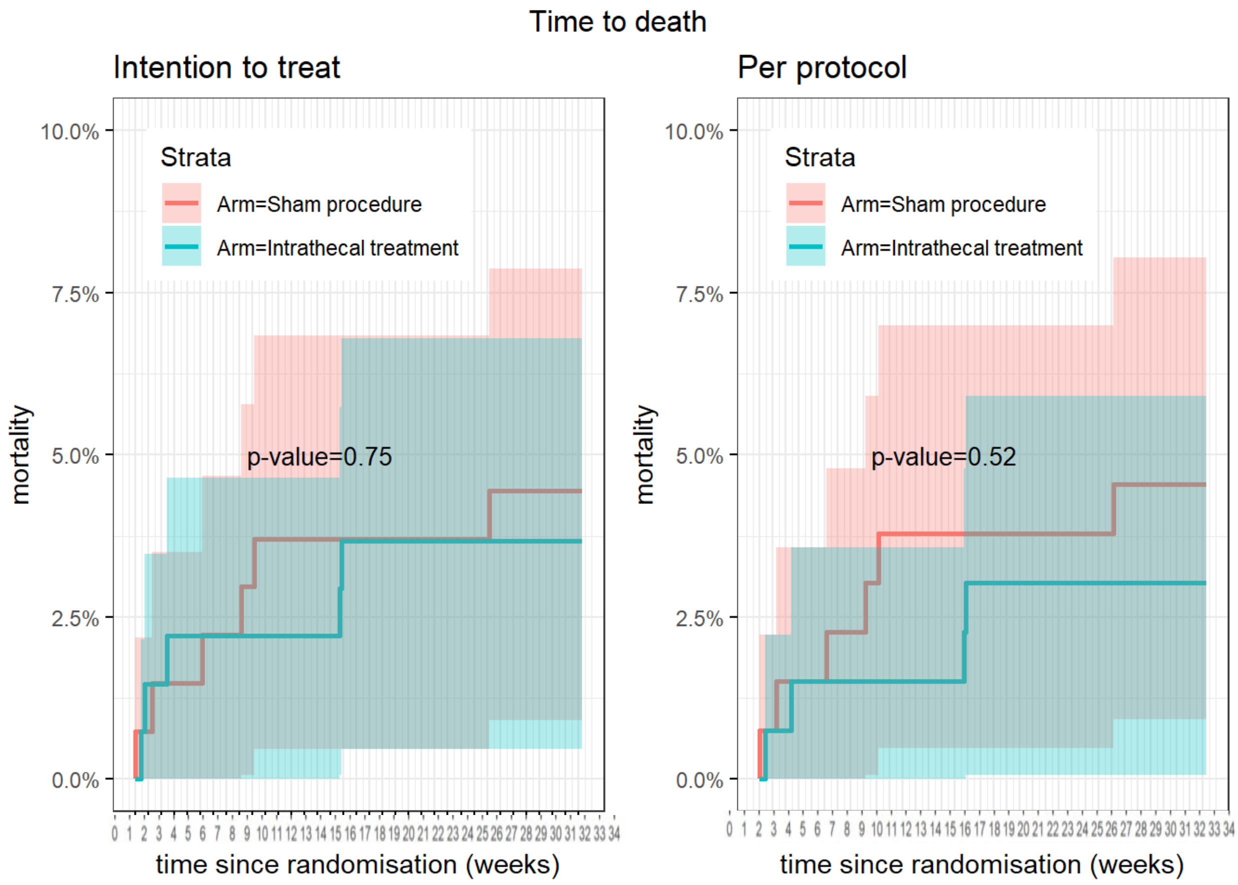

P value relates to log-rank test

Figure S13b 240-day mortality (Intramuscular, IM, intervention intention-to-treat and per-protocol populations)

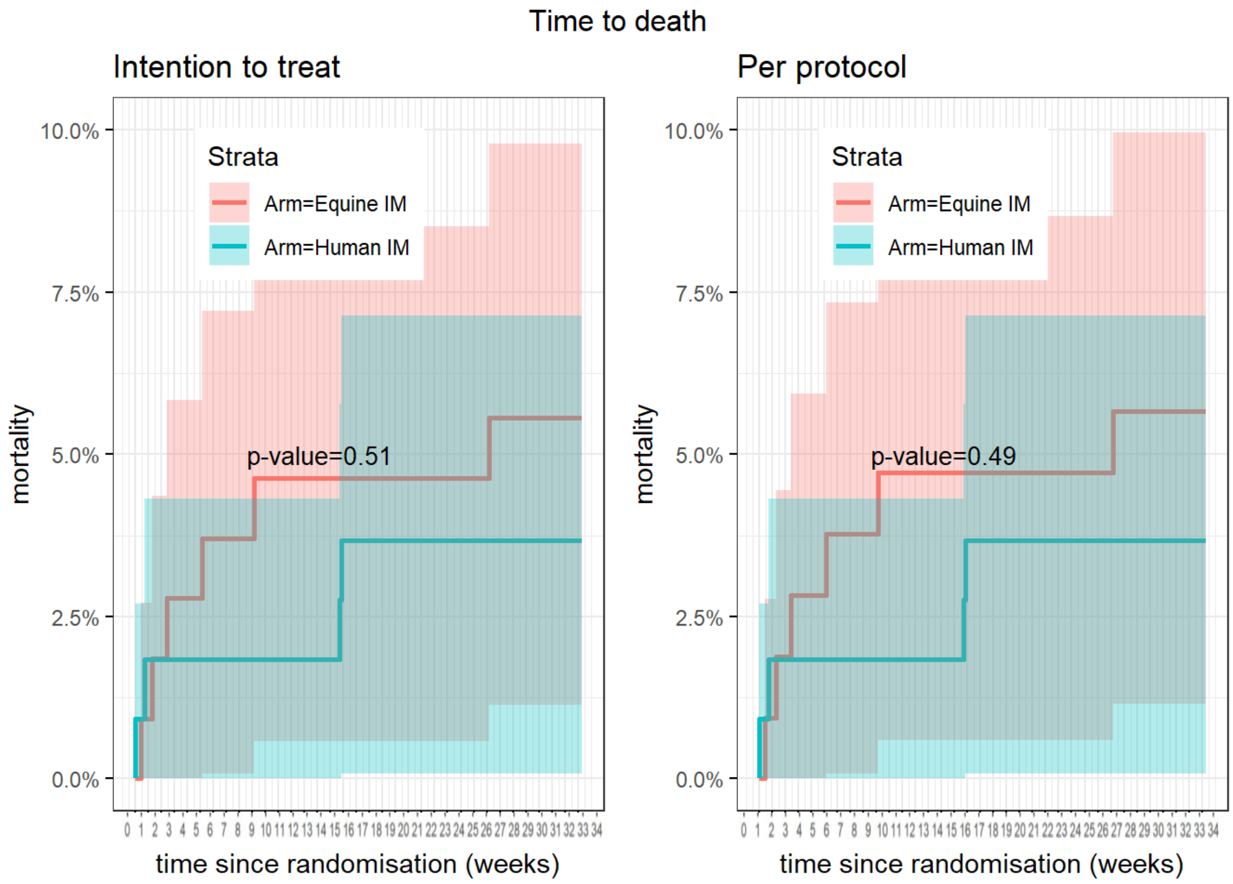

P value relates to log-rank test

Antitoxin at previous hospital was intramuscular equine origin only.

Figure S13c: 240-day mortality (Intramuscular (IM) intervention including population receiving antitoxin at previous hospital)

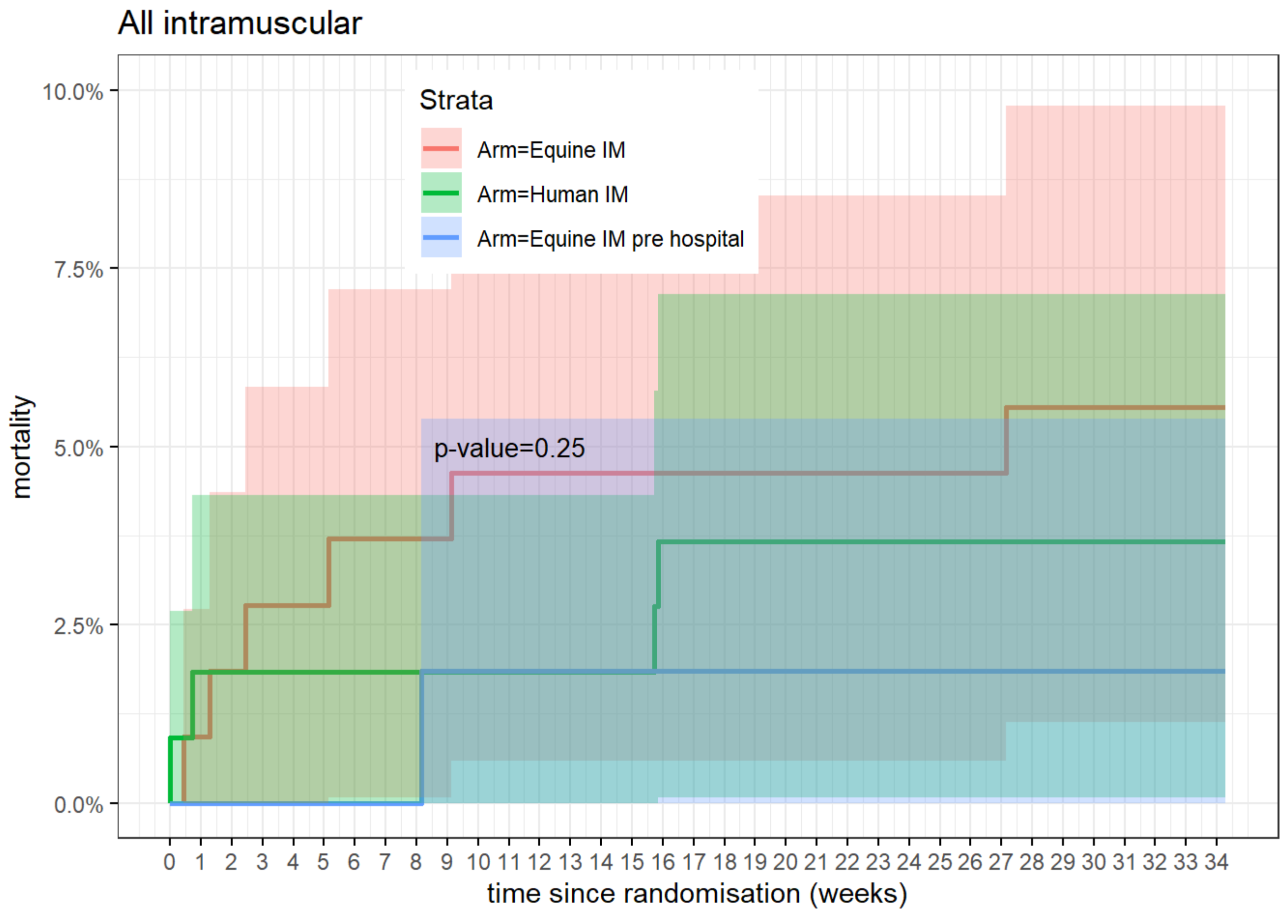

P value relates to log-rank test

Antitoxin at previous hospital was intramuscular equine origin only.

Tables S7 a-f: 240-day disability

Table S7a 240-day disability – Rankin scores (Intrathecal intervention intention-to-treat population)

Variable	Modified Rankin Scores for Neurologic Disability (0-6)						OR[1] for disability	95% CI[2]	P-value
	0 (n = 204)	1 (n = 35)	2 (n = 13)	4 (n = 2)	5 (n = 3)	6 (n = 11)			
Arm (N = 268)									
Sham procedure	95 (71%)	21 (16%)	8 (6%)	2 (1.5%)	2 (1.5%)	6 (4.5%)			
Intrathecal treatment	109 (81%)	14 (10%)	5 (3.7%)	0 (0%)	1 (0.7%)	5 (3.7%)	0.56	0.32, 0.99	0.048

[1]OR = Odds Ratio of being in a higher category of reported Modified Rankin Score for Neurologic Disability between interventions; [2]CI = Confidence Interval

Table S7b 240-day disability – Rankin scores (Intramuscular intervention intention-to-treat population)

Variable	Modified Rankin Scores for Neurologic Disability (0-6)						OR[1] for disability	95% CI[2]	P-value
	0 (n = 164)	1 (n = 27)	2 (n = 9)	4 (n = 1)	5 (n = 3)	6 (n = 10)			
Arm (N = 214)									
Equine intramuscular	80 (75%)	13 (12%)	5 (4.7%)	1 (0.9%)	2 (1.9%)	6 (5.6%)			
Human intramusuclar	84 (79%)	14 (13%)	4 (3.7%)	0 (0%)	1 (0.9%)	4 (3.7%)	0.78	0.41, 1.47	0.45

[1]OR = Odds Ratio of being in a higher category of reported Modified Rankin Score for Neurologic Disability between interventions; [2]CI = Confidence Interval

Table S7c 240 day disability – Rankin scores (Intrathecal intervention per-protocol population)

Variable	Modified Rankin Scores for Neurologic Disability (0-6)						OR[1] for disability	95% CI[2]	P-value
	0 (n = 198)	1 (n = 35)	2 (n = 13)	4 (n = 2)	5 (n = 3)	6 (n = 10)			
Arm (N = 261)									
Sham procedure	92 (70%)	21 (16%)	8 (6.1%)	2 (1.5%)	2 (1.5%)	6 (4.6%)			
Intrathecal treatment	106 (82%)	14 (11%)	5 (3.8%)	0 (0%)	1 (0.8%)	4 (3.1%)	0.53	0.30, 0.94	0.032

[1]OR = Odds Ratio of being in a higher category of reported Modified Rankin Scores for Neurologic Disability between interventions; [2]CI = Confidence Interval

Table S7d 240-gday disability – Rankin scores (Intramuscular intervention per-protocol population)

Variable	Modified Rankin Scores for Neurologic Disability (0-6)						OR[1] for disability	95% CI[2]	P-value
	0 (n = 162)	1 (n = 27)	2 (n = 9)	4 (n = 1)	5 (n = 3)	6 (n = 10)			
Arm (N = 212)									
Equine intramuscular	78 (74%)	13 (12%)	5 (4.8%)	1 (0.9%)	2 (1.9%)	6 (5.7%)			
Human intramuscular	84 (79%)	14 (13%)	4 (3.7%)	0 (0%)	1 (0.9%)	4 (3.7%)	0.76	0.40, 1.43	0.40

[1]OR = Odds Ratio of being in a higher category of reported Modified Rankin Score for Neurologic Disability between interventions; [2]CI = Confidence Interval

Table S7e 240-day disability – Rankin scores (Intramuscular population including population receiving antitoxin at previous hospital)

Variable	0 (n = 204)	1 (n = 35)	2 (n = 13)	4 (n = 2)	5 (n = 3)	6 (n = 11)	OR[1] for disability	95% CI[2]	P-value
Arm (N = 268)									0.88
Equine intramuscular	80 (75%)	13 (12%)	5 (4.7%)	1 (0.9%)	2 (1.9%)	6 (5.6%)			
Human intramuscular	84 (79%)	14 (13%)	4 (3.7%)	0 (0%)	1 (0.9%)	4 (3.7%)	0.78	0.41, 1.46	0.44
Antitoxin at previous hospital[3]	40 (74%)	8 (15%)	4 (7.4%)	1 (1.9%)	0 (0%)	1 (1.9%)	0.98	0.46, 2.03	0.96

[1]OR = Odds Ratio of being in a higher category of reported Modified Rankin Score for Neurologic Disability between interventions; [2]CI = Confidence Interval. [3]Antitoxin at previous hospital was intramuscular equine origin only.

Table S7f 240 day disability (Severe vs. Mild) intramuscular intervention including population receiving antitoxin at previous hospital

Variable	Mild (0-1) (n = 239)	Severe (2-6) (n = 29)	OR[1] for severe disability	95% CI[2]	P-value
Arm (N = 268)					**0.54**
Equine intramuscular	93 (87%)	14 (13%)	—	—	
Human intramuscular	98 (92%)	9 (8.4%)	0.61	0.24, 1.46	0.27
Antitoxin at previous hospital[3]	48 (89%)	6 (11%)	0.83	0.28, 2.21	0.72

[1]OR = Odds Ratio of being in a higher category of reported Modified Rankin Score for Neurologic Disability between interventions; [2]CI = Confidence Interval. [3]Antitoxin at previous hospital was intramuscular equine origin only.

Tables S8a&b Ventilator associated pneumonia (VAP) in ventilated patients, intramuscular intervention including population receiving antitoxin at previous hospital

Table S8a Ventilator associated pneumonia (VAP) in patients ventilated, intramuscular intervention including population receiving antitoxin at previous hospital

Variable	No VAP[1] (n = 75)	VAP (n = 56)	OR[2] for VAP	95% CI[3]	P-value
Arm (N = 131)					**0.31**
Equine intramuscular	28 (58%)	20 (42%)	—	—	
Human intramuscular	25 (50%)	25 (50%)	1.40	0.63, 3.13	0.41
Antitoxin at previous hospital[4]	22 (67%)	11 (33%)	0.70	0.27, 1.75	0.45

[1]VAP = Ventilator associated pneumonia; [2]OR = Odds Ratio; [3]CI = Confidence Interval. [4]Antitoxin at previous hospital was intramuscular equine origin only.

Table S8b Microbiologically confirmed VAP – intramuscular intervention including population receiving antitoxin at previous hospital

Variable	No VAP[1] (n = 86)	VAP (n = 45)	OR[2] for VAP	95% CI[3]	P-value
Arm (N = 131)					**0.24**
Equine intramuscular	32 (67%)	16 (33%)	—	—	
Human intramuscular	29 (58%)	21 (42%)	1.45	0.64, 3.33	0.38
Antitoxin at previous hospital[4]	25 (76%)	8 (24%)	0.64	0.23, 1.70	0.38

[1]VAP = Ventilator associated pneumonia; [2]OR = Odds Ratio; [3]CI = Confidence Interval. [4]Antitoxin at previous hospital was intramuscular equine origin only.

Table S9 Clinical syndrome of autonomic nervous system dysfunction (ANSD) intramuscular intervention including population receiving antitoxin at previous hospital

Table S9 Clinical syndrome of autonomic nervous system dysfunction (ANSD) intramuscular intervention including population receiving antitoxin at previous hospital

Variable	No ANSD [1] (n = 215)	ANSD (n = 56)	OR[2] for ANSD	95% CI[3]	P-value
**Arm (N = 271)**					**0.78**
Equine intramuscular	86 (80%)	22 (20%)	—	—	
Human intramuscular	88 (81%)	21 (19%)	0.93	0.48, 1.82	0.84
Antitoxin at previous hospital[4]	41 (76%)	13 (24%)	1.24	0.56, 2.68	0.59

[1]ANSD = Autonomic nervous system dysfunction; [2]OR = Odds Ratio; [3]CI = Confidence Interval. [4]Antitoxin at previous hospital was intramuscular equine origin only.

Table S10 New antibiotic prescription during ICU stay intramuscular intervention including population receiving antitoxin at previous hospital

Table S10 New antibiotic prescription during ICU stay (excluding antibiotics for tetanus or initial entry site infection) intramuscular intervention including population receiving antitoxin at previous hospital

Variable	No AB [1], (n = 159)	AB (n = 112)	OR[2] for AB	95% CI[3]	P-value
**Arm (N = 271)**					**0.19**
Equine intramuscular	68 (63%)	40 (37%)	—	—	
Human intramuscular	65 (60%)	44 (40%)	1.15	0.67, 1.99	0.61
Antitoxin at previous hospital[4]	26 (48%)	28 (52%)	1.83	0.95, 3.57	0.073

[1]AB = Antibiotic prescription; [2]OR = Odds Ratio; [3]CI = Confidence Interval. [4]Antitoxin at previous hospital was intramuscular equine origin only.

Figures S14a-c Total dose of pipecuronium during hospital stay (for patients ventilated)

Figure S14a Total dose of pipecuronium Intrathecal intervention intention-to-treat (left hand panel) and per-protocol (right hand panel) populations

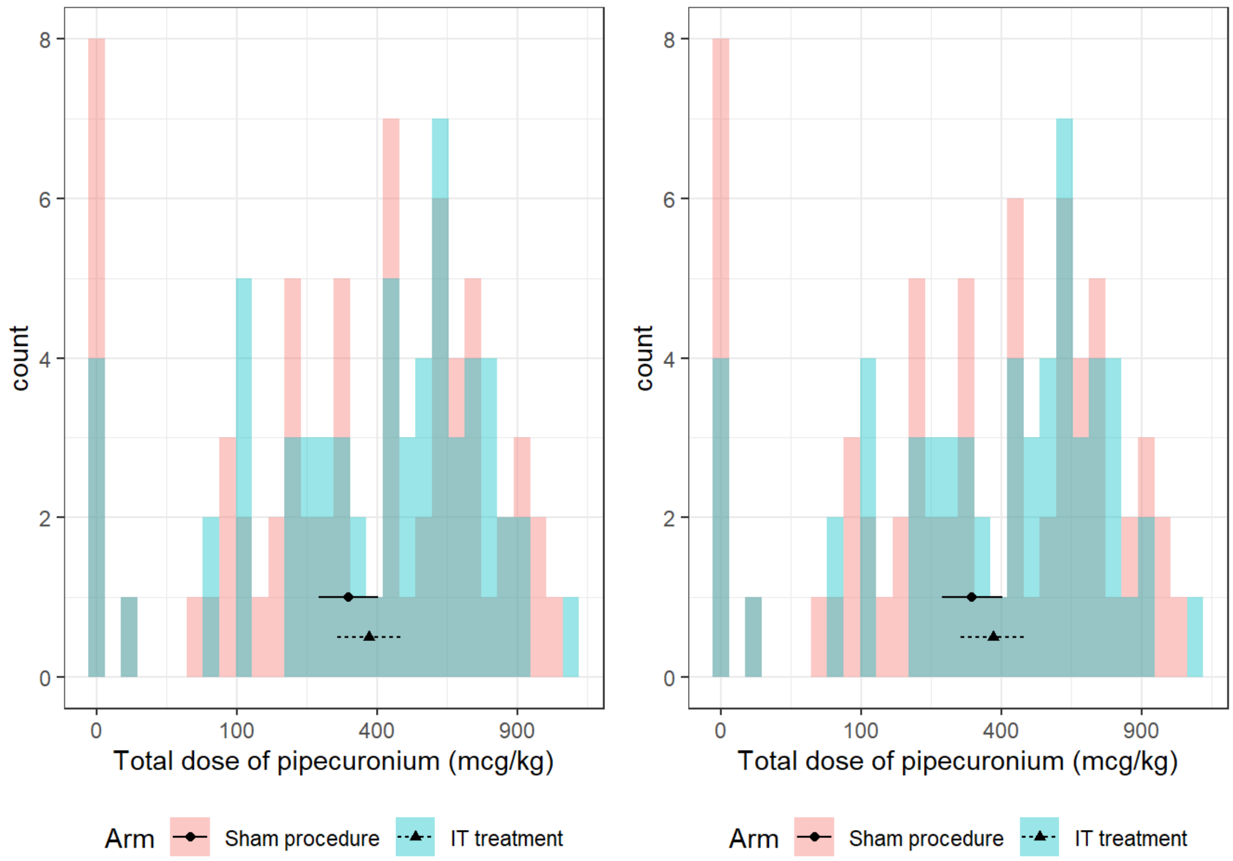

Mean values and 95% confidence intervals were shown in the central horizontal lines of the histograms for intervention arms

Figure S14b Total dose of pipecuronium Intramuscular(IM) intervention intention-to-treat (left hand panel) and per-protocol (right hand panel) populations

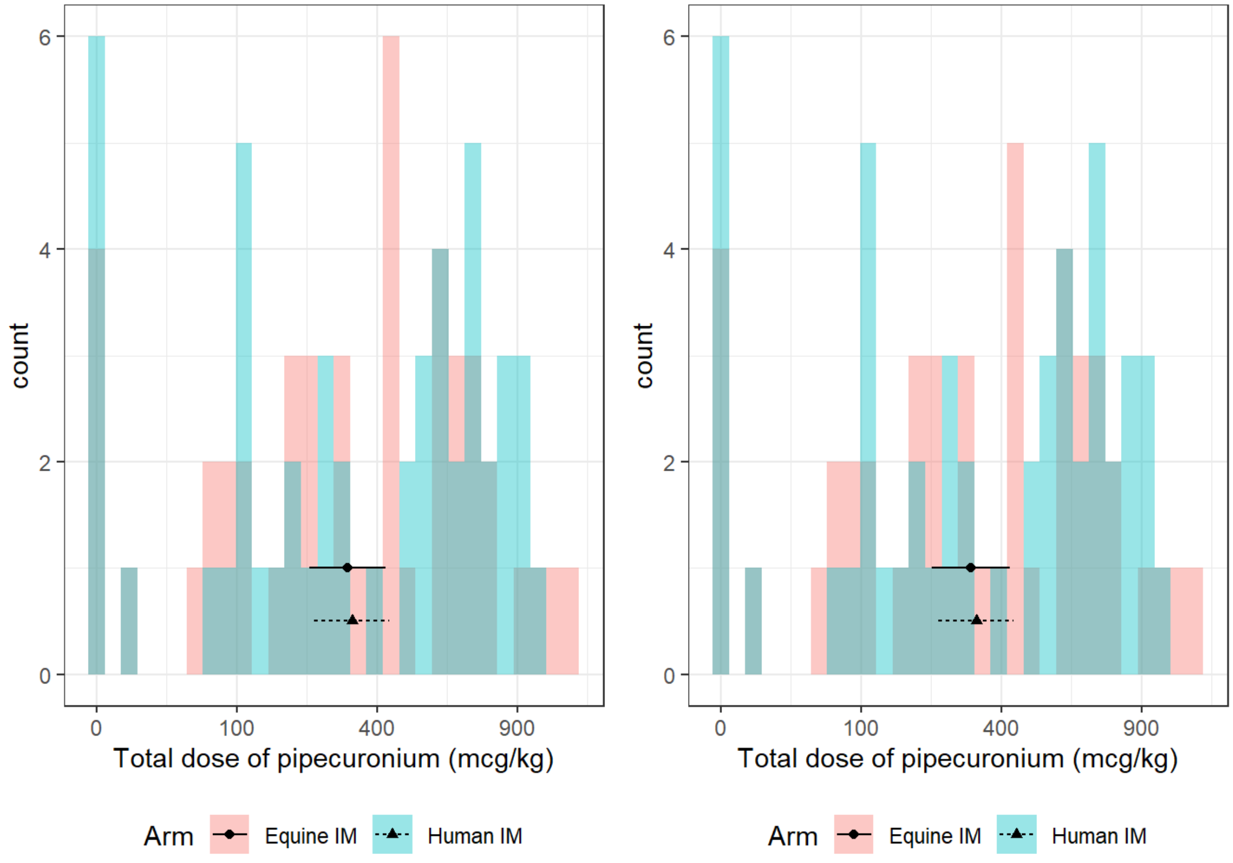

Mean values and 95% confidence intervals were shown in the central horizontal lines of the histograms for intervention arms

Figure S14c Total dose of pipecuronium (Intramuscular (IM) intervention including population receiving antitoxin at previous hospital)

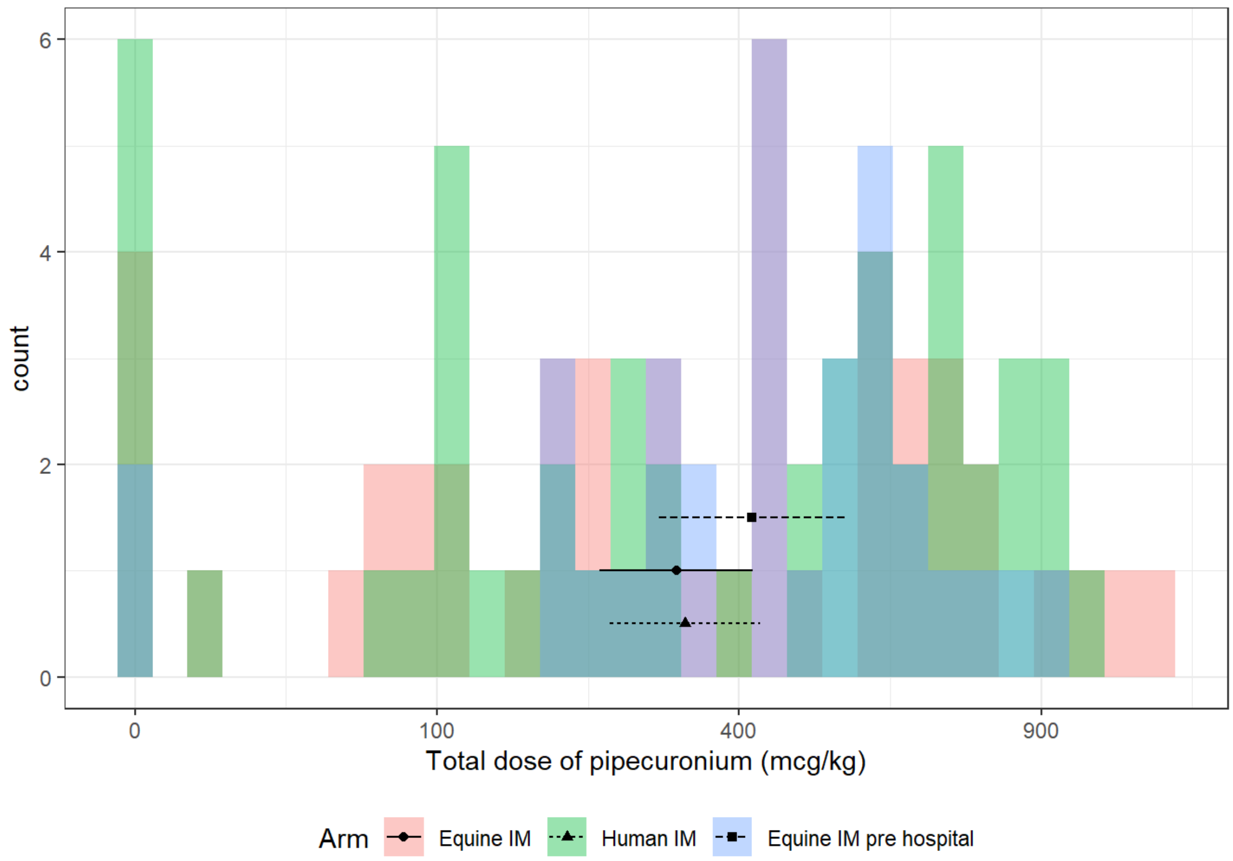

Mean values and 95% confidence intervals were shown in the central horizontal lines of the histograms for intervention arms

Antitoxin at previous hospital was intramuscular equine origin only.

Figures S15 a-c Total duration of pipecuronium (for patients ventilated)

Figure S15a Total duration of pipecuronium (left hand panel) intrathecal intervention and per-protocol (right hand panel) populations

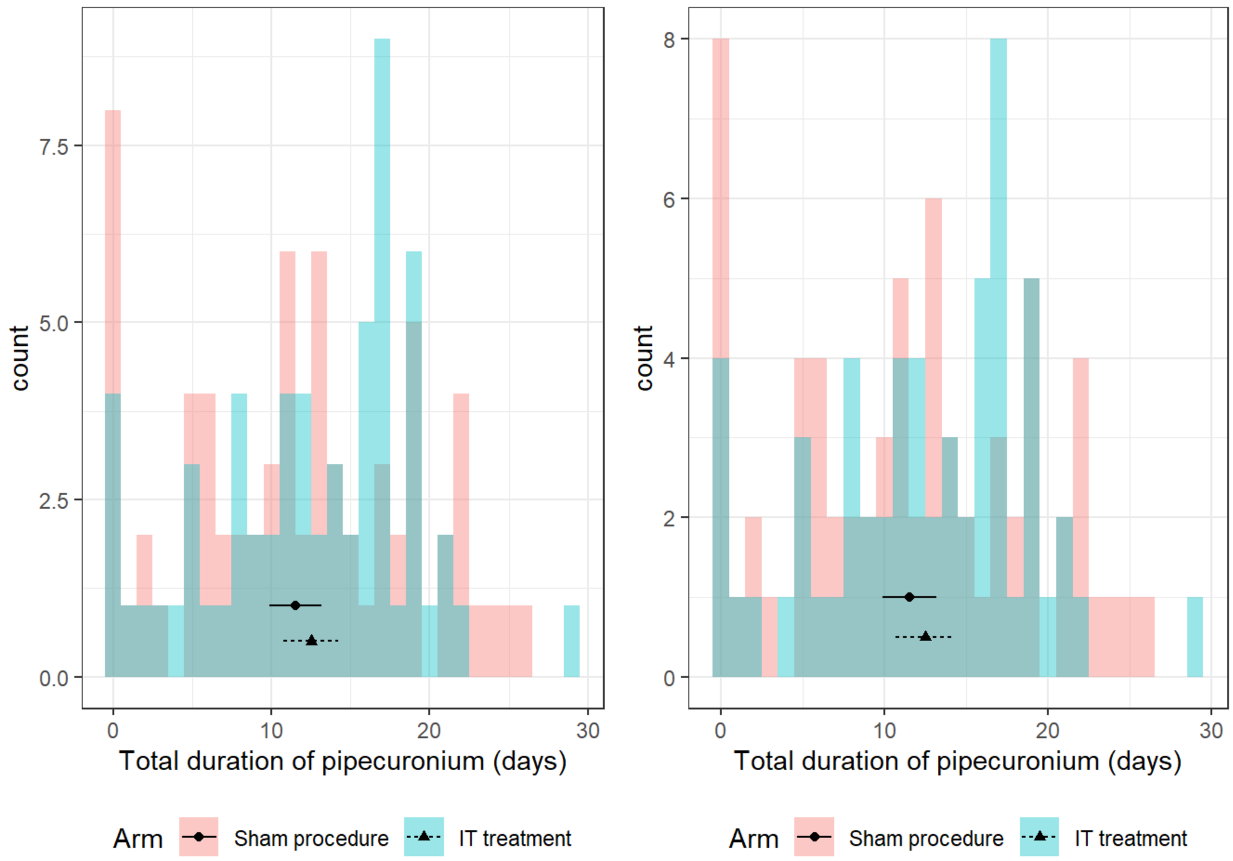

Mean values and 95% confidence intervals were shown in the central horizontal lines of the histograms for intervention arms

Figure S15b Total duration of pipecuronium intramuscular (IM) intention-to-treat (left hand panel) and per-protocol (right hand panel) populations)

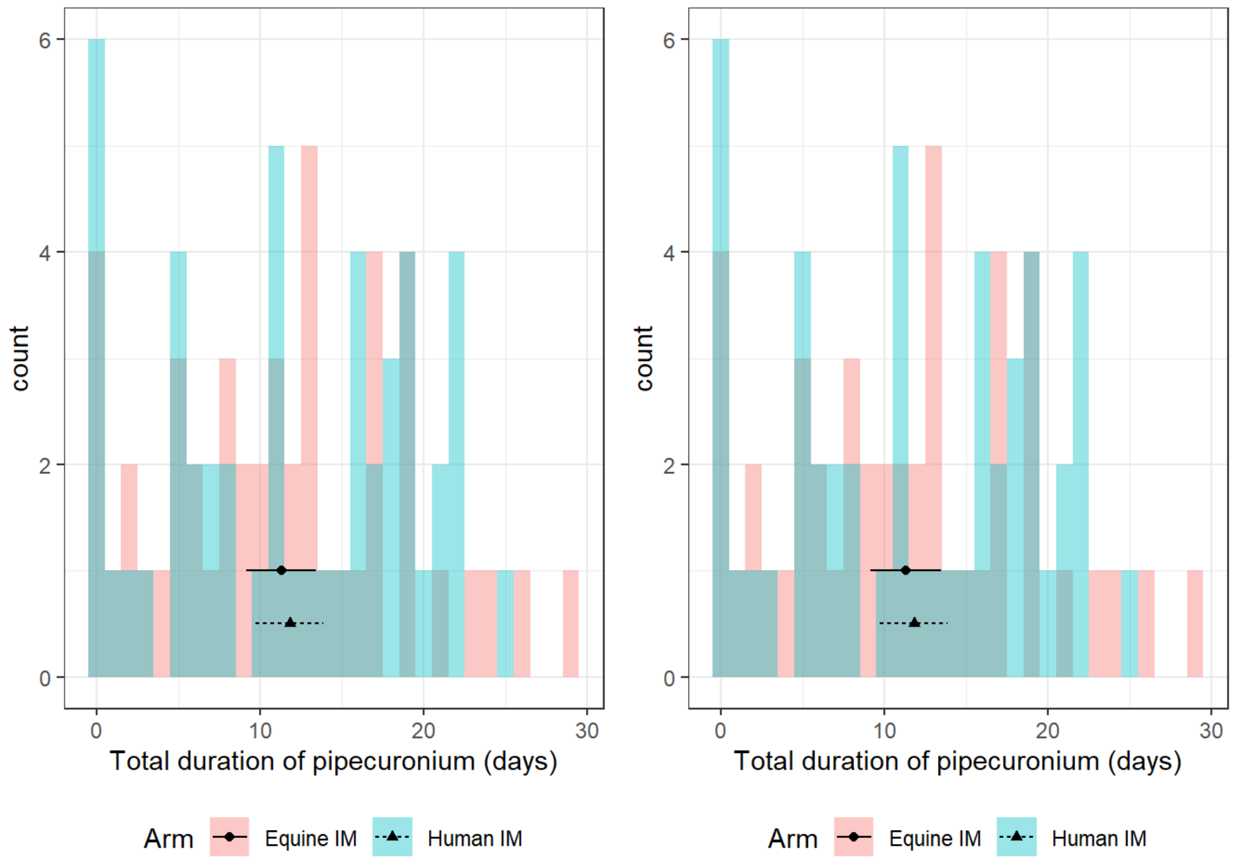

Mean values and 95% confidence intervals were shown in the central horizontal lines of the histograms for intervention arms

Figure S15c Total duration of pipecuronium: intramuscular (IM) intervention including population receiving antitoxin at previous hospital)

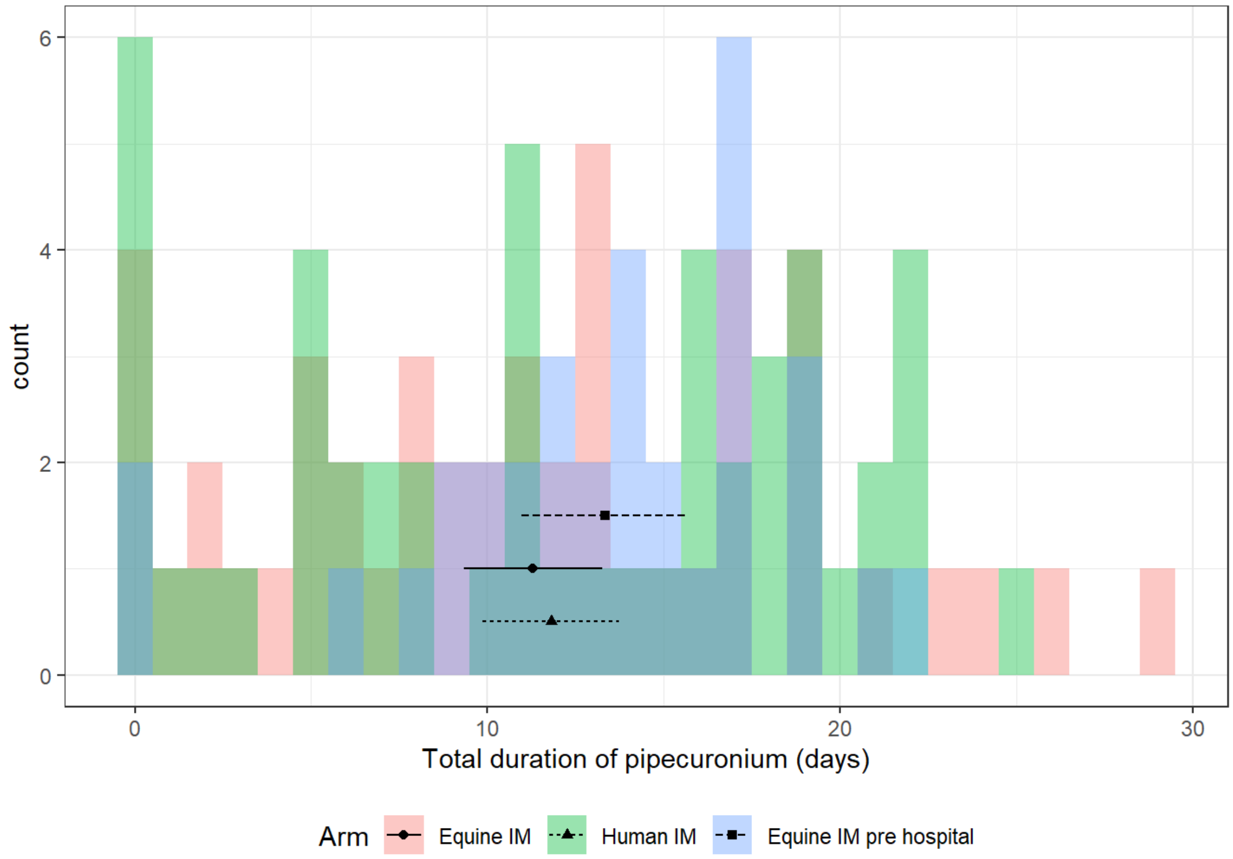

Mean values and 95% confidence intervals were shown in the central horizontal lines of the histograms for intervention arms

Antitoxin at previous hospital was intramuscular equine origin only.

Figures S16a-c Total dose of Diazepam during hospital stay

Figure S16a Total dose of diazepam intrathecal intervention intention-to-treat (left hand panel) and per-protocol (right hand panel) populations

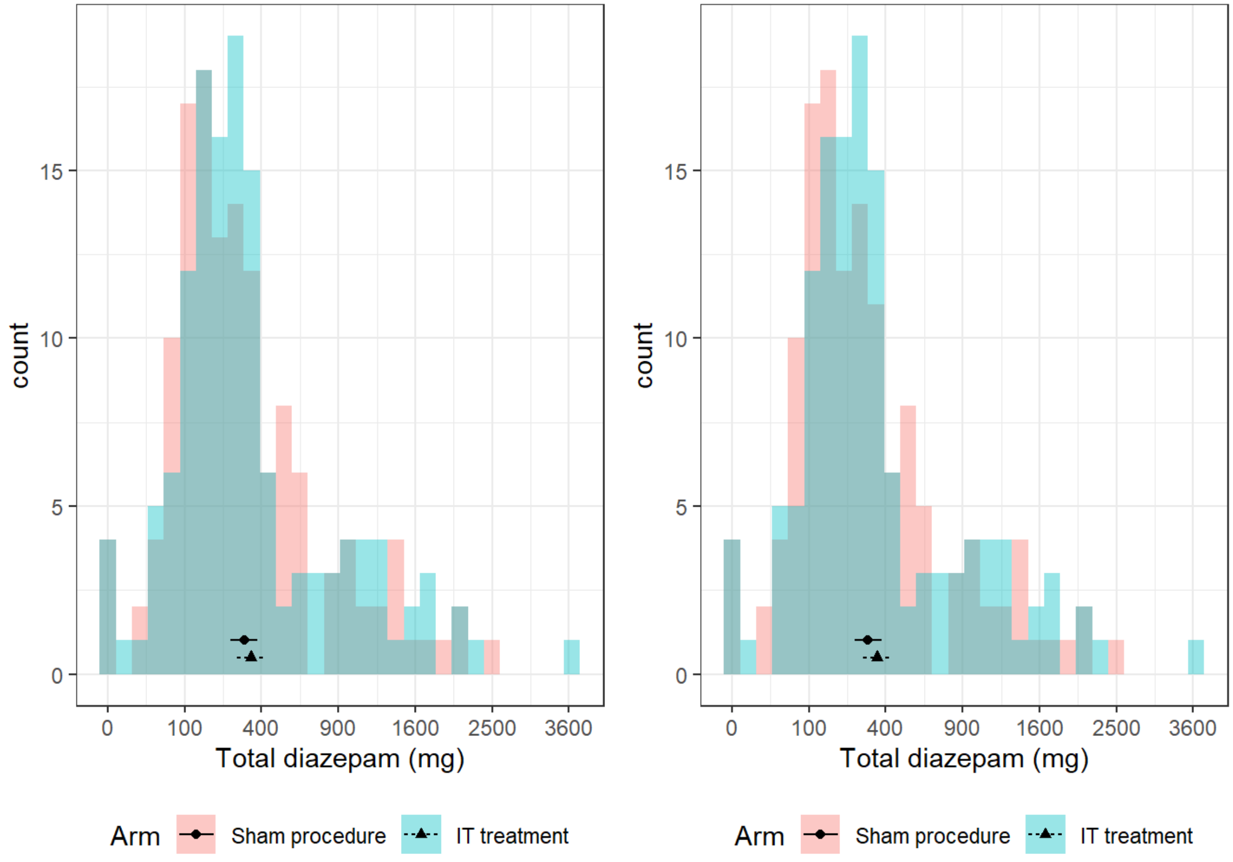

Mean values and 95% confidence intervals were shown in the central horizontal lines of the histograms for intervention arms

Figure S16b Total dose of diazepam intramuscular (IM) intervention intention-to-treat (left hand panel) and per-protocol (right hand panel) populations

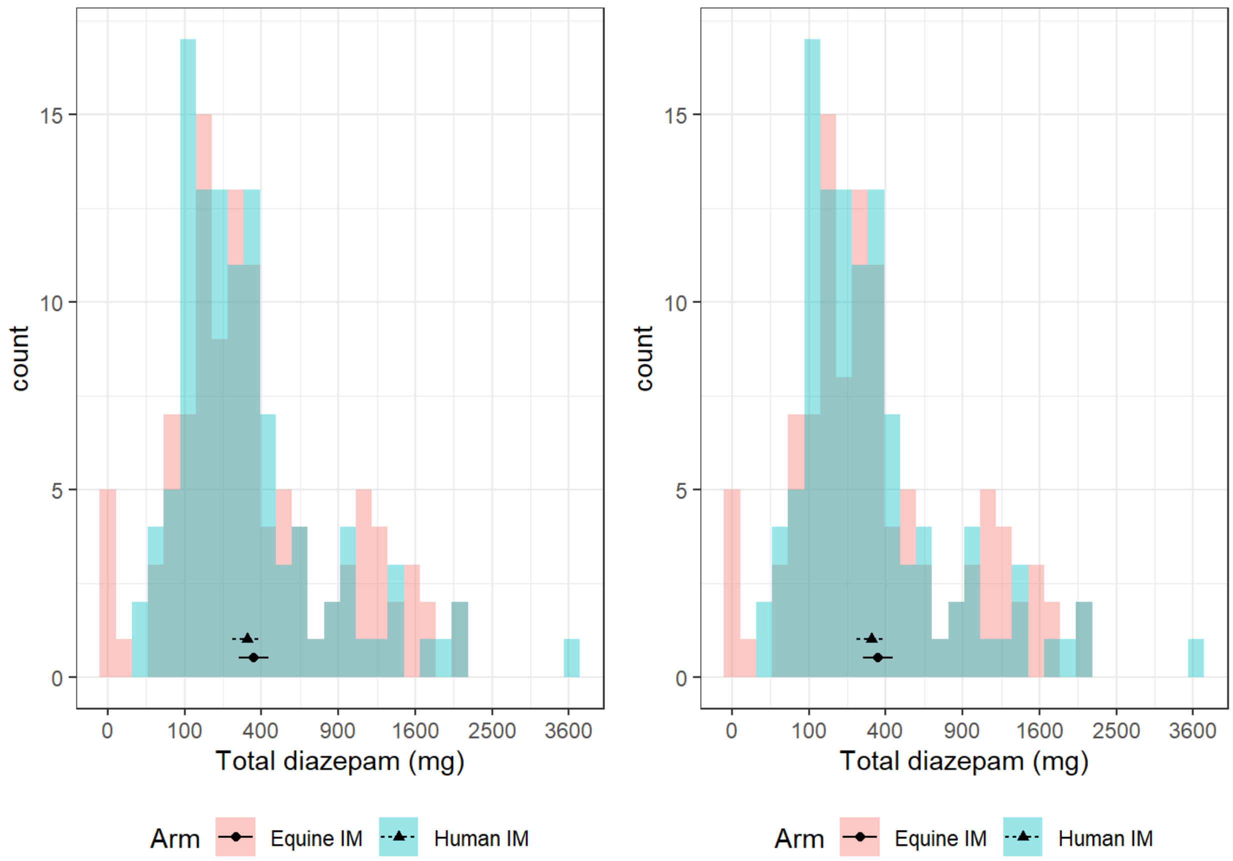

Mean values and 95% confidence intervals were shown in the central horizontal lines of the histograms for intervention arms

Figure S16c Total dose of diazepam (Intramuscular (IM) intervention including population receiving antitoxin at previous hospital)

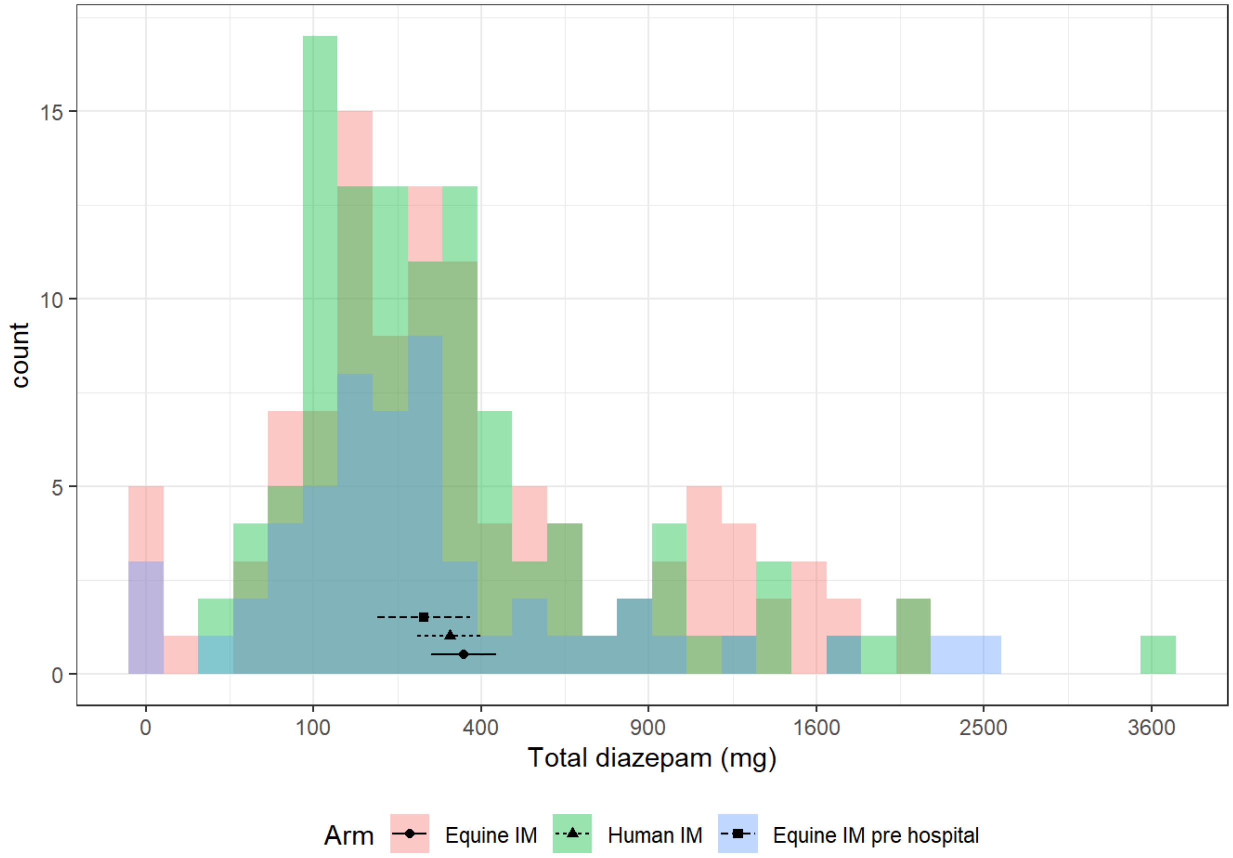

Mean values and 95% confidence intervals were shown in the central horizontal lines of the histograms for intervention arms

Antitoxin at previous hospital was intramuscular equine origin only.

Figures S17 a-c Total dose of midazolam during hospital stay

Figure S17a Total dose of midazolam intrathecal intervention intention-to-treat (left hand panel) and per-protocol (right hand panel) populations

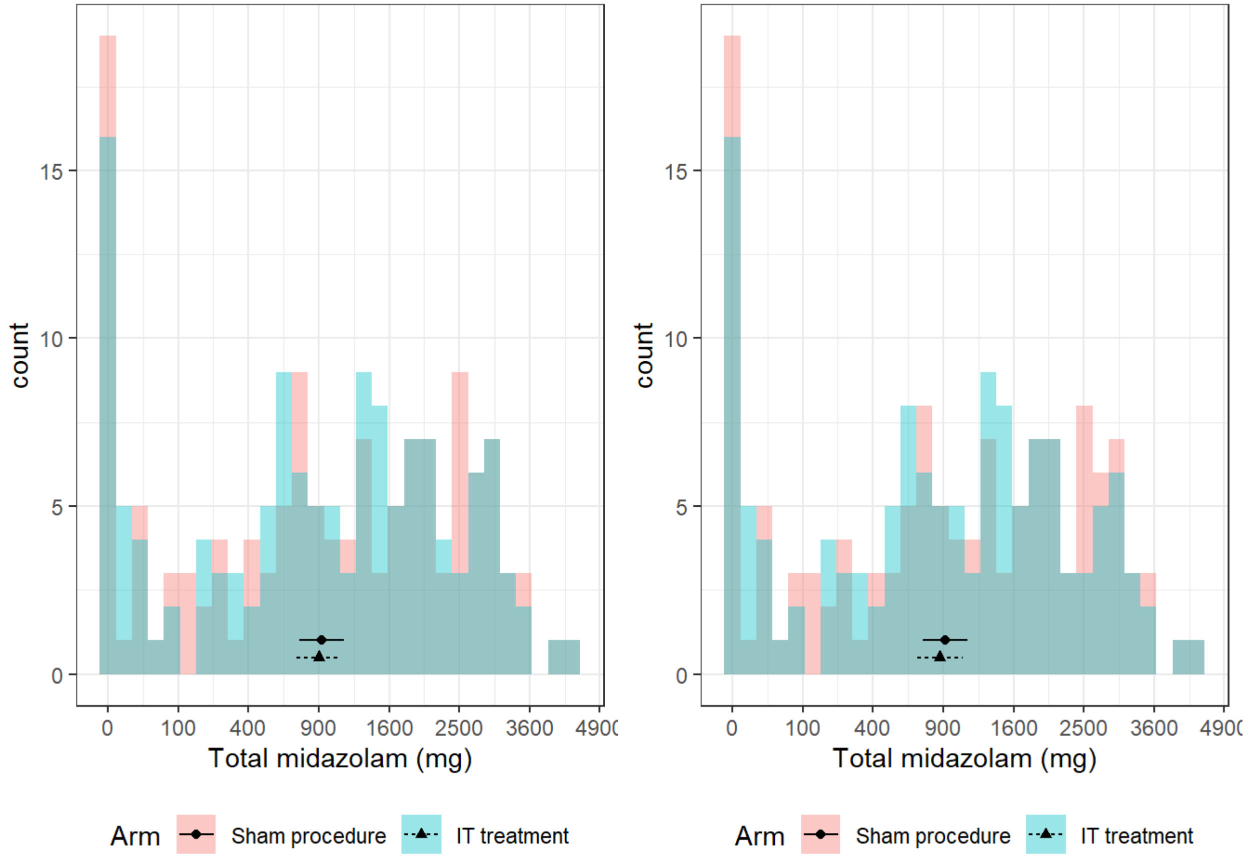

Mean values and 95% confidence intervals were shown in the central horizontal lines of the histograms for intervention arms

Figure S17b Total dose of midazolam intramuscular (IM) intervention intention-to-treat (left hand panel) and per-protocol (right hand panel) populations

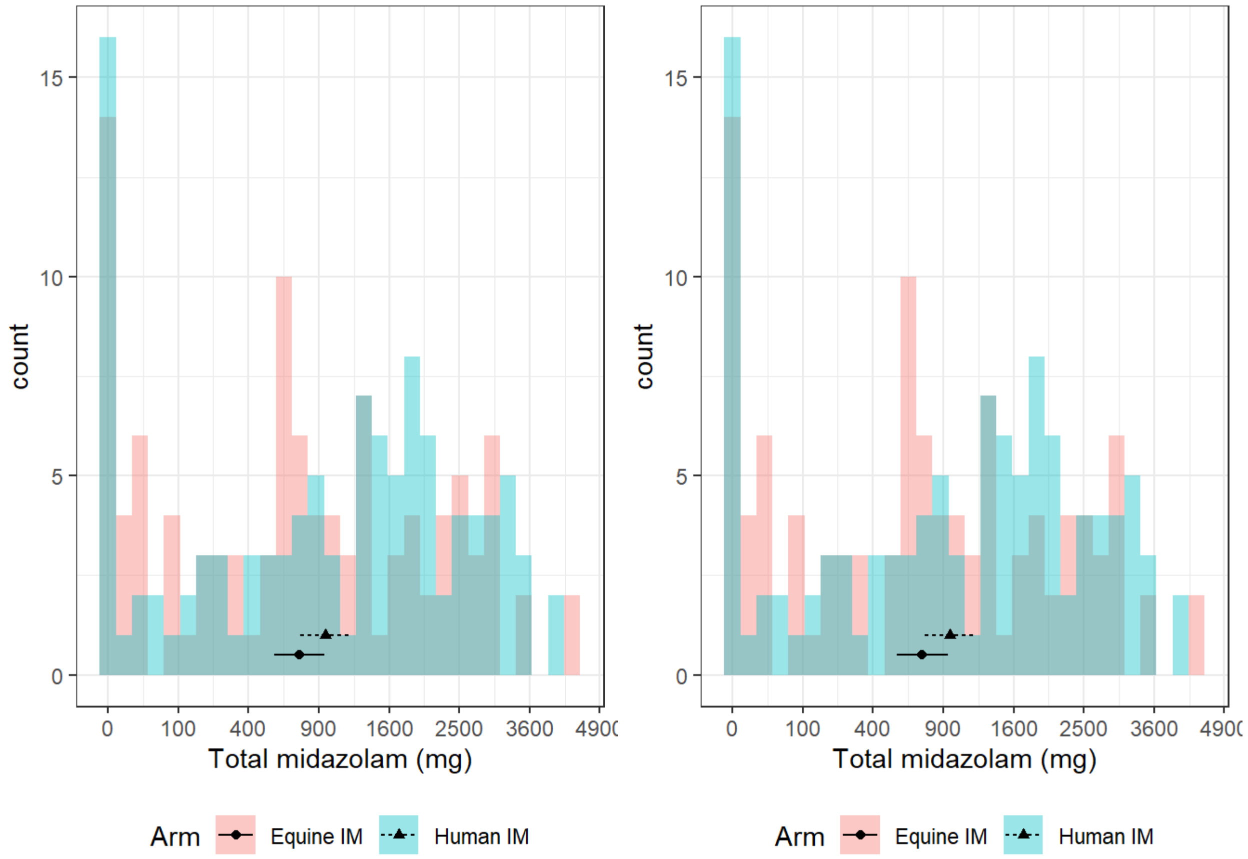

Mean values and 95% confidence intervals were shown in the central horizontal lines of the histograms for intervention arms

Figure S17c Total dose of midazolam (Intramuscular (IM) intervention including population receiving antitoxin at previous hospital)

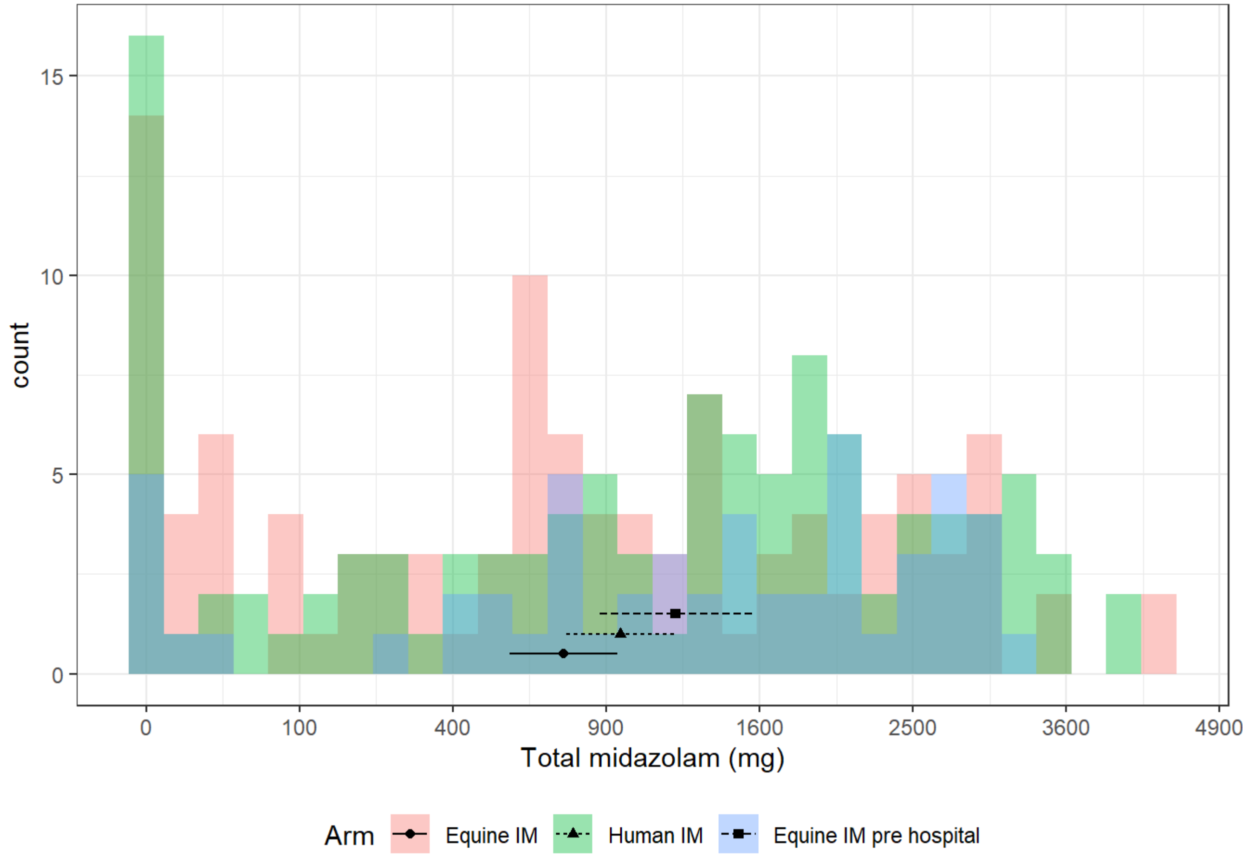

Mean values and 95% confidence intervals were shown in the central horizontal lines of the histograms for intervention arms

Antitoxin at previous hospital was intramuscular equine origin only.

Figures S18a-c Total dose of benzodiazepines during hospital stay

Figure S18a Total dose of benzodiazepines intrathecal intervention intention-to-treat (left hand panel) and per-protocol (right hand panel) populations

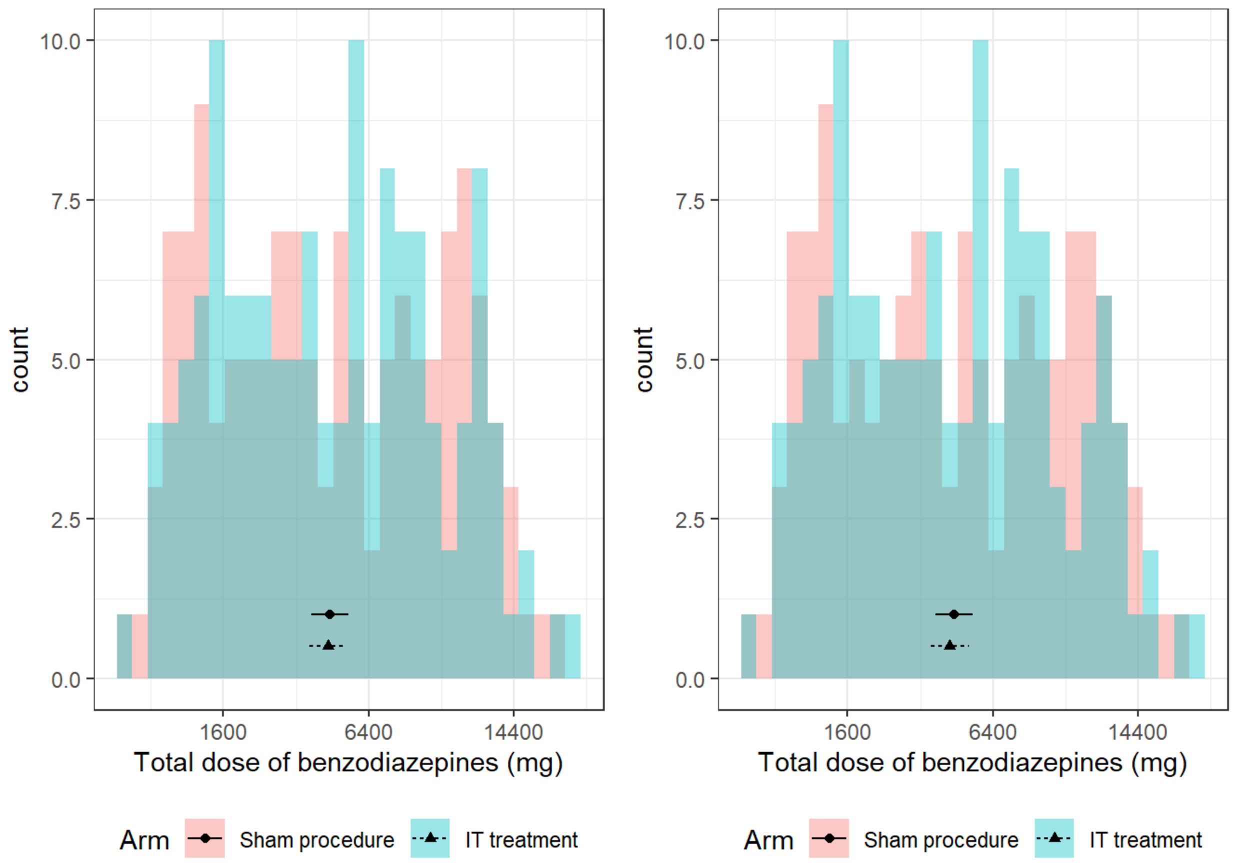

Mean values and 95% confidence intervals were shown in the central horizontal lines of the histograms for intervention arms

Figure S18b Total dose of benzodiazepines intramuscular (IM) intervention intention-to-treat (left hand panel) and per-protocol (right hand panel) populations

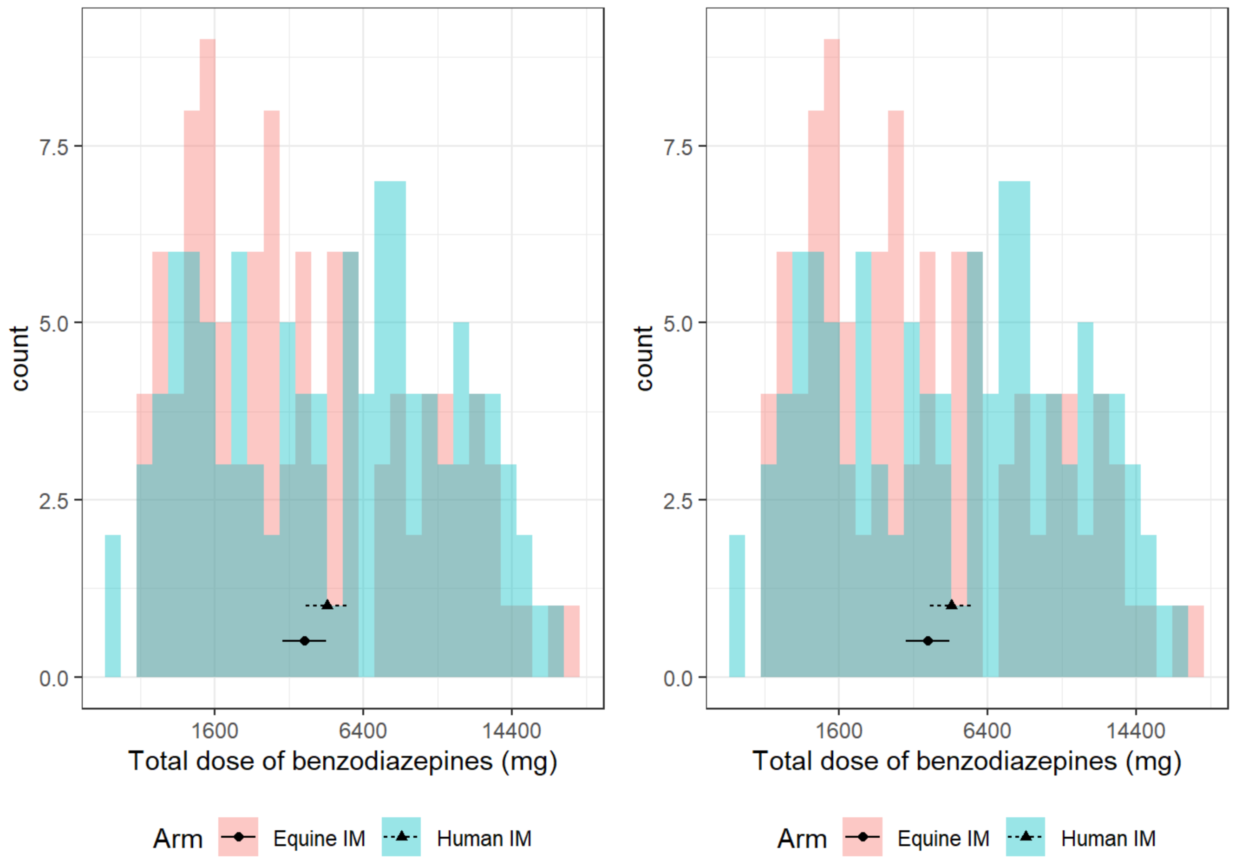

Mean values and 95% confidence intervals were shown in the central horizontal lines of the histograms for intervention arms

Figure S18c Total dose of benzodiazepines (Intramuscular (IM) intervention including population receiving antitoxin at previous hospital)

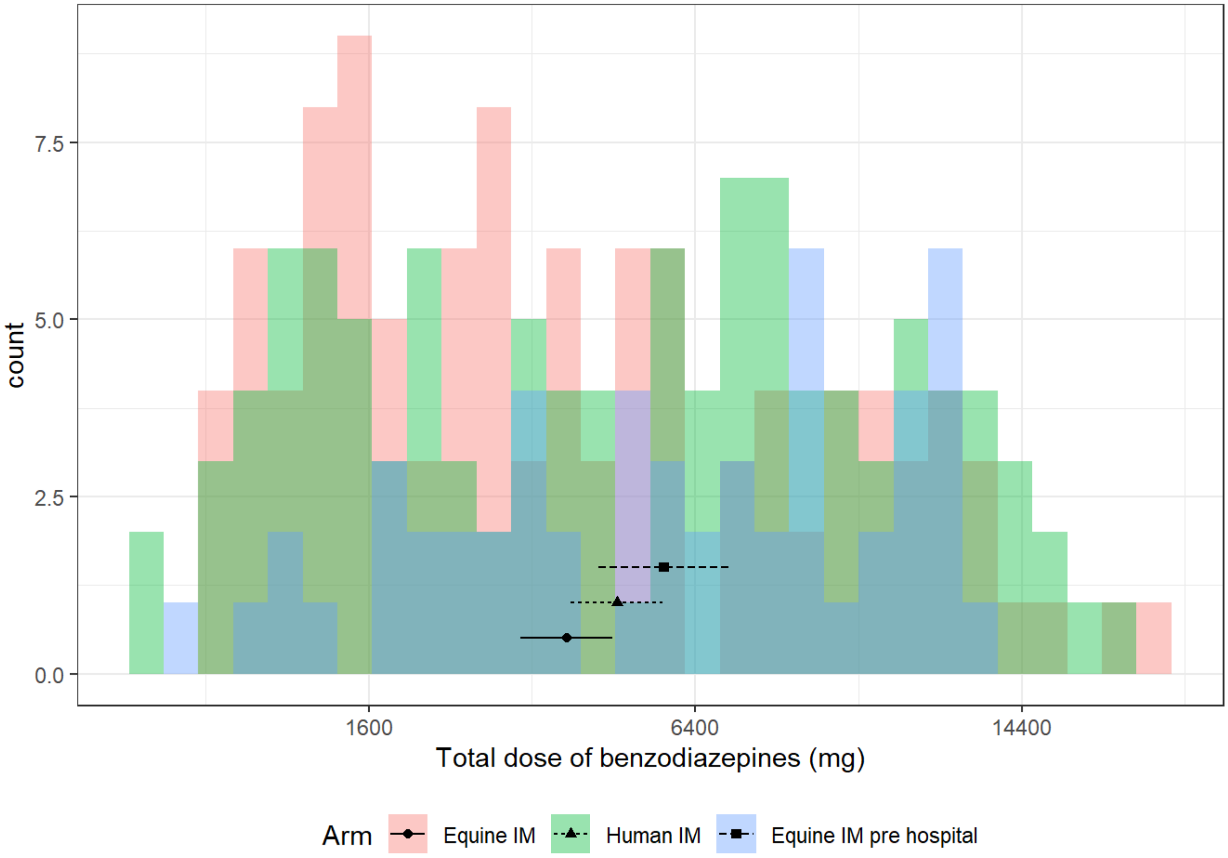

Mean values and 95% confidence intervals were shown in the central horizontal lines of the histograms for intervention arms

Antitoxin at previous hospital was intramuscular equine origin only.

Figures S19a-c Total duration of benzodiazepines

Figure S19a Total duration of benzodiazepines intrathecal intervention intention-to-treat (left hand panel) and per-protocol (right hand panel) populations

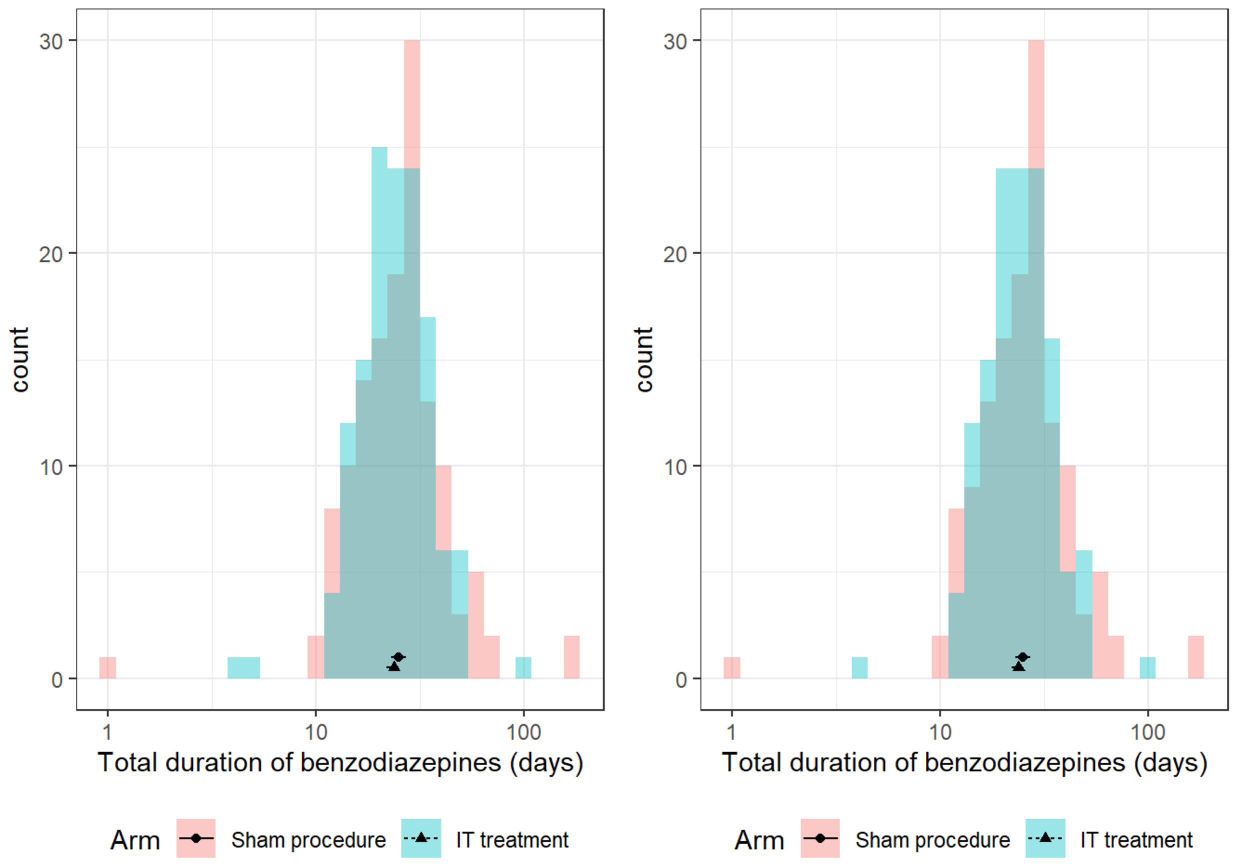

Mean values and 95% confidence intervals were shown in the central horizontal lines of the histograms for intervention arms

Figure S19b Total duration of benzodiazepines intramuscular (IM) intervention intention-to-treat (left hand panel) and per-protocol (right hand panel) populations

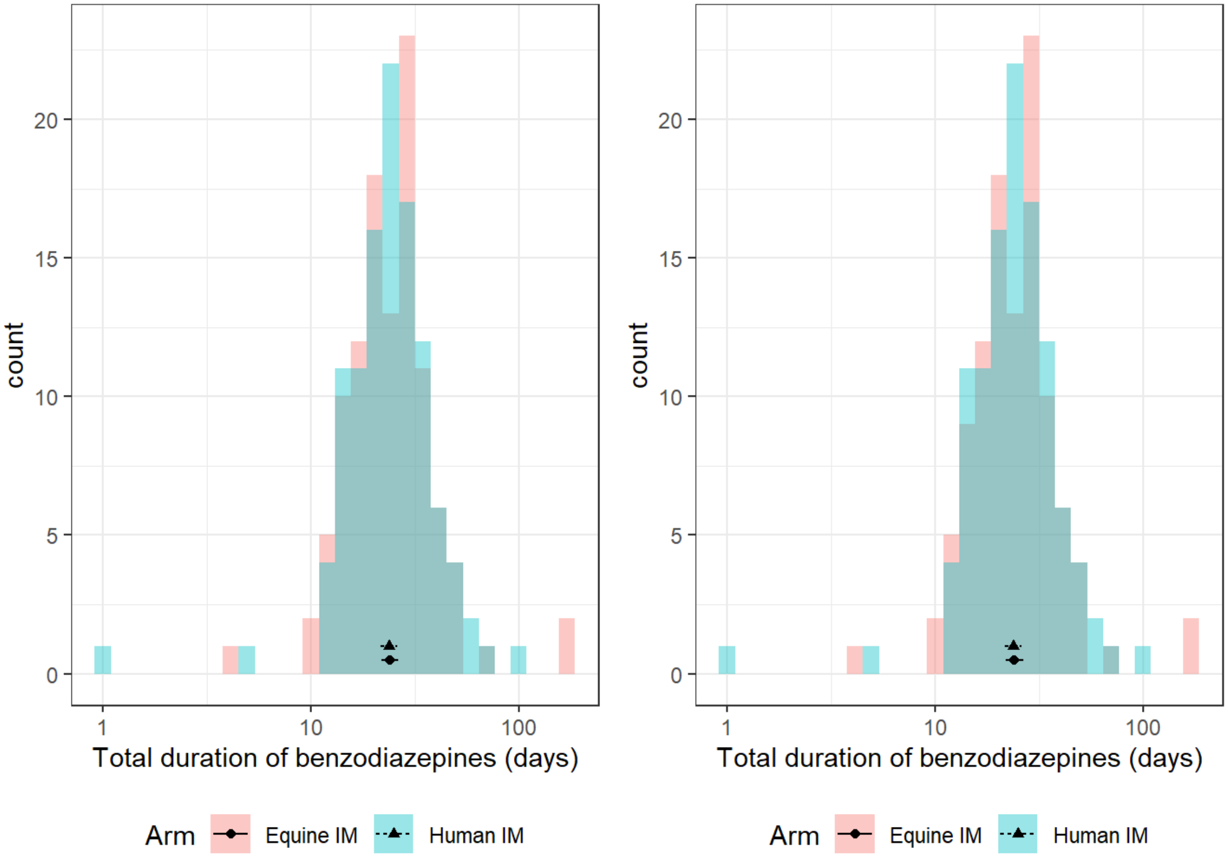

Mean values and 95% confidence intervals were shown in the central horizontal lines of the histograms for intervention arms

Figure S19c Total duration of benzodiazepines (Intramuscular (IM) intervention including population receiving antitoxin at previous hospital)

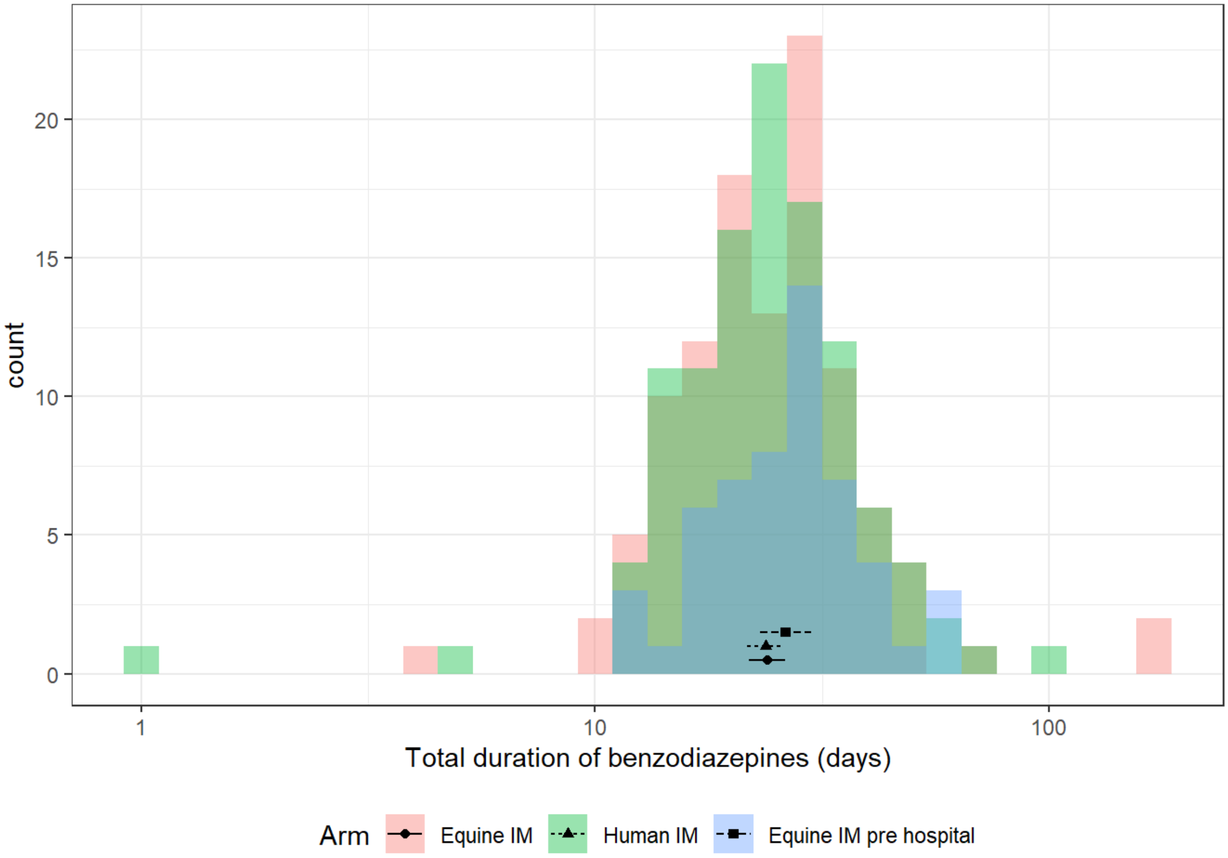

Mean values and 95% confidence intervals were shown in the central horizontal lines of the histograms for intervention arms

Antitoxin at previous hospital was intramuscular equine origin only.

Figures S20a-c Cost of ICU Stay

Figure S20a Cost of ICU stay intrathecal intervention intention-to-treat (left hand panel) and per-protocol (right hand panel) populations

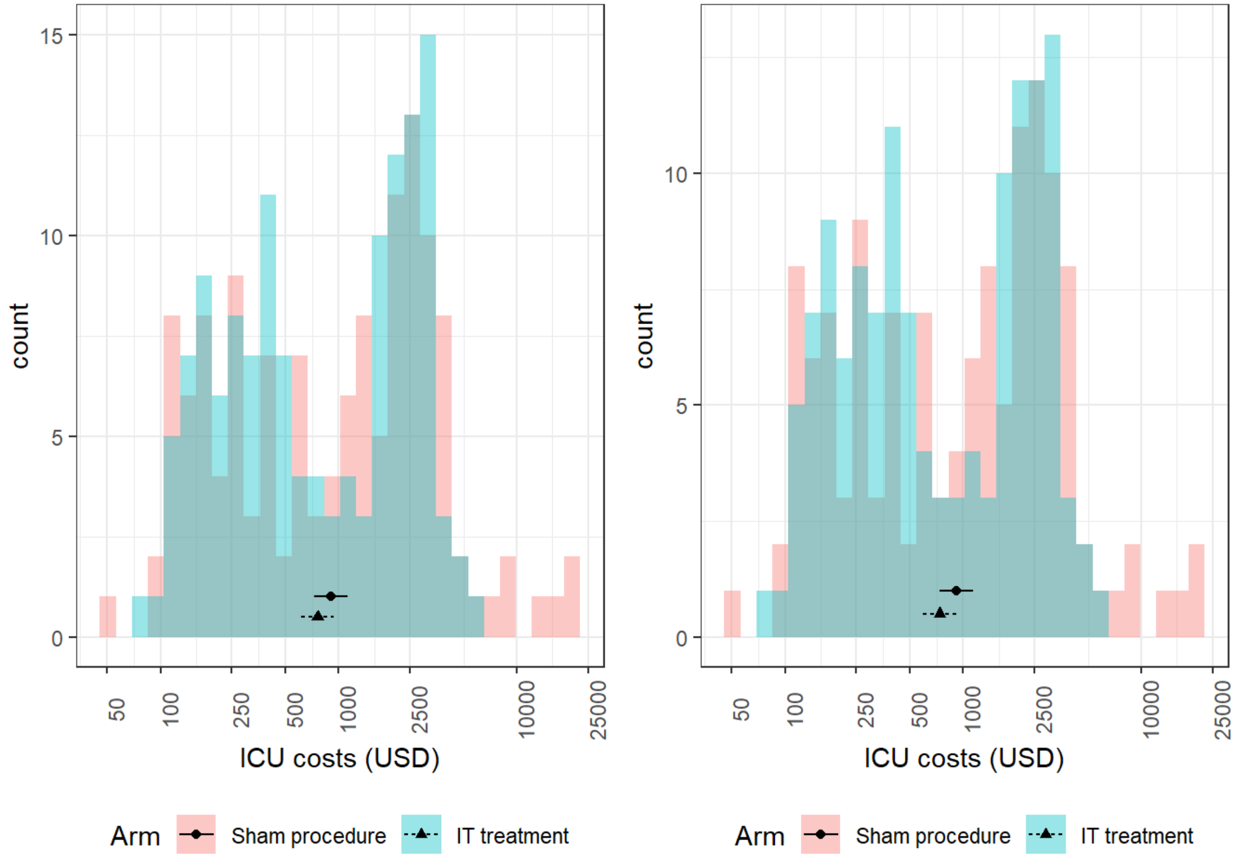

Mean values and 95% confidence intervals were shown in the central horizontal lines of the histograms for intervention arms

Figure S20b Cost of ICU stay intramuscular (IM)l intervention intention-to-treat (left hand panel) and per-protocol (right hand panel) populations

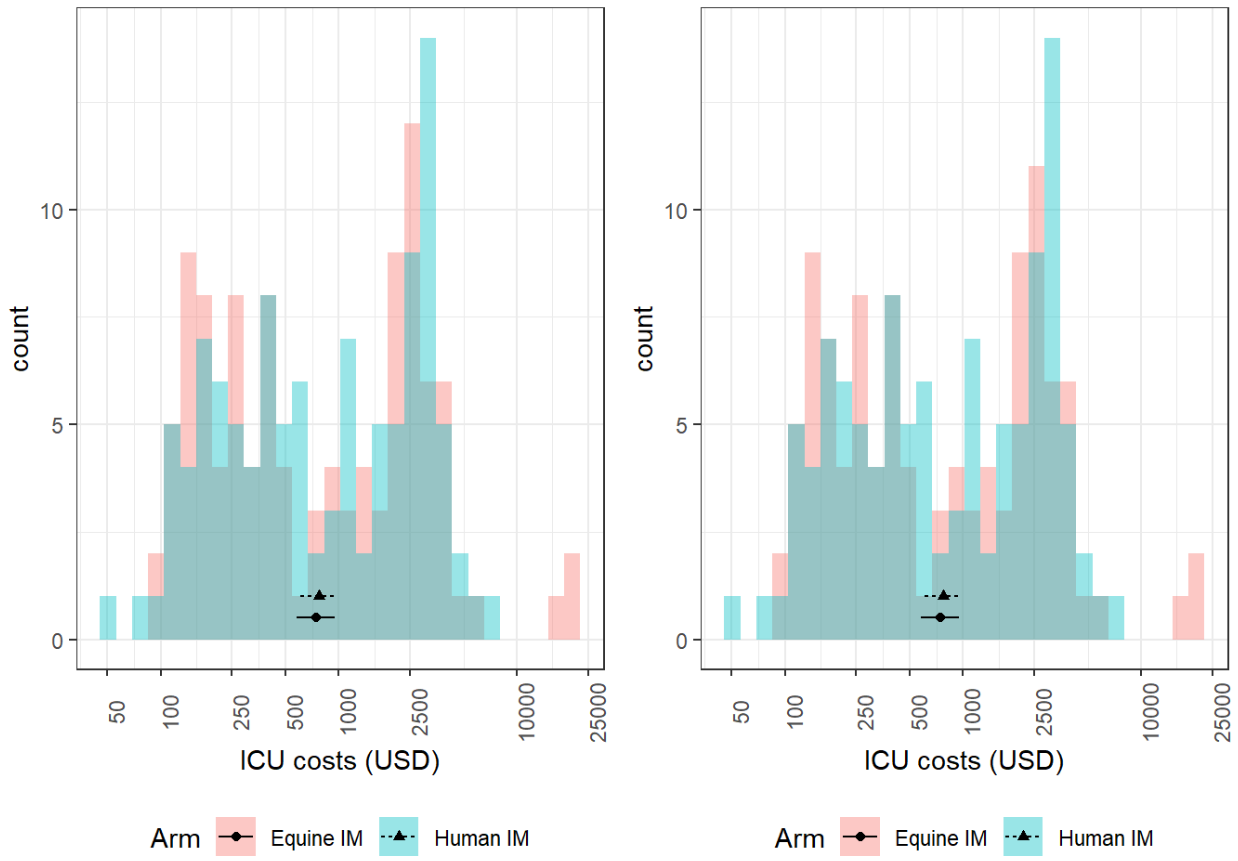

Mean values and 95% confidence intervals were shown in the central horizontal lines of the histograms for intervention arms

Figure S20c Cost of ICU stay (Intramuscular (IM) intervention including population receiving antitoxin at previous hospital)

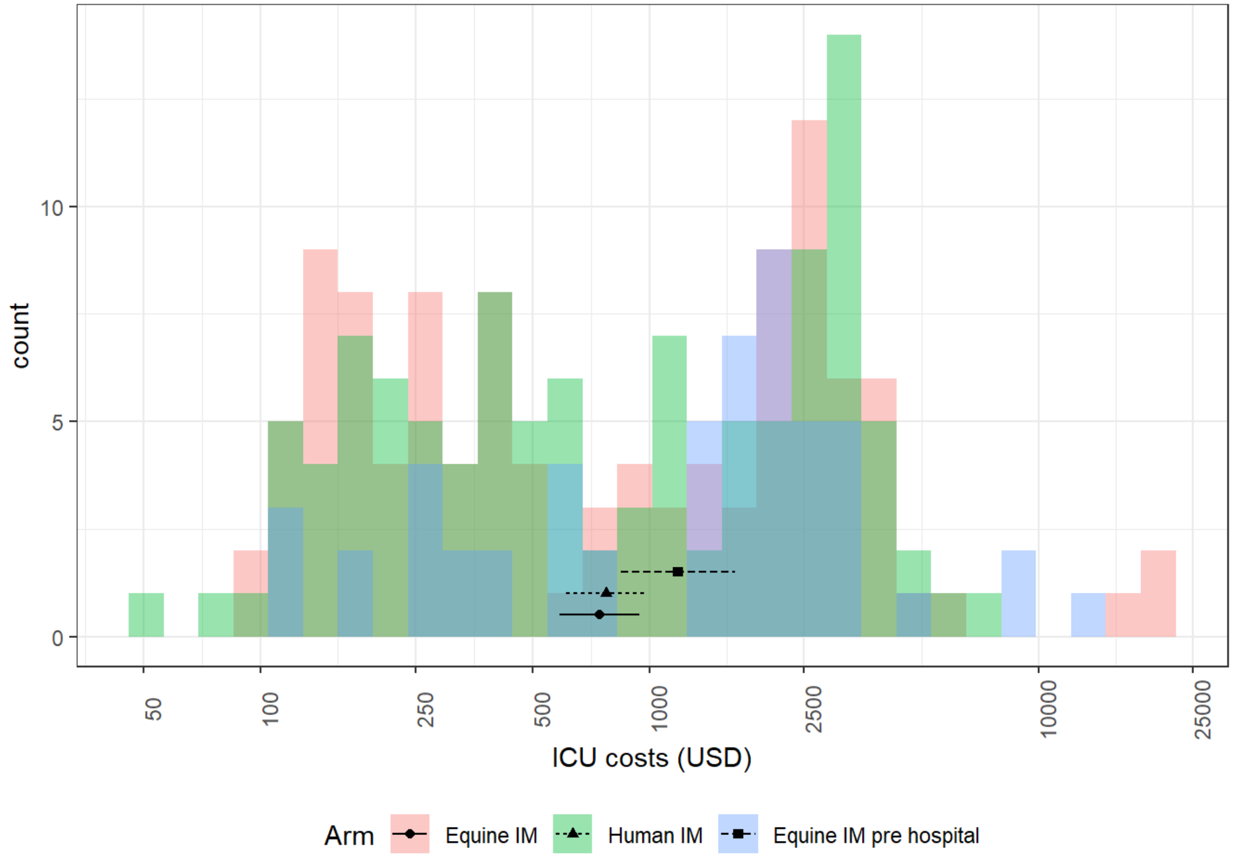

Mean values and 95% confidence intervals were shown in the central horizontal lines of the histograms for intervention arms

Antitoxin at previous hospital was intramuscular equine origin only.

Figures S21a-c Cost of hospital Stay

Figure S21a Cost of hospital stay intrathecal intervention intention-to-treat (left hand panel) and per-protocol (right hand panel) populations

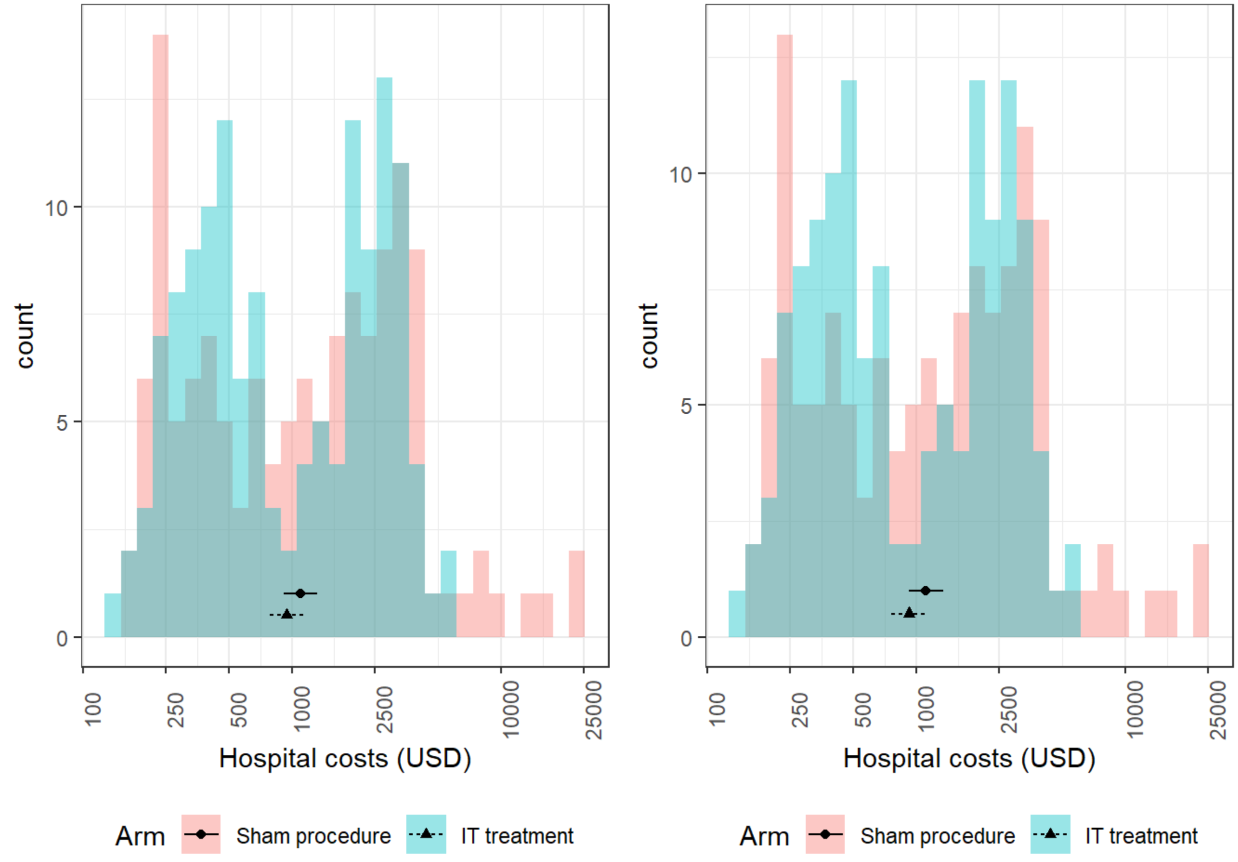

Mean values and 95% confidence intervals were shown in the central horizontal lines of the histograms for intervention arms

Figure S21b Cost of hospital stay intramuscular (IM) intervention intention-to-treat (left hand panel) and per-protocol (right hand panel) populations

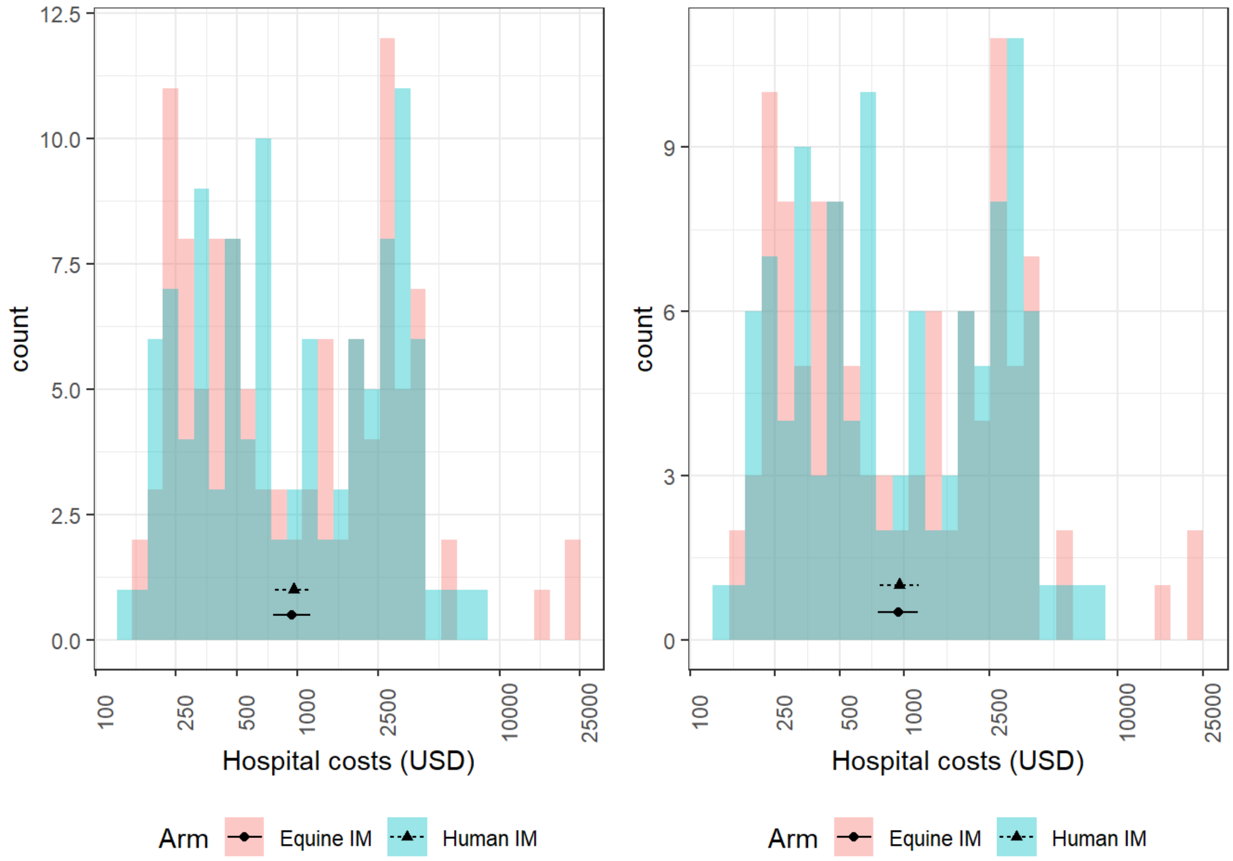

Mean values and 95% confidence intervals were shown in the central horizontal lines of the histograms for intervention arms

Figure S21c Cost of hospital stay (Intramuscular (IM) intervention including population receiving antitoxin at previous hospital)

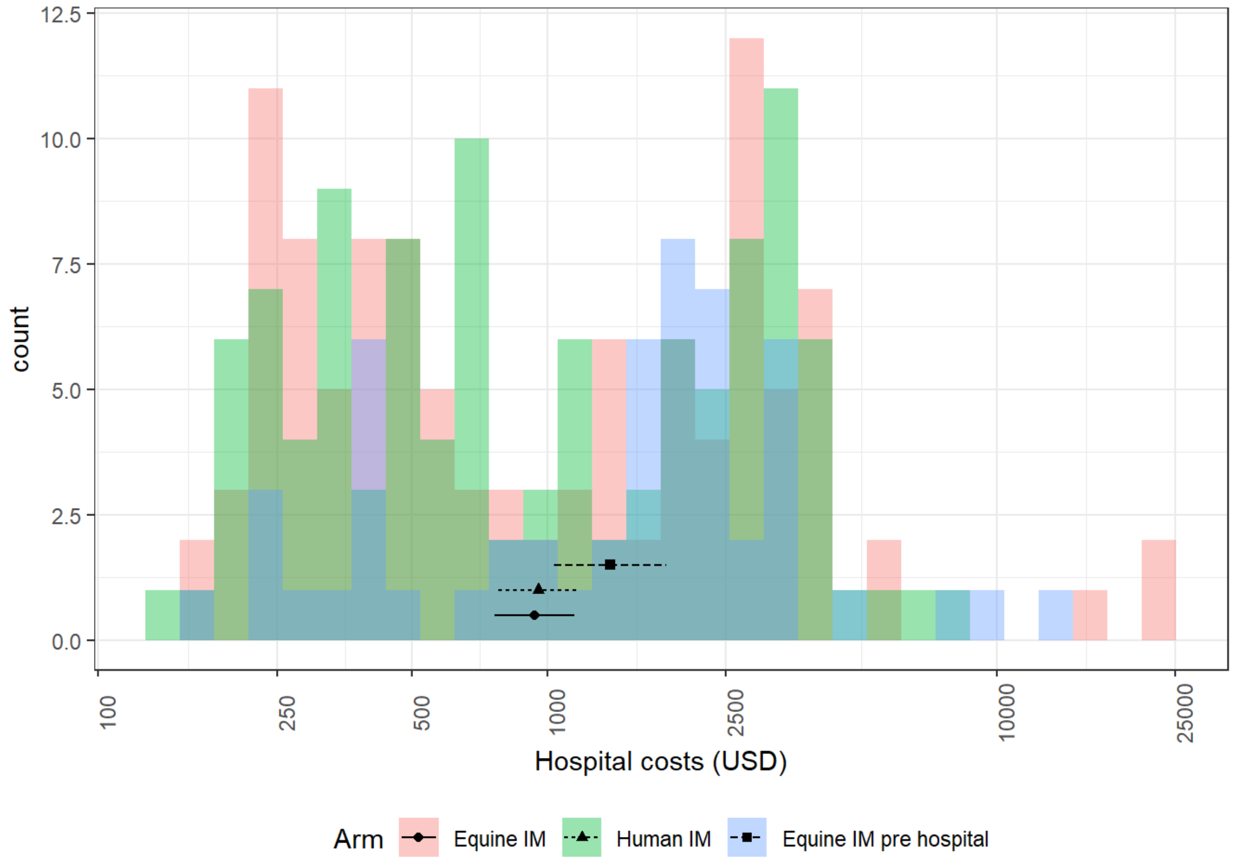

Mean values and 95% confidence intervals were shown in the central horizontal lines of the histograms for intervention arms

Antitoxin at previous hospital was intramuscular equine origin only.

Table S11 Frequency of adverse events classified as ‘possibly related’ or ‘related to’ interventions (intention –to-treat population)

		Sham procedure	Intrathecal antitoxin	Equine intramuscular antitoxin	Human intramuscular antitoxin
**Grade 1 & 2 events**	Nausea	0	1	1	0
	Headache	0	8	2	6
	Skin rash/reaction/ pruritus	9	7	8	8
	Fever/chills	0	6	2	4
	Other (n)	Back pain (1)<br>Deep vein thrombosis (1)	Atrial thrombosis (1)	Deep vein thrombosis (1)	Back pain (1)<br>Atrial thrombosis (1)
**Grade 3 & 4 events**	Thromboembolic events (n)	Deep vein thrombosis (2)	Deep vein thrombosis (1)<br>Pulmonary embolus (2)	Deep vein thrombosis (2)<br>Pulmonary Embolus (1)	Deep vein thrombosis (1)<br>Pulmonary embolus (1)
	Other (n)	Hypotension (1)<br>Cerebral infarction (1)	Urinary retention (1)	Hypotension (1)<br>Cerebral infarction (1)<br>Urinary retention (1)	

Table S12 Number of patients with adverse events (AE) (Intramuscular intervention per-protocol population)

	Equine intramuscular (N=106)		Human intramuscular (N=109)		
**AE Characteristic**	n	Summary statistic	n	Summary statistic	P-value
Grade I	106	18/106 (17%)	109	17/109 (16%)	0.783
Grade II	106	44/106 (42%)	109	40/109 (37%)	0.47
Grade III	106	16/106 (15%)	109	12/109 (11%)	0.374
Grade IV	106	5/106 (5%)	109	4/109 (4%)	0.701
Related	106	1/106 (1%)	109	2/109 (2%)	0.577
Possibly related*	106	13/106 (12%)	109	15/109 (14%)	0.744
Unrelated	106	59/106 (56%)	109	58/109 (53%)	0.718
Number AEs per patient	106		109		0.838
- 0		41/106 (39%)		41/109 (38%)	
- 1		18/106 (17%)		24/109 (22%)	
- 2		15/106 (14%)		17/109 (16%)	
- 3		9/106 (8%)		5/109 (5%)	
- 4		8/106 (8%)		10/109 (9%)	
- 5		6/106 (6%)		4/109 (4%)	
- >5		9/106 (8%)		8/109 (7%)	

Table S13 Number of patients with adverse events(AE) (Intrathecal intervention per-protocol population)

	Intrathecal treatment (N=132)		Sham procedure (N=132)		
**AE Characteristic**	n	Summary statistic	n	Summary statistic	P-value
Grade I	132	23/132 (17%)	132	22/132 (17%)	0.87
Grade II	132	52/132 (39%)	132	54/132 (41%)	0.802
Grade III	132	14/132 (11%)	132	23/132 (17%)	0.111
Grade IV	132	4/132 (3%)	132	5/132 (4%)	0.734
Related	132	2/132 (2%)	132	0/132 (0%)	0.156
Possibly related	132	17/132 (13%)	132	12/132 (9%)	0.325
Unrelated	132	69/132 (52%)	132	82/132 (62%)	0.106
Number AEs per patient	132		132		0.123
- 0		52/132 (39%)		47/132 (36%)	
- 1		26/132 (20%)		26/132 (20%)	
- 2		17/132 (13%)		20/132 (15%)	
- 3		6/132 (5%)		12/132 (9%)	
- 4		14/132 (11%)		9/132 (7%)	
- 5		11/132 (8%)		4/132 (3%)	
- >5		6/132 (5%)		14/132 (11%)	

Table S14 Number of patients with adverse events (AE) (Intramuscular intervention including population receiving antitoxin at previous hospital)

	Equine intramuscular (N=108)		Antitoxin at previous hospital [1] (N=54)		Human intramuscular (N=109)		
Characteristic	n	Summary statistic	n	Summary statistic	n	Summary statistic	P-value
Grade I	108	19/108 (18%)	54	12/54 (22%)	109	17/109 (16%)	0.58
Grade II	108	45/108 (42%)	54	25/54 (46%)	109	40/109 (37%)	0.48
Grade III	108	16/108 (15%)	54	12/54 (22%)	109	12/109 (11%)	0.165
Grade IV	108	5/108 (5%)	54	1/54 (2%)	109	4/109 (4%)	0.676
Related	108	2/108 (2%)	54	0/54 (0%)	109	2/109 (2%)	0.603
Possibly related	108	14/108 (13%)	54	4/54 (7%)	109	15/109 (14%)	0.48
Unrelated	108	61/108 (56%)	54	37/54 (69%)	109	58/109 (53%)	0.169
Number AEs per patient	108		54		109		0.643
- 0		41/108 (38%)		17/54 (31%)		41/109 (38%)	
- 1		18/108 (17%)		12/54 (22%)		24/109 (22%)	
- 2		16/108 (15%)		5/54 (9%)		17/109 (16%)	
- 3		10/108 (9%)		4/54 (7%)		5/109 (5%)	
- 4		8/108 (7%)		5/54 (9%)		10/109 (9%)	
- 5		6/108 (6%)		7/54 (13%)		4/109 (4%)	
- >5		9/108 (8%)		4/54 (7%)		8/109 (7%)	

[1] Antitoxin available at previous hospital was intramuscular equine origin only.

Table S15 All adverse events (Intramuscular intervention intention-to-treat population)

	Equine intramuscular (N=265)		Human intramuscular (N=228)		
Characteristic	n	Summary statistic	n	Summary statistic	P-value
Adverse Event	224		187		0.53
- Acute renal failure		2/224 (1%)		1/187 (1%)	
- Adrenal insufficiency		3/224 (1%)		0/187 (0%)	
- Allergy		1/224 (0%)		0/187 (0%)	
- Anemia		6/224 (3%)		2/187 (1%)	
- Arterial thromboembolism		1/224 (0%)		1/187 (1%)	
- Arthralgia		1/224 (0%)		3/187 (2%)	
- Arthritis		0/224 (0%)		3/187 (2%)	
- Aspiration pneumonia		5/224 (2%)		2/187 (1%)	
- Atrial fibrillation		2/224 (1%)		0/187 (0%)	
- Atrial thrombosis		0/224 (0%)		1/187 (1%)	
- Back pain		0/224 (0%)		1/187 (1%)	
- Blood stream infection		7/224 (3%)		5/187 (3%)	
- Bruising		1/224 (0%)		0/187 (0%)	
- Cardiac arrest		1/224 (0%)		1/187 (1%)	
- Cerebral infarction		1/224 (0%)		0/187 (0%)	
- Cerebral hemorrhage		0/224 (0%)		1/187 (1%)	
- Chills		0/224 (0%)		1/187 (1%)	
- Cholangitis		0/224 (0%)		1/187 (1%)	
- Chronic venous insufficiency		1/224 (0%)		0/187 (0%)	
- Conjunctivitis		3/224 (1%)		1/187 (1%)	
- Constipation		17/224 (8%)		16/187 (9%)	
- Creatinine increase		0/224 (0%)		1/187 (1%)	
- Deep vein thrombosis		2/224 (1%)		1/187 (1%)	
- Dizziness		1/224 (0%)		2/187 (1%)	
- Dysphagia		0/224 (0%)		1/187 (1%)	
- Facial nerve disorder		1/224 (0%)		0/187 (0%)	
- Failed extubation		1/224 (0%)		1/187 (1%)	
- Fever		1/224 (0%)		3/187 (2%)	
- Gastritis		4/224 (2%)		1/187 (1%)	
- Headache		2/224 (1%)		6/187 (3%)	
- Heat stroke		1/224 (0%)		0/187 (0%)	
- Hematuria		1/224 (0%)		2/187 (1%)	
- Hemorrhoids		1/224 (0%)		0/187 (0%)	
- Hiccup		0/224 (0%)		1/187 (1%)	
- Hospital acquired pneumonia		0/224 (0%)		1/187 (1%)	

	Equine intramuscular (N=265)		Human intramuscular (N=228)		
Characteristic	n	Summary statistic	n	Summary statistic	P-value
- Hypernatremia		1/224 (0%)		0/187 (0%)	
- Hyperpyrexia		0/224 (0%)		1/187 (1%)	
- Hypertension		0/224 (0%)		1/187 (1%)	
- Hypoalbuminemia		1/224 (0%)		0/187 (0%)	
- Hypocalcemia		12/224 (5%)		9/187 (5%)	
- Hypokalemia		19/224 (8%)		14/187 (7%)	
- Hypomagnesemia		4/224 (2%)		3/187 (2%)	
- Hyponatremia		8/224 (4%)		9/187 (5%)	
- Hypotension		5/224 (2%)		1/187 (1%)	
- Insomnia		1/224 (0%)		0/187 (0%)	
- Low weight, muscle wasting		3/224 (1%)		0/187 (0%)	
- Myalgia		0/224 (0%)		1/187 (1%)	
- Myocardial ischaemia		2/224 (1%)		1/187 (1%)	
- Myocarditis		0/224 (0%)		1/187 (1%)	
- Nausea		1/224 (0%)		0/187 (0%)	
- Neuralgia		1/224 (0%)		0/187 (0%)	
- Nose bleed		3/224 (1%)		3/187 (2%)	
- Oral infection		2/224 (1%)		2/187 (1%)	
- Otitis		1/224 (0%)		2/187 (1%)	
- Phlebitis		3/224 (1%)		3/187 (2%)	
- Platelet count decreased		2/224 (1%)		0/187 (0%)	
- Pneumothorax		1/224 (0%)		0/187 (0%)	
- Postoperative hemorrage		1/224 (0%)		0/187 (0%)	
- Pressure ulcer		2/224 (1%)		1/187 (1%)	
- Pruritus		0/224 (0%)		1/187 (1%)	
- Pyloric stenosis		0/224 (0%)		1/187 (1%)	
- Recurrent tetanus		1/224 (0%)		0/187 (0%)	
- Sinus bradycardia		1/224 (0%)		1/187 (1%)	
- Sinusitis		1/224 (0%)		0/187 (0%)	
- Skin allergy		7/224 (3%)		6/187 (3%)	
- Spinal disc herniation		1/224 (0%)		0/187 (0%)	
- Subcutaneous emphysema		0/224 (0%)		1/187 (1%)	
- Torticolis		1/224 (0%)		0/187 (0%)	
- Tracheal hemorrhage		8/224 (4%)		7/187 (4%)	
- Upper gastrointestinal hemorrhage		7/224 (3%)		10/187 (5%)	
- Urinary retention		1/224 (0%)		2/187 (1%)	
- Urinary tract infection		23/224 (10%)		18/187 (10%)	

Characteristic	Equine intramuscular (N=265) n	Summary statistic	Human intramuscular (N=228) n	Summary statistic	P-value
- Ventilator associated pneumonia		29/224 (13%)		27/187 (14%)	
- Viral infection		2/224 (1%)		0/187 (0%)	
- Voice alteration		1/224 (0%)		0/187 (0%)	
- Wound bleed		0/224 (0%)		1/187 (1%)	
- Wound infection		1/224 (0%)		0/187 (0%)	

Table S16 All adverse events (Intramuscular intervention per-protocol population)

	Equine intramuscular (N=260)		Human intramuscular (N=228)		
**Characteristic**	n	Summary statistic	n	Summary statistic	P-value
Adverse Event	219		187		0.60
- Acute renal failure		2/219 (1%)		1/187 (1%)	
- Adrenal insufficiency		3/219 (1%)		0/187 (0%)	
- Allergy		1/219 (0%)		0/187 (0%)	
- Anemia		6/219 (3%)		2/187 (1%)	
- Arterial thromboembolism		1/219 (0%)		1/187 (1%)	
- Arthralgia		1/219 (0%)		3/187 (2%)	
- Arthritis		0/219 (0%)		3/187 (2%)	
- Aspiration pneumonia		5/219 (2%)		2/187 (1%)	
- Atrial fibrillation*		2/219 (1%)		0/187 (0%)	
- Atrial thrombosis		0/219 (0%)		1/187 (1%)	
- Back pain		0/219 (0%)		1/187 (1%)	
- Blood stream infection		7/219 (3%)		5/187 (3%)	
- Bruising		1/219 (0%)		0/187 (0%)	
- Cardiac arrest		1/219 (0%)		1/187 (1%)	
- Cerebral infarction		1/219 (0%)		0/187 (0%)	
- Cerebral hemorrhage		0/219 (0%)		1/187 (1%)	
- Chills		0/219 (0%)		1/187 (1%)	
- Cholangitis		0/219 (0%)		1/187 (1%)	
- Chronic venous insufficiency		1/219 (0%)		0/187 (0%)	
- Conjunctivitis		3/219 (1%)		1/187 (1%)	
- Constipation		17/219 (8%)		16/187 (9%)	
- Creatinine increase		0/219 (0%)		1/187 (1%)	
- Deep vein thrombosis		2/219 (1%)		1/187 (1%)	
- Dizziness		1/219 (0%)		2/187 (1%)	
- Dysphagia		0/219 (0%)		1/187 (1%)	
- Failed extubation		1/219 (0%)		1/187 (1%)	
- Fever		1/219 (0%)		3/187 (2%)	
- Gastritis		4/219 (2%)		1/187 (1%)	
- Headache		2/219 (1%)		6/187 (3%)	
- Heat stroke		1/219 (0%)		0/187 (0%)	
- Hematuria		1/219 (0%)		2/187 (1%)	
- Hemorrhoids		1/219 (0%)		0/187 (0%)	
- Hiccup		0/219 (0%)		1/187 (1%)	
- Hospital acquired pneumonia		0/219 (0%)		1/187 (1%)	

	Equine intramuscular (N=260)		Human intramuscular (N=228)		
Characteristic	n	Summary statistic	n	Summary statistic	P-value
- Hypernatremia		1/219 (0%)		0/187 (0%)	
- Hyperpyrexia		0/219 (0%)		1/187 (1%)	
- Hypertension		0/219 (0%)		1/187 (1%)	
- Hypoalbuminemia		1/219 (0%)		0/187 (0%)	
- Hypocalcemia		12/219 (5%)		9/187 (5%)	
- Hypokalemia		19/219 (9%)		14/187 (7%)	
- Hypomagnesemia		4/219 (2%)		3/187 (2%)	
- Hyponatremia		8/219 (4%)		9/187 (5%)	
- Hypotension		5/219 (2%)		1/187 (1%)	
- Insomnia		1/219 (0%)		0/187 (0%)	
- Low weight, muscle wasting		3/219 (1%)		0/187 (0%)	
- Myalgia		0/219 (0%)		1/187 (1%)	
- Myocardial ischaemia		2/219 (1%)		1/187 (1%)	
- Myocarditis		0/219 (0%)		1/187 (1%)	
- Nausea		1/219 (0%)		0/187 (0%)	
- Neuralgia		1/219 (0%)		0/187 (0%)	
- Nose bleed		3/219 (1%)		3/187 (2%)	
- Oral infection		2/219 (1%)		2/187 (1%)	
- Otitis		1/219 (0%)		2/187 (1%)	
- Phlebitis		3/219 (1%)		3/187 (2%)	
- Platelet count decreased		2/219 (1%)		0/187 (0%)	
- Pneumothorax		1/219 (0%)		0/187 (0%)	
- Postoperative hemorrage		1/219 (0%)		0/187 (0%)	
- Pressure ulcer		2/219 (1%)		1/187 (1%)	
- Pruritus		0/219 (0%)		1/187 (1%)	
- Pyloric stenosis		0/219 (0%)		1/187 (1%)	
- Recurrent tetanus		1/219 (0%)		0/187 (0%)	
- Sinus bradycardia		1/219 (0%)		1/187 (1%)	
- Sinusitis		1/219 (0%)		0/187 (0%)	
- Skin allergy		5/219 (2%)		6/187 (3%)	
- Spinal disc herniation		1/219 (0%)		0/187 (0%)	
- Subcutaneous emphysema		0/219 (0%)		1/187 (1%)	
- Torticolis		1/219 (0%)		0/187 (0%)	
- Tracheal hemorrhage		8/219 (4%)		7/187 (4%)	
- Upper gastrointestinal hemorrhage		7/219 (3%)		10/187 (5%)	
- Urinary retention		1/219 (0%)		2/187 (1%)	
- UTI		22/219 (10%)		18/187 (10%)	

	Equine intramuscular (N=260)		Human intramuscular (N=228)		
**Characteristic**	n	Summary statistic	n	Summary statistic	P-value
- Ventilator associated pneumonia		29/219 (13%)		27/187 (14%)	
- Viral infection		2/219 (1%)		0/187 (0%)	
- Wound bleed		0/219 (0%)		1/187 (1%)	
- Wound infection		1/219 (0%)		0/187 (0%)	

Table S17 All adverse events (Intrathecal intervention intention-to-treat population)

	Intrathecal treatment (N=303)		Sham procedure (N=330)		
Characteristic	n	Summary statistic	n	Summary statistic	P-value
Adverse Event	251		283		0.37
- Acute renal failure		1/251 (0%)		3/283 (1%)	
- Adrenal insufficiency		1/251 (0%)		2/283 (1%)	
- Allergy		1/251 (0%)		0/283 (0%)	
- Anemia		2/251 (1%)		9/283 (3%)	
- Appendicitis		0/251 (0%)		1/283 (0%)	
- Arterial thromboembolism		2/251 (1%)		0/283 (0%)	
- Arthralgia		2/251 (1%)		2/283 (1%)	
- Arthritis		4/251 (2%)		1/283 (0%)	
- Aspiration pneumonia*		2/251 (1%)		6/283 (2%)	
- Atrial fibrillation		0/251 (0%)		2/283 (1%)	
- Atrial thrombosis		1/251 (0%)		0/283 (0%)	
- Back pain		1/251 (0%)		1/283 (0%)	
- Blood stream infection		9/251 (4%)		8/283 (3%)	
- Bruising		1/251 (0%)		0/283 (0%)	
- Cardiac arrest		0/251 (0%)		3/283 (1%)	
- Cellulitis		1/251 (0%)		0/283 (0%)	
- Cerbral infarction		0/251 (0%)		1/283 (0%)	
- Cerebral hemorrhage		1/251 (0%)		0/283 (0%)	
- Chills		1/251 (0%)		0/283 (0%)	
- Cholangitis		0/251 (0%)		1/283 (0%)	
- Chronic venous insufficiency		1/251 (0%)		0/283 (0%)	
- Conjunctivitis		2/251 (1%)		3/283 (1%)	
- Constipation		19/251 (8%)		19/283 (7%)	
- Creatinine increase		1/251 (0%)		0/283 (0%)	
- Deep vein thrombosis		1/251 (0%)		3/283 (1%)	
- Dizziness		3/251 (1%)		1/283 (0%)	
- Dysphagia		1/251 (0%)		0/283 (0%)	
- facial nerve disorder		0/251 (0%)		1/283 (0%)	
- Failed extubation		2/251 (1%)		0/283 (0%)	
- Fever		5/251 (2%)		0/283 (0%)	
- Gastritis		4/251 (2%)		1/283 (0%)	
- Gastrointestinal disorder		0/251 (0%)		1/283 (0%)	
- Headache		8/251 (3%)		0/283 (0%)	
- Heat stroke		0/251 (0%)		1/283 (0%)	
- Hematuria		1/251 (0%)		2/283 (1%)	
- Hemorrhoids		2/251 (1%)		1/283 (0%)	
- Hiccup		1/251 (0%)		0/283 (0%)	
- Hospital acquired pneumonia		2/251 (1%)		2/283 (1%)	
- Hyperglycaemia		1/251 (0%)		0/283 (0%)	
- Hypernatremia		0/251 (0%)		2/283 (1%)	
- Hyperpyrexia		1/251 (0%)		0/283 (0%)	
- Hypertension		0/251 (0%)		1/283 (0%)	
- Hypoalbuminemia		0/251 (0%)		1/283 (0%)	
- Hypocalcemia		15/251 (6%)		17/283 (6%)	
- Hypokalemia		21/251 (8%)		30/283 (11%)	

- Hypomagnesemia	4/251 (2%)	4/283 (1%)
- Hyponatremia	14/251 (6%)	13/283 (5%)
- Hypotension	1/251 (0%)	6/283 (2%)
- Insomnia	1/251 (0%)	0/283 (0%)
- Low weight, muscle wasting	1/251 (0%)	2/283 (1%)
- Myalgia	0/251 (0%)	1/283 (0%)
- Myocardial ischaemia	2/251 (1%)	1/283 (0%)
- Myocarditis	1/251 (0%)	1/283 (0%)
- Nausea	1/251 (0%)	0/283 (0%)
- Necrotic wound	1/251 (0%)	0/283 (0%)
- Neuralgia	0/251 (0%)	1/283 (0%)
- Nose bleed	5/251 (2%)	2/283 (1%)
- Oral infection	3/251 (1%)	1/283 (0%)
- Otitis	1/251 (0%)	2/283 (1%)
- pharyngitis	1/251 (0%)	0/283 (0%)
- Phlebitis	2/251 (1%)	6/283 (2%)
- Platelet count decreased	0/251 (0%)	2/283 (1%)
- Pleural abscess	0/251 (0%)	1/283 (0%)
- Pneumothorax	0/251 (0%)	1/283 (0%)
- Postoperative hemorrage	1/251 (0%)	0/283 (0%)
- Pressure ulcer	1/251 (0%)	4/283 (1%)
- Pruritus	0/251 (0%)	1/283 (0%)
- Pyloric stenosis	1/251 (0%)	0/283 (0%)
- Recurrent tetanus	0/251 (0%)	1/283 (0%)
- Rhabdomyolysis	0/251 (0%)	1/283 (0%)
- Sinus bradycardia	0/251 (0%)	2/283 (1%)
- Sinusitis	0/251 (0%)	1/283 (0%)
- Skin allergy	6/251 (2%)	9/283 (3%)
- Spinal disc herniation	1/251 (0%)	0/283 (0%)
- Subcutaneous emphysema	0/251 (0%)	1/283 (0%)
- Torticolis	1/251 (0%)	0/283 (0%)
- Tracheal hemorrhage	13/251 (5%)	3/283 (1%)
- Tracheal stenosis	0/251 (0%)	1/283 (0%)
- Upper gastrointestinal hemorrhage	11/251 (4%)	11/283 (4%)
- Upper respiratory infection	3/251 (1%)	1/283 (0%)
- Urinary retention	2/251 (1%)	1/283 (0%)
- UTI	20/251 (8%)	34/283 (12%)
- Ventilator associated pneumonia	28/251 (11%)	36/283 (13%)
- Viral infection	0/251 (0%)	2/283 (1%)
- Voice alteration	0/251 (0%)	3/283 (1%)
- Wound bleed	2/251 (1%)	0/283 (0%)
- Wound infection	2/251 (1%)	1/283 (0%)

Table S18 All adverse events (Intrathecal intervention per-protocol population)

Characteristic	Intrathecal treatment (N=283)		Sham procedure (N=324)		
	n	Summary statistic	n	Summary statistic	P-value
Adverse Event	231		277		0.24
- Acute renal failure		1/231 (0%)		3/277 (1%)	
- Adrenal insufficiency		1/231 (0%)		2/277 (1%)	
- Anemia		1/231 (0%)		9/277 (3%)	
- Appendicitis		0/231 (0%)		1/277 (0%)	
- Arterial thromboembolism		1/231 (0%)		0/277 (0%)	
- arthralgia		2/231 (1%)		1/277 (0%)	
- Arthralgia		0/231 (0%)		1/277 (0%)	
- Arthritis		4/231 (2%)		1/277 (0%)	
- Aspiration pneumonia		2/231 (1%)		6/277 (2%)	
- Atrial fibrillation*		0/231 (0%)		2/277 (1%)	
- Atrial thrombosis		1/231 (0%)		0/277 (0%)	
- Back pain		1/231 (0%)		1/277 (0%)	
- Blood stream infection		8/231 (3%)		8/277 (3%)	
- Bruising		1/231 (0%)		0/277 (0%)	
- Cardiac arrest		0/231 (0%)		3/277 (1%)	
- Cellulitis		1/231 (0%)		0/277 (0%)	
- Cerbral infarction		0/231 (0%)		1/277 (0%)	
- Cerebral hemorrhage		1/231 (0%)		0/277 (0%)	
- Chills		1/231 (0%)		0/277 (0%)	
- Cholangitis		0/231 (0%)		1/277 (0%)	
- Chronic venous insufficiency		1/231 (0%)		0/277 (0%)	
- Conjunctivitis		2/231 (1%)		3/277 (1%)	
- Constipation		19/231 (8%)		19/277 (7%)	
- Deep vein thrombosis		0/231 (0%)		3/277 (1%)	
- Dizziness		3/231 (1%)		1/277 (0%)	
- Dysphagia		1/231 (0%)		0/277 (0%)	
- Failed extubation		2/231 (1%)		0/277 (0%)	
- Fever		5/231 (2%)		0/277 (0%)	
- Gastritis		4/231 (2%)		1/277 (0%)	
- Gastrointestinal disorder		0/231 (0%)		1/277 (0%)	
- Headache		8/231 (3%)		0/277 (0%)	
- Heat stroke		0/231 (0%)		1/277 (0%)	
- Hematuria		1/231 (0%)		2/277 (1%)	
- Hemorrhoids		2/231 (1%)		1/277 (0%)	
- Hiccup		1/231 (0%)		0/277 (0%)	

Characteristic	Intrathecal treatment (N=283) n	Summary statistic	Sham procedure (N=324) n	Summary statistic	P-value
- Hospital acquired pneumonia		2/231 (1%)		2/277 (1%)	
- Hyperglycaemia		1/231 (0%)		0/277 (0%)	
- Hypernatremia		0/231 (0%)		2/277 (1%)	
- Hypertension		0/231 (0%)		1/277 (0%)	
- Hypoalbuminemia		0/231 (0%)		1/277 (0%)	
- Hypocalcemia		13/231 (6%)		17/277 (6%)	
- Hypokalemia		19/231 (8%)		30/277 (11%)	
- Hypomagnesemia		3/231 (1%)		4/277 (1%)	
- Hyponatremia		13/231 (6%)		13/277 (5%)	
- Hypotension		1/231 (0%)		6/277 (2%)	
- Insomnia		1/231 (0%)		0/277 (0%)	
- Low weight, muscle wasting		1/231 (0%)		2/277 (1%)	
- Myalgia		0/231 (0%)		1/277 (0%)	
- Myocardial ischaemia		2/231 (1%)		1/277 (0%)	
- Myocarditis		0/231 (0%)		1/277 (0%)	
- Nausea		1/231 (0%)		0/277 (0%)	
- Neuralgia		0/231 (0%)		1/277 (0%)	
- Nose bleed		4/231 (2%)		2/277 (1%)	
- Oral infection		3/231 (1%)		1/277 (0%)	
- Otitis		1/231 (0%)		2/277 (1%)	
- pharyngitis		1/231 (0%)		0/277 (0%)	
- Phlebitis		2/231 (1%)		6/277 (2%)	
- Platelet count decreased		0/231 (0%)		2/277 (1%)	
- Pleural abscess		0/231 (0%)		1/277 (0%)	
- Pneumothorax		0/231 (0%)		1/277 (0%)	
- Postoperative hemorrage		1/231 (0%)		0/277 (0%)	
- Pressure ulcer		1/231 (0%)		4/277 (1%)	
- Pruritus		0/231 (0%)		1/277 (0%)	
- Pyloric stenosis		1/231 (0%)		0/277 (0%)	
- Recurrent tetanus		0/231 (0%)		1/277 (0%)	
- Rhabdomyolysis		0/231 (0%)		1/277 (0%)	
- Sinus bradycardia		0/231 (0%)		2/277 (1%)	
- Sinusitis		0/231 (0%)		1/277 (0%)	
- Skin allergy		6/231 (3%)		6/277 (2%)	
- Spinal disc herniation		1/231 (0%)		0/277 (0%)	
- Subcutaneous emphysema		0/231 (0%)		1/277 (0%)	
- Torticolis		1/231 (0%)		0/277 (0%)	

Characteristic	Intrathecal treatment (N=283) n	Summary statistic	Sham procedure (N=324) n	Summary statistic	P-value
- Tracheal hemorrhage		11/231 (5%)		3/277 (1%)	
- Tracheal stenosis		0/231 (0%)		1/277 (0%)	
- Upper gastrointestinal hemorrhage		11/231 (5%)		11/277 (4%)	
- Upper respiratory infection		3/231 (1%)		1/277 (0%)	
- Urinary retention		2/231 (1%)		1/277 (0%)	
- UTI		20/231 (9%)		33/277 (12%)	
- Ventilator associated pneumonia		28/231 (12%)		36/277 (13%)	
- Viral infection		0/231 (0%)		2/277 (1%)	
- Voice alteration		0/231 (0%)		2/277 (1%)	
- Wound bleed		1/231 (0%)		0/277 (0%)	
- Wound infection		1/231 (0%)		1/277 (0%)	

Table S19 All adverse events (Intramuscular intervention including pre-hospital intramuscular antitoxin population)

Characteristic	Equine intramuscular (N=265) n	Equine intramuscular (N=265) Summary statistic	Antitoxin in previous hospital (N=140) [1] n	Antitoxin in previous hospital (N=140) [1] Summary statistic	Human intramuscular (N=228) n	Human intramuscular (N=228) Summary statistic	P-value
Adverse Event	224		123		187		0.32
- Acute renal failure		2/224 (1%)		1/123 (1%)		1/187 (1%)	
- Adrenal insufficiency		3/224 (1%)		0/123 (0%)		0/187 (0%)	
- Allergy		1/224 (0%)		0/123 (0%)		0/187 (0%)	
- Anemia		6/224 (3%)		3/123 (2%)		2/187 (1%)	
- Appendicitis		0/224 (0%)		1/123 (1%)		0/187 (0%)	
- Arterial thromboembolism		1/224 (0%)		0/123 (0%)		1/187 (1%)	
- arthralgia		1/224 (0%)		0/123 (0%)		2/187 (1%)	
- Arthralgia		0/224 (0%)		0/123 (0%)		1/187 (1%)	
- Arthritis		0/224 (0%)		2/123 (2%)		3/187 (2%)	
- Aspiration pneumonia*		5/224 (2%)		1/123 (1%)		2/187 (1%)	
- Atrial fibrillation		2/224 (1%)		0/123 (0%)		0/187 (0%)	
- Atrial thrombosis		0/224 (0%)		0/123 (0%)		1/187 (1%)	
- Back pain		0/224 (0%)		1/123 (1%)		1/187 (1%)	
- Blood stream infection		7/224 (3%)		5/123 (4%)		5/187 (3%)	
- Bruising		1/224 (0%)		0/123 (0%)		0/187 (0%)	
- Cardiac arrest		1/224 (0%)		1/123 (1%)		1/187 (1%)	
- Cellulitis		0/224 (0%)		1/123 (1%)		0/187 (0%)	
- Cerbral infarction		1/224 (0%)		0/123 (0%)		0/187 (0%)	
- Cerebral hemorrhage		0/224 (0%)		0/123 (0%)		1/187 (1%)	
- Chills		0/224 (0%)		0/123 (0%)		1/187 (1%)	
- Cholangitis		0/224 (0%)		0/123 (0%)		1/187 (1%)	
- Chronic venous insufficiency		1/224 (0%)		0/123 (0%)		0/187 (0%)	
- Conjunctivitis		3/224 (1%)		1/123 (1%)		1/187 (1%)	
- Constipation		17/224 (8%)		5/123 (4%)		16/187 (9%)	
- Creatinine increase		0/224 (0%)		0/123 (0%)		1/187 (1%)	
- Deep vein thrombosis		2/224 (1%)		1/123 (1%)		1/187 (1%)	
- Dizziness		1/224 (0%)		1/123 (1%)		2/187 (1%)	
- Dysphagia		0/224 (0%)		0/123 (0%)		1/187 (1%)	
- facial nerve disorder		1/224 (0%)		0/123 (0%)		0/187 (0%)	
- Failed extubation		1/224 (0%)		0/123 (0%)		1/187 (1%)	
- Fever		1/224 (0%)		1/123 (1%)		3/187 (2%)	
- Gastritis		4/224 (2%)		0/123 (0%)		1/187 (1%)	

Characteristic	Equine intramuscular (N=265)		Antitoxin in previous hospital (N=140) [1]		Human intramuscular (N=228)		P-value
	n	Summary statistic	n	Summary statistic	n	Summary statistic	
- Gastrointestinal disorder		0/224 (0%)		1/123 (1%)		0/187 (0%)	
- Headache		2/224 (1%)		0/123 (0%)		6/187 (3%)	
- Heat stroke		1/224 (0%)		0/123 (0%)		0/187 (0%)	
- Hematuria		1/224 (0%)		0/123 (0%)		2/187 (1%)	
- Hemorrhoids		1/224 (0%)		2/123 (2%)		0/187 (0%)	
- Hiccup		0/224 (0%)		0/123 (0%)		1/187 (1%)	
- Hospital acquired pneumonia		0/224 (0%)		3/123 (2%)		1/187 (1%)	
- Hyperglycaemia		0/224 (0%)		1/123 (1%)		0/187 (0%)	
- Hypernatremia		1/224 (0%)		1/123 (1%)		0/187 (0%)	
- Hyperpyrexia		0/224 (0%)		0/123 (0%)		1/187 (1%)	
- Hypertension		0/224 (0%)		0/123 (0%)		1/187 (1%)	
- Hypoalbuminemia		1/224 (0%)		0/123 (0%)		0/187 (0%)	
- Hypocalcemia		12/224 (5%)		11/123 (9%)		9/187 (5%)	
- Hypokalemia		19/224 (8%)		18/123 (15%)		14/187 (7%)	
- Hypomagnesemia		4/224 (2%)		1/123 (1%)		3/187 (2%)	
- Hyponatremia		8/224 (4%)		10/123 (8%)		9/187 (5%)	
- Hypotension		5/224 (2%)		1/123 (1%)		1/187 (1%)	
- Insomnia		1/224 (0%)		0/123 (0%)		0/187 (0%)	
- Low weight, muscle wasting		3/224 (1%)		0/123 (0%)		0/187 (0%)	
- Myalgia		0/224 (0%)		0/123 (0%)		1/187 (1%)	
- Myocardial ischaemia		2/224 (1%)		0/123 (0%)		1/187 (1%)	
- Myocarditis		0/224 (0%)		1/123 (1%)		1/187 (1%)	
- Nausea		1/224 (0%)		0/123 (0%)		0/187 (0%)	
- Necrotic wound		0/224 (0%)		1/123 (1%)		0/187 (0%)	
- Neuralgia		1/224 (0%)		0/123 (0%)		0/187 (0%)	
- Nose bleed		3/224 (1%)		1/123 (1%)		3/187 (2%)	
- Oral infection		2/224 (1%)		0/123 (0%)		2/187 (1%)	
- Otitis		1/224 (0%)		0/123 (0%)		2/187 (1%)	
- pharyngitis		0/224 (0%)		1/123 (1%)		0/187 (0%)	
- Phlebitis		3/224 (1%)		2/123 (2%)		3/187 (2%)	
- Platelet count decreased		2/224 (1%)		0/123 (0%)		0/187 (0%)	
- Pleural abscess		0/224 (0%)		1/123 (1%)		0/187 (0%)	
- Pneumothorax		1/224 (0%)		0/123 (0%)		0/187 (0%)	
- Postoperative hemorrage		1/224 (0%)		0/123 (0%)		0/187 (0%)	
- Pressure ulcer		2/224 (1%)		2/123 (2%)		1/187 (1%)	
- Pruritus		0/224 (0%)		0/123 (0%)		1/187 (1%)	

Characteristic	Equine intramuscular (N=265) n	Summary statistic	Antitoxin in previous hospital (N=140) [1] n	Summary statistic	Human intramuscular (N=228) n	Summary statistic	P-value
- Pyloric stenosis		0/224 (0%)		0/123 (0%)		1/187 (1%)	
- Recurrent tetanus		1/224 (0%)		0/123 (0%)		0/187 (0%)	
- Rhabdomyolysis		0/224 (0%)		1/123 (1%)		0/187 (0%)	
- Sinus bradycardia		1/224 (0%)		0/123 (0%)		1/187 (1%)	
- Sinusitis		1/224 (0%)		0/123 (0%)		0/187 (0%)	
- Skin allergy		7/224 (3%)		2/123 (2%)		6/187 (3%)	
- Spinal disc herniation		1/224 (0%)		0/123 (0%)		0/187 (0%)	
- Subcutaneous emphysema		0/224 (0%)		0/123 (0%)		1/187 (1%)	
- Torticolis		1/224 (0%)		0/123 (0%)		0/187 (0%)	
- Tracheal hemorrhage		8/224 (4%)		1/123 (1%)		7/187 (4%)	
- Tracheal stenosis		0/224 (0%)		1/123 (1%)		0/187 (0%)	
- Upper gastrointestinal hemorrhage		7/224 (3%)		5/123 (4%)		10/187 (5%)	
- Upper respiratory infection		0/224 (0%)		4/123 (3%)		0/187 (0%)	
- Urinary retention		1/224 (0%)		0/123 (0%)		2/187 (1%)	
- UTI		23/224 (10%)		13/123 (11%)		18/187 (10%)	
- Ventilator associated pneumonia		29/224 (13%)		8/123 (7%)		27/187 (14%)	
- Viral infection		2/224 (1%)		0/123 (0%)		0/187 (0%)	
- Voice alteration		1/224 (0%)		2/123 (2%)		0/187 (0%)	
- Wound bleed		0/224 (0%)		1/123 (1%)		1/187 (1%)	
- Wound infection		1/224 (0%)		2/123 (2%)		0/187 (0%)	

[1]Antitoxin available in previous hospital was equine human antitoxin only.

Tables S20a-c Primary outcome: requirement for mechanical ventilation: without exclusions for early mechanical ventilation

Table S20a Intrathecal intervention intention-to-treat population (without any exclusions for early mechanical ventilation)

Antitoxin	No MV[1] (n = 140)	MV (n = 131)	RR^2 (95% CI^3)	P-value	OR^4 (95% CI^3)	P-value
Arm (N = 271)						
Sham procedure	66 (49%)	69 (51%)	—		—	
Intrathecal treatment	74 (54%)	62 (46%)	0.89 (0.69, 1.14)	0.36	0.80 (0.50, 1.29)	0.36

[1]MV = Mechanical ventilation; [2]RR = Relative risk; [3]CI = Confidence Interval; [4]OR = Odds ratio

Table S20b Intramuscular intervention intention-to-treat population (without exclusions for early mechanical ventilation)

Antitoxin	No MV[1] (n = 119)	MV (n = 98)	RR^2 (95% CI^3)	P-value	OR^4 (95% CI^3)	P-value
Arm (N = 217)						
Equine intramuscular	60 (56%)	48 (44%)	—		—	
Human intramuscular	59 (54%)	50 (46%)	1.03 (0.77, 1.39)	0.83	1.06 (0.62, 1.81)	0.83

[1]MV = Mechanical ventilation; [2]RR = Relative risk; [3]CI = Confidence Interval; [4]OR = Odds ratio

Table 20c Intramuscular intervention including pre-hospital intramuscular antitoxin population (without exclusions for early mechanical ventilation)

Antitoxin	No MV[1] (n = 140)	MV (n = 129)	RR^2 (95% CI^3)	P-value	OR^4 (95% CI^3)	P-value
Arm (N = 269)				0.095		0.095
Equine intramuscular	60 (56%)	48 (44%)	—		—	
Human intramuscular	59 (55%)	48 (45%)	1.01 (0.75, 1.36)	0.95	1.02 (0.59, 1.74)	0.95
Antitoxin at previous hospital[5]	21 (39%)	33 (61%)	1.37 (1.01, 1.85)	0.04	1.96 (1.02, 3.86)	0.05

[1]MV = Mechanical ventilation; [2]RR = Relative risk; [3]CI = Confidence Interval; [4]OR = Odds ratio. [5]Antitoxin at previous hospital was intramuscular equine origin only.

Table S21 Interaction between treatments including intramuscular antitoxin at previous hospital population (intention-to-treat populations)

	Antitoxin	No MV[1]	MV	OR[2] for MV	95% CI[3]	P-value
In Sham procedure group	N = 131					0.42
	Equine intramuscular	22 (44%)	28 (56%)	—	—	
	Human intramuscular	28 (57%)	21 (43%)	0.59	0.26, 1.30	0.19
	Equine intramuscular in previoushospital[4]	16 (50%)	16 (50%)	0.79	0.32, 1.92	0.60
In intrathecal treatment group	N = 130					0.003
	Equine intramuscular	38 (67%)	19 (33%)	—	—	
	Human intramuscular	31 (60%)	21 (40%)	1.35	0.62, 2.98	0.45
	Equine intramuscular in previoushospital[4]	5 (24%)	16 (76%)	6.40	2.15, 22.1	0.001
In human intramuscular group	N = 101					
	Sham procedure	28 (57%)	21 (43%)	—	—	
	Intrathecal treatment	31 (60%)	21 (40%)	0.90	0.41, 2.00	0.80
In equine intramuscular group	N = 107					
	Sham procedure	22 (44%)	28 (56%)	—	—	
	Intrathecal treatment	38 (67%)	19 (33%)	0.39	0.18, 0.85	0.02
Antitoxin in previous hospital group[4]	N = 53					
	Sham procedure	16 (50%)	16 (50%)	—	—	
	Intrathecal treatment	5 (24%)	16 (76%)	3.20	0.99, 11.7	0.06
Overall interaction	Human intramuscular* Intrathecal treatment			2.30	0.76, 7.06	0.14
	Equine intramuscular in previous hospital * Intrathecal treatment			8.15	1.98, 36.8	0.005

[1]MV = Mechanical ventilation; [2]RR = Relative risk; [3]CI = Confidence Interval. [4]Antitoxin available in previous hospital was intramuscular equine origin only.

Table S22 Baseline characteristics of patients treated at previous hospital antitoxin and with intrathecal treatment only

	Equine intramuscular (N=57)		Antitoxin in previous hospital[1] (N=21)		Human intramuscular (N=52)	
Characteristic	n	Summary statistic	n	Summary statistic	n	Summary statistic
Age [years]g	57	49.0 (38.0, 61.0)	21	51.0 (41.0, 55.0)	52	43.0 (36.5, 57.5)
Female sex	57	13/57 (22.8%)	21	3/21 (14.3%)	52	6/52 (11.5%)
BMI [kg/m2]	57	21.8 (20.3, 23.4)	21	21.6 (20.2, 24.6)	52	21.0 (19.1, 22.7)
Days in ICU [days]*	57	10.0 (7.0, 18.0)	21	19.0 (15.0, 22.0)	52	12.5 (8.0, 21.2)
Comorbidity						
- Myocardial infarction	56	1/56 (1.8%)	21	0/21 (0.0%)	51	0/51 (0.0%)
- Chronic pulmonary	56	0/56 (0.0%)	21	0/21 (0.0%)	51	1/51 (2.0%)
- Mild liver	56	17/56 (30.4%)	21	4/21 (19.0%)	51	20/51 (39.2%)
- Diabetes with chronic	56	1/56 (1.8%)	21	0/21 (0.0%)	51	1/51 (2.0%)
- Severe liver	56	3/56 (5.4%)	21	1/21 (4.8%)	52	0/52 (0.0%)
- Cerebrovascular	56	1/56 (1.8%)	21	1/21 (4.8%)	51	0/51 (0.0%)
- Peptic ulcer	56	2/56 (3.6%)	21	0/21 (0.0%)	52	1/52 (1.9%)
- Diabetes	56	5/56 (8.9%)	21	0/21 (0.0%)	52	1/52 (1.9%)
- Severe kidney	56	2/56 (3.6%)	21	0/21 (0.0%)	52	1/52 (1.9%)
- Malignancy	56	0/56 (0.0%)	21	0/21 (0.0%)	52	1/52 (1.9%)
- Dementia	56	0/56 (0.0%)	21	0/21 (0.0%)	52	1/52 (1.9%)
- Acute Renal failure	57	2/57 (3.5%)	21	0/21 (0.0%)	52	0/52 (0.0%)
- Elective surgery <30 days	57	1/57 (1.8%)	21	0/21 (0.0%)	52	0/52 (0.0%)
- Emergency surgery <30 days	57	4/57 (7.0%)	21	1/21 (4.8%)	52	4/52 (7.7%)
Duration of illness [days]	57	3.0 (3.0, 5.0)	21	3.0 (2.0, 4.0)	52	3.0 (2.0, 5.2)
Incubation period [days]	44	8.0 (6.0, 13.2)	17	7.0 (6.0, 14.0)	42	9.0 (6.2, 13.5)
Period of onset [hours]	46	48.0 (24.0, 72.0)	21	24.0 (24.0, 48.0)	44	24.0 (24.0, 72.0)
Ablett score*	57		21		52	
- I		14/57 (24.6%)		0/21 (0.0%)		9/52 (17.3%)
- II		40/57 (70.2%)		18/21 (85.7%)		39/52 (75.0%)
- III		3/57 (5.3%)		3/21 (14.3%)		4/52 (7.7%)
ASA score*	57		21		52	

	n	Equine intramuscular (N=57)	n	Antitoxin in previous hospital[1] (N=21)	n	Human intramuscular (N=52)
- 1		33/57 (57.9%)		14/21 (66.7%)		24/52 (46.2%)
- 2		21/57 (36.8%)		7/21 (33.3%)		25/52 (48.1%)
- 3		3/57 (5.3%)		0/21 (0.0%)		3/52 (5.8%)
APACHE II score*	57	3.0 (2.0, 7.0)	21	3.0 (2.0, 6.0)	52	4.0 (1.0, 7.0)
SOFA score*	57		21		52	
- 0		50/57 (87.7%)		18/21 (85.7%)		43/52 (82.7%)
- 1		7/57 (12.3%)		2/21 (9.5%)		6/52 (11.5%)
- 2		0/57 (0.0%)		1/21 (4.8%)		2/52 (3.8%)
- 3		0/57 (0.0%)		0/21 (0.0%)		1/52 (1.9%)
Tetanus Severity Score*	57	0.0 (-3.0, 3.0)	21	0.0 (-2.0, 5.0)	52	2.0 (-3.0, 5.0)

- n = number of patients included in that summary statistic.

- Values in the form of X (A, B) are medians followed by the 25th and 75th percentiles in parentheses.

[1] Antitoxin available in previous hospital was intramuscular equine origin only

* Prognostic indicators on admission to hospital. Incubation period is the period from wound to first symptom; period of onset is the period from first symptom to first spasm; Ablett score: Grade I: no spasms; II tetanus with spasms not interfering with respiration; III severe spasms interfering with respiration (3); APACHE II (4), Sequential Organ Failure Score (5), Tetanus Severity Score (2). Antitoxin at previous hospital was intramuscular equine origin only.

Table S23 a&b Time from ICU admission to intervention: intention-to-treat and per-protocol populations

Table S23a: Median (IQR) time from ICU admission to intrathecal and intramuscular intervention for intrathecal populations (intention-to-treat and per-protocol populations)

Variable	Intention-to-treat population (N=271)		Per-protocol population (N=264)	
Arm	Sham procedure	Intrathecal treatment	Sham procedure	Intrathecal treatment
Time from ICU admission to intrathecal intervention (hours)	3.4 (2.1, 6.2)	3.5 (2.4, 7.4)	3.4 (2.1, 6.2)	3.5 (2.4, 7.3)
Time from ICU admission to intramuscular intervention (hours)*	1.7 (0.5, 2.3)	1.8 (1.0, 2.5)	1.7 (0.5, 2.3)	1.8 (1.1, 2.5)

*Includes the estimated time of intramuscular antitoxin given at previous hospital (based on an assumption that antitoxin was given shortly before hospital transfer)

Table S23b: Median (IQR) time from ICU admission to intrathecal and intramuscular intervention for intramuscular populations (intention-to-treat, per-protocol, and pre-hospital antitoxin populations).

	Intention-to-treat population (N = 217)		Per-protocol population (N = 215)		Equine IM pre hospital* (N=54)
Arm	Equine IM	Human IM	Equine IM	Human IM	
Time from ICU admission to intrathecal intervention (hours)	3.2 (2.0, 4.8)	3.8 (2.3, 8.3)	3.2 (2.0, 4.8)	3.8 (2.3, 8.3)	4.0 (2.8, 8.8)
Time from ICU admission to intramuscular intervention (hours)	2.1 (1.4, 2.9)	1.9 (1.5, 2.4)	2.1 (1.4, 2.9)	1.9 (1.5, 2.4)	-5.3 (-9,3, -3.5)

*Estimated time only – based on an assumption that antitoxin was given shortly before hospital transfer

Figure S22 Histogram showing numbers of males and females in whole study population

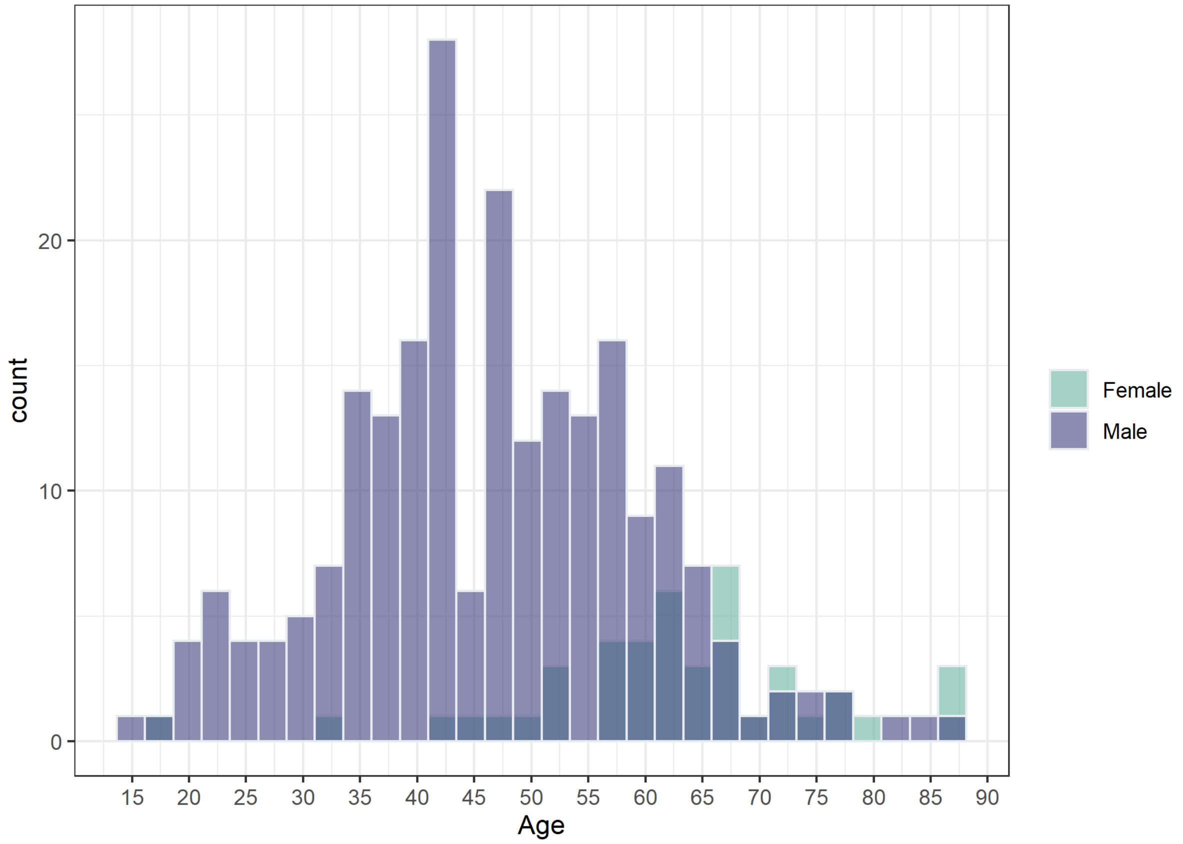

Overall, mean (SD) age was 49.1 (14.5). Mean (SD) age of men was 46.5 (13.2) years, whereas for women it was 62.4 (13.5) years.

Figure S23 Flow diagram showing intrathecal population only

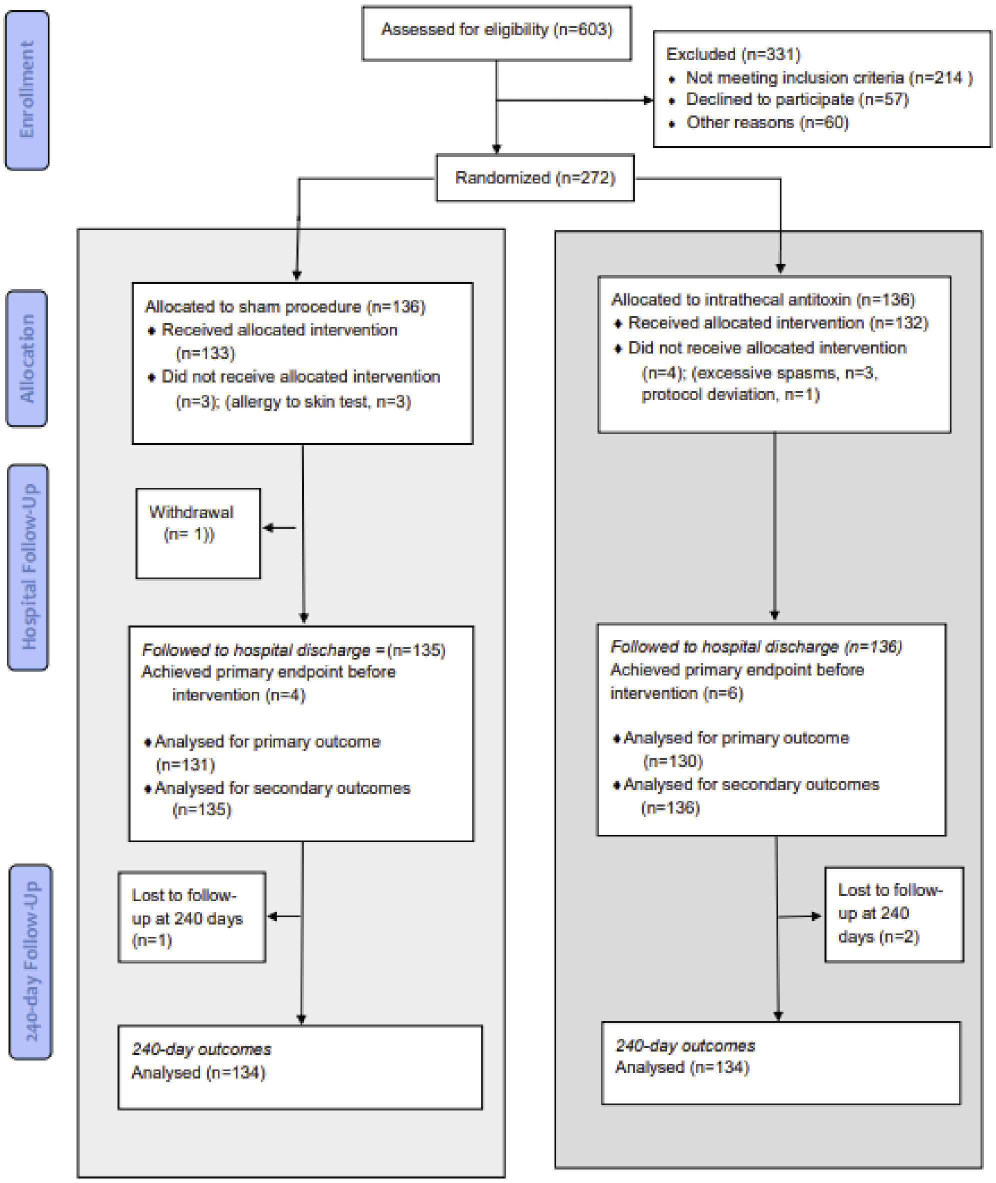

Figure S24 Flow diagram showing intramuscular population only

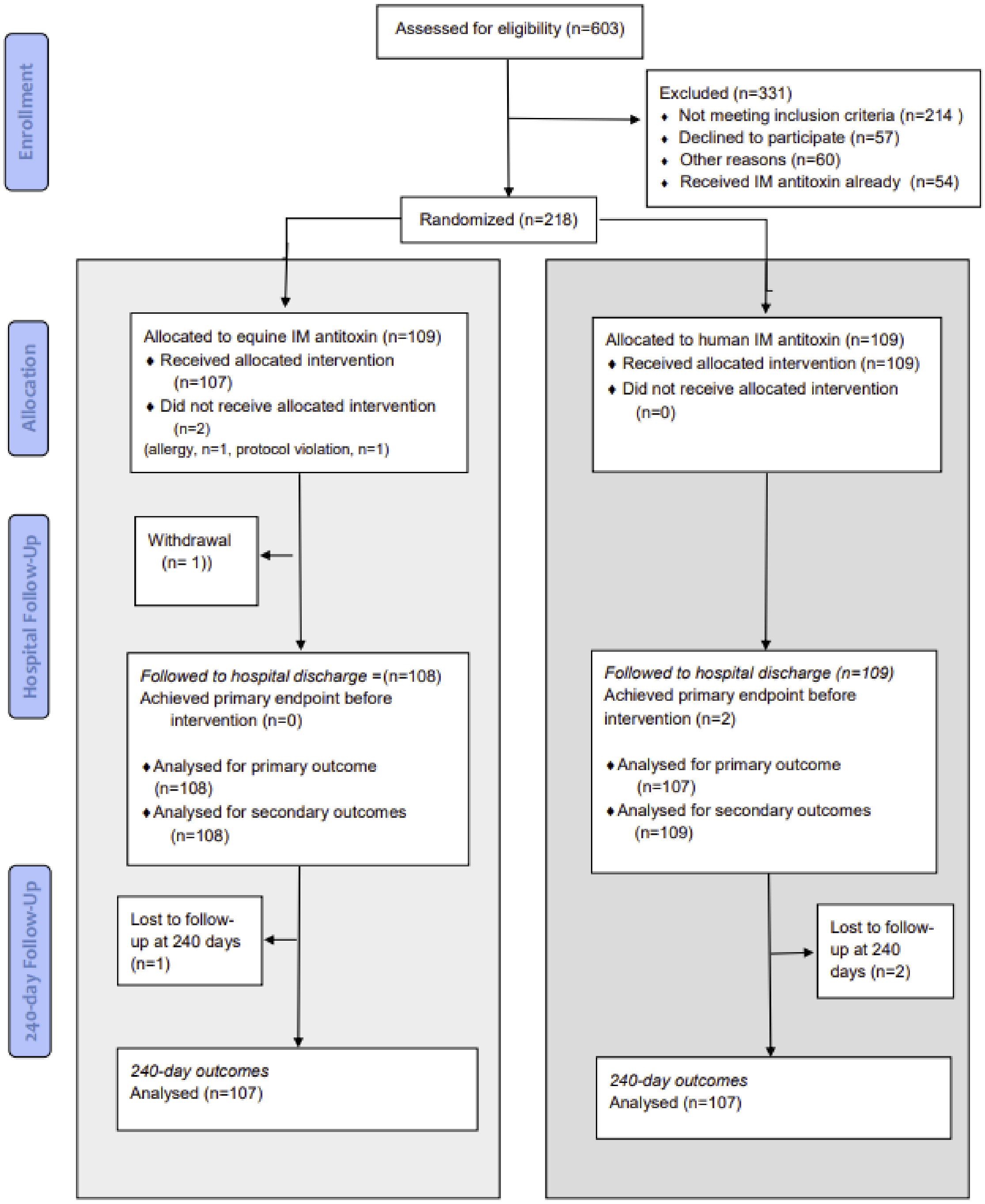

Statistical Analysis Plan

Revision History of Protocol and Statistical Analysis Plan

	Document number	Date	Details
**Protocol**	03TS OxTREC 17-16 Protocol EN V2.1 03NOV16	November 2016	Original protocol approved by ethics committees: Oxford Tropical Research Ethics Committee, Hospital for Tropical Diseases Ethics Committee and Ministry of Health Vietnam.
	03TS OxTREC 17-16 Protocol EN V3.0 01NOV17	November 2017	Protocol revision: The exclusion of patients with prior intramuscular antitoxin was removed (phase 2, section 4.3). Since starting recruitment we discovered that a higher than expected proportion of patients have already received intramuscular antitoxin before arriving at our hospital. This has not only led to lower than expected recruitment rates but also meant that the current study design fails to address the relevant clinical question in Vietnam. This revision was approved by all ethics committees and the Ministry of Health Vietnam.
**Statistical Analysis Plan**	03TS V1.1 217.9.20	17th September 2020	Original Statistical analysis plan approved by PI and Study statistician prior to analysis of trial data
	03TS V1.2 219.9.20	18th September 2020	Minor textual changes. For mortality, follow-up is set at 240 days for those that didn't die.
	03TS V1.3 28.10.20	28th October 2020	Typographical error in TSS changed (yes=4 and No=0)
	03TS V1.4 11.11.20	11th November 2020	Typographical error in APACHE II scoring changed
	03TS V1.5 19.11.20	19th November 2020	Clarification that ventilator associated pneumonia is only assessed in those ventilated

Statistical Analysis Plan for 03TS Intrathecal Immunoglobulin for Treatment of Adult Patients With Tetanus: a Randomized Controlled 2x2 Factorial Trial. NCT NCT02999815 v 1.5

This analysis plan was written by Ronald Geskus and Louise Thwaites

This document details the final analysis for the randomised controlled clinical trial OUCRU 03TS conducted at the Hospital for Tropical Diseases, Ho Chi Minh City as outlined in the trial protocol version 3.3 November 2017. The study was a superiority trial investigating whether additional intrathecal antitoxin is beneficial in adult patients with tetanus. This document contains detailed definitions of the endpoints. Deviations from the original protocol are noted.

This analysis plan was written before performing the analyses with the un-blinded treatment allocation. All changes made in the analyses after unblinding will be documented. The final analysis will be performed using the R version for Windows as specified in the final report.

Statistical adjustment for multiplicity testing will not be deployed, rather interpretation of results will consider this issue.

DATA SOURCES

The data-source for this analysis is the CliRes study database 03TS which contains multiple tables (eg. **ENR** contains patients' enrollment information, **VENT** contains medical ventilation history etc). The study Data Management Plan, Standard Operating Procedures include further information.

In this analysis plan, we refer to variables within tables by separating them by a dot, e.g. **VENT**.TRACHE refers to the variable TRACHE in table **VENT** and indicates yes or no whether a patient had a tracheostomy.

TRIAL DESIGN AND SAMPLE SIZE

Trial design

2x2 factorial design, blinded, randomized controlled trial of intrathecal immunoglobulin for treatment of tetanus in Vietnam. Randomisation is 1:1:1:1 to the four treatment arms in the 2x2 factorial trial (intrathecal treatment and human intramuscular treatment, intrathecal treatment and equine intramuscular treatment, sham procedure and human intramuscular treatment, sham procedure and equine intramuscular treatment). Randomization is based on a computer-based randomization list using block randomization with variable blocks lengths of 8 and 12 without stratification.

Sample size

The target sample size for this trial is 272 subjects. This sample size was calculated to detect an absolute risk reduction for mechanical ventilations due to intrathecal treatment by 17% (from 45% to 28%) with 80% power at the two-sided 5% significance level: (250 subjects are required but to account for some protocol violations and losses to follow-up, the trial will randomize a total of 272 patients).

Blinding

Treatment allocation were concealed from the investigators, study physicians, study nurses and attending ward staff throughout the study and hence was single blinded. Personnel involved in data entry and data checking were also blinded.

ANALYSIS POPULATIONS

There are five main populations defined:

1. **The intrathecal intention to treat (IT-ITT) population** consists of all patients who have been randomised to the trial. (derived as **ENR**.RANDTC with a date, ie not missing data). Analysis will be according to the randomized treatment arm (from randomization list).
2. **The intramuscular intention to treat (IM-ITT) population** consists of all patients who have been randomised to the trial and **did not** receive IM antitoxin before arrival at HTD (they were not excluded in the protocol). (derived as **ADM**.PREHTIG =N). Analysis will be according to the randomized treatment arm (from randomization list).

3. The **Intrathecal per-protocol population (IT-PP)** consists of all patients who received the allocated intrathecal treatment. Excluded individuals will be supplied as a separate list. Analysis will be according to the randomized treatment arm (from randomization list).
4. The **Intramuscular per-protocol population (IM-PP)** consists of all patients in the IM-ITT population who received the allocated intramuscular treatment. Analysis will be according to the randomized treatment arm. Excluded individuals will be supplied as a separate list. Analysis will be according to the randomized treatment arm (from randomization list).
5. The **All Intramuscular population** (IM-ALL) consists of all patients who received intramuscular antitoxin, including those who received it at a previous hospital. Those receiving antiotixin at a previous hospital will be analyzed as a separate equine antitoxin group. (Derived as **ADM**.PREHTIG =Y). This group was not mentioned in the protocol.
6. Subjects recruited in the pilot phase will not be included in any of the analyses (Subject IDs P01-P05

STUDY FLOW AND COMPLETENESS OF FOLLOW-UP

We will summarize the following quantities related to follow-up duration by treatment arm:

Number screened (Supplied on separate list); Reasons for ineligibility (supplied as separate list)

Number randomised (**ENR**.RANDTC not blank)

Number of subjects who completed of study in hospital (**COMPL**.COMPLETE =1)

Number of subjects who withdrew from study (**COMPL**.COMPLETE = 3)

Number of subjects available for Follow up at 240 days (**FU**.CONTACT = Y)

CALCULATION OF PRIMARY ENDPOINTS

Primary Endpoint: Requirement for mechanical ventilation during ICU stay (**VENT**. VENSTART=Y) . Patients requiring mechanical ventilation before study interventions will be excluded from primary endpoint analysis:

IIndividuals to be removed will be supplied as a separate list.

SECONDARY ENDPOINTS

- Duration of ICU stay in **days (ADM.ICUDISDTC -ADM.ICUDTC)**
- Duration of hospital stay in days (**ADM**.DISCDTC – **ADM**.ADMDTC)
- Duration of mechanical ventilation

  Only in patients with mechanical ventilation: **VENT.**VENSTART = Y,

  If **VENT**.OVER1VENEPISODE = N, duration is calculated as **VENT**.DATEVENSTOP – **VENT**.VENSTARTDATE

  if **VENT**.OVER1VENEPISODE = Y, duration is calculated as **VENT_VENTILATION**.DATEVENEND – **VENT**.VENSTARTDATE

- In hospital mortality (**ADM**.OUTCOME =1 or 2)
- mortality until 240 days (**FU**.PATSTAT = 1) and/or (**ADM**.OUTCOME =1 or 2) [Date of death = **FU**.DATEDEATH]
- 240 day disability (**FU**.RANKI)
- Probability of Ventilator Associated Pneumonia (**VENT**.VAP = Y). (Not the rate, as is mentioned in protocol) in patients ventilated (**VENT**.VENSTART =1)
- Probability of microbiologically confirmed Ventilator associated pneumonia (**VENT**.ETA=Y). (Not the rate, as is mentioned in protocol) in patients ventilated (**VENT**.VENSTART =1; missing values are N if VENT.VAP =N)
- New antibiotic prescription during ICU stay (excluding antibiotics for tetanus or initial entry site infection) (**DAILY**_DAILY. ANTIBIO = Y and **DAILY**_DAILY. ANTIBIOINDICATE >1 and **ADM**.ICUDTC - **DAILY**_DAILY .ASSDTC> 1 (Not rate, as mentioned in protocol)
- Clinical syndrome of autonomic nervous system dysfunction (**VENT**.ANSD=Y) (Not rate, as mentioned in protocol)
- Total dose of pipecuronium during hospital stay for patients who are ventilated (**VENT**. VENSTART=Y) (sum of the values of **DAILY_DAILY.**PIPECURONIUM per patient over the days) (not mentioned in protocol)

- Duration of pipecuronium during hospital stay in patients who are ventilated (**VENT**. VENSTART=Y) (length of **DAILY_DAILY.**PIPECURONIUM per patient) (not mentioned in protocol). The value is zero for those that did not receive pipecuronium.

- Total dose of diazepam during hospital stay (**DAILY_DAILY**.DIAZEPAMIM, + **DAILY_DAILY**.DIAZEPAMORAL, (not mentioned in protocol)

- Total dose of midazolam during hospital stay (**DAILY_DAILY**.MIDAZOLAM) (not mentioned in protocol)

- Total dose of benzodiazepines during hospital stay [as diazepam equivalent dose] calculated as total **DAILY_DAILY**. DIAZEPAMIM, + **DAILY_DAILY**.DIAZEPAMORAL + (4.17*(**DAILY_DAILY**.MIDAZOLAM)) (sum of the values per patient; not mentioned in protocol)

- Total duration of benzodiazepines (**DAILY_DAILY**.DIAZEPAMIM, **DAILY_DAILY**.DIAZEPAMORAL , **DAILY_DAILY**.MIDAZOLAM)) (length of the column per patient; not mentioned in protocol).

- Daily maximum and minimum systolic blood pressure (**DAILY_DAILY.**MAXSBP **DAILY_DAILY.**MINSBP) during first 7 days in hospital (not mentioned in protocol)

- Daily maximum and minimum heart rate (**DAILY_DAILY.**MAXHR **DAILY_DAILY.**MINHR) during first 7 days in hospital (not mentioned in protocol)

- Cost of ICU stay (**COMP**.COSTICU) Costs will be corrected to USD by dividing by 22, 660.83 (the exchange rate on 1/1/2018 as shown on https://www.exchange-rates.org/Rate/USD/VND/1-1-2018 accessed 14/6/2019)"

- Cost of hospital stay (**COMP**.COSTTOTAL) Costs will be corrected to USD by dividing by 22, 660.83 (the exchange rate on 1/1/2018 as shown on https://www.exchange-rates.org/Rate/USD/VND/1-1-2018 accessed 14/6/2019)"

- Occurrence of adverse events (see below)

ANALYSIS

Primary endpoint: Requirement for mechanical ventilation

The main analysis is the comparison between intrathecal treatment vs. sham procedure (IT-ITT and IT-PP). The effect of the second randomized intervention will be analyzed separately (IM-

ITT, IM-PP and IM-ALL). Patients will be analyzed according to their randomized arm. All inference is based on likelihood ratio tests. All effect measures will be supplied with p-values and 95% confidence intervals.

For both analyses, requirement for mechanical ventilation is summarized as x/n (%) in each treatment group and compared between the groups based on a logistic regression model with the intervention as the only covariate (intrathecal treatment vs. sham procedure for IT-ITT and IT-PP, human vs equine intramuscular treatment for IM-ITT, IM-PP and IM-ALL) . As odds ratios from logistic regression are somewhat difficult to interpret, we will additionally estimate relative risk between the groups based on a binary regression model with a log-link rather than the logit link function used in logistic regression.

We test for interaction between both treatments using a logistic regression model and the IM-ITT population. We report the odds ratios for each level of the other intervention, irrespective of statistical significance of the interaction (protocol deviation). We repeat this analysis for the IM-ALL population, with three IM treatment groups and two IT groups.

We will assess heterogeneity of the treatment effect with the following pre-defined grouping variables (one at a time, not all together in a single model):

- The TSS (Tetanus Severity Score; computation in the appendix) (calculated based on information available prior to randomization only) modeled via restricted cubic splines with three knots.
- Age (**ENR**.AGE), using restricted cubic splines with three knots (protocol used categories).
- Pre-existing medical conditions: severe illness defined according to ASA physical state scale (**ADM**.ASA see Appendix 2 of protocol). It will be dichotomized 1 versus 2/3 (4 and 5 do not occur in this study)
- Antitoxin (**ENR**.ANTIOXIN) before hospital admission

This will be done for each intervention separately (IT-ITT, IT-PP, IM-PP, IM-ALL and IM-ITT), based on a logistic regression analysis. For the continuous interaction terms we plot the odds ratios for the range of the continuous variable in a figure.

Secondary endpoints

All analyses will be performed for the comparison of intrathecal vs. sham procedure (IT-ITT and IT-PP) and for the comparison of equine vs human antitoxin intramuscularly (IM-ITT, IM-PP and IM-ALL).

For duration of hospital stay, duration of ICU stay and duration of ventilation, in hospital death is treated as a competing event (protocol deviation). Those that were discharged palliatively are considered as in hospital deaths as well and are assumed to have died at discharge. For duration of ventilation, people who have ventilation stopped to allow them to go home to die will be considered as deaths as well. So if end of ventilation day = day of death or palliative discharge then we should consider this as a death for analysis. We will nonparametrically estimate the cause-specific cumulative incidence for each of the three event types and death, and we plot the results. We test for differences between the treatment groups using Gray's log-rank test. Contrary to the protocol, no Cox regression is performed.

For in hospital mortality, we compute the number of patients who died or were discharged palliatively during their stay in hospital and we fit a logistic regression model (protocol deviation).
Mortality up to 240 days will be visualized using Kaplan-Meier curves and arms will be compared using the log-rank test. We assume that individuals without death observed remained alive until day 240.
Neurological disability (as assessed by the ordinal Rankin scale) at 240 days will be compared between the two arms with a proportional odds logistic regression model (not mentioned in protocol). The result will be summarized as a cumulative odds ratio with corresponding 95% confidence interval and p-value.
Ventilator associated pneumonia, new antibiotic prescription during ICU stay and clinical syndrome of autonomic nervous system dysfunction will be analyzed using logistic regression. Daily maximum and minimum systolic blood pressure and heart rate during first seven days in hospital will be compared using a random effects model. We will consider a linear trend for fixed and random effect, but will add a quadratic term for both effects if it gives a better model fit ($p<0.10$).

The cumulative dose (pipecuronium, diazepam, midazolam, benzodiazepines) and duration (pipecuronium, benzodiazepines) outcomes and the cost outcomes may have a skewed distribution. Therefore we use the Box-Cox procedure with intervention arm as covariable to find a suitable transformation. If reasonable, we use the identity or log transformation. After the transformation, arms are compared using linear regression. As effect measure we report the difference in expected value on the transformed scale (and report the transformation we used). Since this may be hard to interpret, we also plot the distribution of the cost variable by intervention arm via histograms. We add to the histograms the mean value with 95% confidence interval for each intervention arm.

Adverse Events

Adverse events (AE) have been derived by study physicians who were blind to the treatment allocations. We consider "any adverse event" as well as each AE separately. Tables will be generated to summarize the proportion of individuals with the adverse event, tabulating adverse events by grade (I-IV) and whether these events were judged to be related or possibly related to the treatment intervention. [*AE.SAE DATA SHEET* **SAE_GRID_AE.**CTCAENAME, **SAE_GRID_AE.**CTCAEGRADE, POSRELUNREL , "POSREL" or "REL"]. The following events will be excluded from adverse event reporting *AE.SAE DATA SHEET:* **SAE_GRID_AE.**CTCAENAME = "Nasogastric tube"; "Urinary Catheter"; "Tracheostomy"; "Mechanical ventilation" and "ANSD"

Tables will be generated separately for severe adverse events for IT-ITT, IM-PP, IM-ITT, IM-PP and IM-ALL,[*AE.SAE DATA SHEET* **SAE_GRID_SAE.** SAECATEGORY]. Comparisons of the proportions will be done with the chi-square test for independence; if the expected number is <= 1 in at least one of the cells, Fisher's exact test is used. AE and SAE data will be supplied as a separate file [*AE.SAE DATA SHEET*].

OTHER DESCRIPTIVE ANALYSES

Summary of baseline characteristics

Baseline characteristic**s** will be summarized as median (1st and 3rd quartile, lowest and highest value) for numeric data and n (%) for categorical data. No formal statistical comparison of baseline characteristics between the two study arms will be performed.

The following baseline characteristics will be summarized:

a. Patient details: sex, age (**ENR.**AGE **ENR.**SEX), BMI (**ADM**.WEIGHT/(**ADM**.HEIGHT)^2)
b. Past medical history:
   1.
   ADM.HYPERTENSION, **ADM.**MYOCARDIALINFART, **ADM.**ANGINA, **ADM**.PERIVASCULAR, **ADM.**CHRONICPUL, **ADM**.CONNECTIVETISSUE, **ADM**.MILDLIVER, **ADM**.HEMIPLEGIA, **ADM**.DIAWITHCHRONIC, **ADM**.SEVERELIVER, **ADM**.AIDS, **ADM**.CARDIACFAILUREIII, **ADM**.CARDIACFAILUREIV, **ADM**.CEREBROVASCULAR, **ADM**.SEVERERESP, **ADM**.PEPTICULCER, **ADM**.DIABETES, **ADM**.SEVEREKIDNEY, **ADM**.MALIGNANCY, **ADM**.TUMOUR, **ADM**.DEMENTIA, **ADM**.COMORBIDITYOTH1, **ADM**.COMORBIDITYOTH2

2. Recent surgery **ADM**.ELECTIVESURGERY, **ADM**.EMERGENCYSURGERY

c. Patient history

- Duration of illness (**ADM.**TIMETOADM)
- Incubation period (**ADM**.INCUBATIONPERIOD)
- Period of onset (**ADM**.INCUPERIODONSET)
- Wound (**ADM.**WOUND 1=deep, 2 – superficial/other)
- Difficulty breathing on admission (**ADM**.DIFFBREATH)
- Ablett Score on admission (**ADM**.ABLETT, values I, II, III or IV)
- ASA Score (**ADM**.ASA, values 1,2 3 or 4)
- Maximum temperature during 1st day (**ADM**.MAXTEMP)
- Respiratory Rate (**ADM**.RESP)
- FiO2 (**ADM**.FIO2)
- SpO2 (**ADM**.SPO2)
- PAO2 (**ADM.**PAO2)
- PH (**ADM**.PH)
- Platelet count (**ADM**.PLT)
- White blood cell count (**ADM**.WBC)
- Haematorcrit (**ADM**.HCT)
- Max HR (**ADM**.MAXHR)
- Min HR (**ADM**.MINHR)
- Max SBP (**ADM.**MAXSBP)
- Worst DBP (**ADM**.WORSTDBP)
- Worst SBP (**ADM**.WORSTSBP)
- Vasopressors (**ADM**.VASO)
- Bilirubin (**ADM**.BILI)
- Sodium (**ADM**.NA)
- Potassium (**ADM**.K)
- Creatinine (**ADM**.CREAT)
- Acute Renal failure **(ADM**.RENALFAILURE)
- Specific severity scores will be calculated from the above variables (See Appendix for details)
  Tetanus Severity Score, SOFA score, APACHE II score

Treatment details

- Time from hospital admission to intramuscular antitoxin calculated from admission date and time (**ADM**.ADMDTC **ADM**.ADMTIME) and time and date of intrathecal procedure (**ADM**.DATEHTIG and **ADM**.TIMEHITG); [Excluding those with intramuscular anditoxin before admission (**ADM**.PREHTIG= Y) [for patient ID 03-057 onwards – before this answer is blank, which should be set to N]
- Time from hospital admission to intrathecal antitoxin calculated from admission date and time (**ADM**.ADMDTC **ADM**.ADMTIME) and Date intrathecal procedure (**ADM**.DATEIT); Time intrathecal procedure (**AMD**.TIMEIT);
- Proportion of patients with nasogastric tube (*AE.SAE DATA SHEET* **SAE_GRID_AE.** CTCAENAME = "Nasogastric tube"
- Proportion of patients with tracheostomy tube (*AE.SAE DATA SHEET* **SAE_GRID_AE.** CTCAENAME= "Tracheostomy"
- Proportion of patients with urinary catheter (*AE.SAE DATA SHEET* **SAE_GRID_AE.** CTCAENAME = "Urinary Catheter"

Appendix

Tetanus Severity Score (TSS)

The score is generated by summing the score for each variable.

Variable	Variable Name	Value	Score
Age	**ADM**.AGE	≤70	0
		71-80	5
		>80	10
Time 1st Symptom to admission	**ADM**.TIMETOADM	≤2	0
		3-5	-5
		>5	-6
Difficulty breathing	**ADM**.DIFFBREATH	Y	4
		N	0
	**ADM**.ASA	1	0

ASA score (co-existing med)		2	3
		3	5
		4	5
		5	9
Entry site	**ADM**.WOUND	1	7
		2	0
Highest systolic BP	**ADM**.MAXSBP	≤130	0
		131-140	2
		>140	4
Highest heart rate	**ADM**.MAXHR	≤100	0
		101-110	1
		111-120	2
		>120	4
Lowest heart rate	**ADM**.MINHR	≤110	0
		>110	-2
Highest temperature	**ADM**.MAXTEMP	≤38.5	0
		38.6-39	4
		39.1-40	6
		>40	8

SOFA Score

Variable	Variable Name	Value	Score
SpO2/FiO2	(**ADM**.SPO2)/(**ADM**.FIO2)	>301	0
		221-301	1
		142-220	2
		67-141	3
		<67	4
Mean arterial Pressure	(**ADM**.WORSTDBP)+((**ADM**.WORSTSBP-**ADM**.WORSTDBP)/3)	≥70	0
		<70	1
	**ADM**.VASO	Y	4*
Bilirubin	**ADM**.BILI	blank or <20	0

		20-32	1
		33-101	2
		102-204	3
		>204	4
Platelets	**ADM.**PLT	≥150	0
		100-149	1
		50-99	2
		20-49	3
		<20	4
Creatinine	**ADM.**CREAT	<110	0
		110-170	1
		171-299	2
		300-440	3
		>440	4
GCS	**ADM.**GCS	15	0
		13-14	1

All blanks score 0

* If VASO is Y then score 4 but don't score anything for WORSDBP (ie not 0 or 1 also)

APACHE II Score

All blanks score 0

Variable	Variable Name	Score	Points
Temperature	**ADM.**MAXTEMP	≥41	4
		39-40.9	3
		38.5-38.9	1
		36-38.4	0
Mean Arterial Pressure	(**ADM.**WORSTDBP)+ ((**ADM.**WORSTSBP- **ADM.**WORSTDBP)/3)	≥160	4
		130-159	3
		110-129	2
		70-109	0
		50-69	2
		40-49	3
		<40	4
Heart rate	**ADM.**MAXHR<br><br>OR<br><br>**ADM.**MINHR	≥180	4
		140-179	3
		110-139	2
		70-109	0
		55-69	2
		40-54	3

	[Highest score]	<40	4
Respiratory rate	**ADM**.RESP	≥50	4
		35-49	3
		25-34	1
		12-24	0
		10-11	1
		6-9	2
		<6	4
PaO2 if FiO2< 50%	**ADM.**PAO2		
		>70	0
		61-70	1
		55-60	2
		<55	4
pH	**ADM**.PH	≥7.7	4
		7.6-7.69	3
		7.5-7.59	1
		7.33-7.49	0
		7.25-7.32	2
		7.15-7.24	3
		<7.15	4
Sodium	**ADM**.NA	≥180	4
		160-179	3
		155-159	2
		150-154	1
		130-149	0
		120-129	2
		111-119	3
		<111	4
Potassium	**ADM**.K	≥ 7	4
		6-6.9	3
		5.5-5.9	1
		3.5-3.9	0
		3-3.4	1
		2.5-2.9	2
		<2.5	4
Creatinine	**ADM**.CREAT<br><br>[Double score if **ADM**.RENALFAILURE = Y]	≥210	4
		178-209	3
		133-177	2
		54 - 132	0
		<54	2
Haematocrit	**ADM**.HCT	≥60	4
		50-59.9	2
		46-49.9	1
		30-45.9	0

		20-20.9	2
		<20	4
White blood count	**ADM**.WBC	≥ 40	4
		20-39.9	2
		15-19.9	1
		3-14.9	0
		1-2.9	2
		<1	4
GCS	**ADM**.GCS		15 – (**ADM**.GCS)
Age	**ENR.**AGE	**<45**	0
		45-54	2
		55-64	3
		65-74	5
		>74	6
Surgery	**ADM**.ELECTIVESURGERY	Y	5
		N	0
	**ADM**.EMERGENCYSURGERY	Y	5
		N	0
Immune suppression	**ADM**.IMMUNOCOMPROMISED OR **ADM**.SEVERERESP OR **ADM**.CARDIACFAILUREIV OR **ADM**.DIAWITHCHRONIC OR **ADM**.SEVERELIVER	Y	5
		N	0

References

1. Common Terminology for Adverse Events Grading (CTAE) V5 . Available from: https://ctep.cancer.gov/protocolDevelopment/electronic_applications/ctc.htm

2. Thwaites CL, Yen LM, Glover C, Tuan PQ, Nga NTN, Parry J, et al. Predicting the clinical outcome of tetanus: the tetanus severity score. Trop Med Int Heal. 2006;11(3):279–87. A

3. Ablett J. Ablett JJL. Analysis and main experiences in 82 patients treated in the Leeds Tetanus Unit. In: Symposium on tetanus in Great Britain Leeds: Leeds General Infirmary. 1967. p. 1–10.

4. Knaus WA, Draper EA, Wagner DP, Zimmerman JE. APACHE II: a severity of disease classification system. Crit Care Med. 1985 Oct [cited 2014 Mar 11];13(10):818–29.

5. Ferreira FL, Bota DP, Bross A, Mélot C, Vincent JL. Serial evaluation of the SOFA score to predict outcome in critically ill patients. JAMA. 2001 Oct 10;286(14):1754–8.

6. American Society Anesthesiologists. ASA Physical Status Classification System . Available from: https://www.asahq.org/standards-and-guidelines/asa-physical-status-classification-system
